# 3^e^ Journées scientifiques sida du Sénégal (JSSS 2022) Diamniadio, Centre international de conférences Abdou Diouf, 1^er^ au 3 novembre 2022 Le sida en contexte de Covid-19 et de maladies émergentes : quelles stratégies pour réduire les inégalités?

**DOI:** 10.48327/mtsi.v4i1.2024.493

**Published:** 2024-02-23

**Authors:** Cheikh Tidiane NDOUR – DLSI, Halimatou Diop NDIAYE – LBV, Khoudia SOW – CRCF, M. Daouda DIOUF - ENDA-SANTÉ /OSC - AOC

**Affiliations:** 1. Sciences biologiques / laboratoire, Co-présidents : Pr Halimatou DIOP NDIAYE – LBV; Colonel Babacar FAYE – HMO.; 2. Prise en charge médicale adulte, Co-présidents : Pr Louise FORTES – DALAL JAMM; Dr Ndève Fatou Ngom GUEYE – CTA Dr Kouro BOUSSO – DLSI.; 3. Prise en charge médicale enfants et adolescents, Co-présidents : Pr Ndèye Ramatoulaye DIAGNE – HED, Dr Ndèye Fatou NGOM – UNICEF, Dr Geres V. Ahognon – RESEAU EVA.; 4. Santé publique, Co-présidents : Pr Adama FAYE – ISED, Dr Cheikh Bamba DIEYE – CNLS, Dr Jules Bashi BAGENDABANGA – FHI 360.; 5. Sciences sociales/politiques/droits humains, Co-présidents : Dr Khoudia SOW – CRCF, Dr Bernard TAVERNE – ANRS, IRD.; 6. Santé communautaire, Co-présidents : M. Ibrahima BA – BOKK YAKAAR, M. Innocent LAISON – AFRICASO, Mme Rokhaya NGUER – SWAA Sénégal

 

