# Supplementary material for: 3rd Scientific Aids Days of Senegal (JSSS 2022) Diamniadio, Abdou Diouf International Conference Center, November 1-3, 2022) AIDS in the context of Covid-19 and emerging diseases: what strategies are needed to reduce inequalities?
Source: Med Trop Sante Int. 2024 Feb 23;4(1):mtsi.v4i1.2024.493. [Article in French] doi: 10.48327/mtsi.v4i1.2024.493 (PMC11151933; doi:10.48327/mtsi.v4i1.2024.493)
Supplement: Related texts

# Research article: Éditorial

**Published:** 2024-02-23

**Authors:** Safiatou THIAM, Cheikh Bamba DIEYE GUEYE

**Affiliations:** Secrétaire exécutive du Conseil national de lutte contre le sida (CNLS); Présidente du Comité scientifique; Responsable Unité suivi évaluation et recherche; Conseil national de lutte contre le sida (CNLS); Co-président de la Commission santé publique

Les **Journées scientifiques sida du Sénégal (JSSS)** sont organisées tous les deux ans, depuis 2016, par le Conseil national de lutte contre le sida et ses partenaires. Elles rassemblent les principaux acteurs nationaux de la lutte contre le sida. Cette troisième édition s’est tenue du 1^er^ au 3 novembre 2022, au Centre international de conférence Abdou Diouf de Diamniadio (Sénégal).

L’objectif des JSSS est d’offrir un espace d’informations, d’échanges et de partage de bonnes pratiques et d’expériences dans la riposte au sida. Les communications orales ou affichées couvrent tous les champs disciplinaires : virologie, clinique, épidémiologie, santé publique et sciences sociales, en recherche fondamentale ou opérationnelle, académique ou communautaire. Il s’agit aussi de renforcer le plaidoyer pour l’engagement des leaders et des décideurs du privé et du public dans la réponse à l’épidémie.

Ces 3^e^ journées scientifiques font suite à deux années d’épidémie de Covid-19. La crise sanitaire qui a été difficilement vécue par les populations, notamment les plus vulnérables, dont les personnes vivant avec le VIH, n’a pas épargné les professionnels de santé qui ont tous dû faire face à d’énormes défis.

Les acteurs de la lutte contre le sida ont très tôt mis en place des stratégies d’adaptation et de résilience. Les acquis de la lutte contre le sida, notamment portés par les acteurs communautaires, ont bénéficié à la riposte au Covid-19. Des stratégies innovantes ont ainsi pu être mises en œuvre pour faciliter la continuité des services de prévention et de prise en charge liés au VIH.

Cette 3^e^ édition des JSSS était la première grande occasion, après deux années d’épidémie de Covid-19, pour tirer les leçons et améliorer nos capacités de réponse aux épidémies. Ce qui justifie le thème des JSSS3. Au-delà de la Covid-19, il a fallu intégrer les maladies émergentes.

Plus de 600 acteurs en provenance de toutes les régions du Sénégal ainsi que de pays et organisations partenaires ont participé à ce cadre de concertation et de partage pour une meilleure synergie des actions futures en vue de mettre fin à l’épidémie de sida d’ici à 2030.

Le Conseil national de lutte contre le sida est ravi de recevoir encore une fois, l’ensemble des partenaires : universités, organismes de recherche, organisations internationales, ONG, acteurs associatifs, entreprises du médicament, et acteurs de la société civile. Nous tenons à remercier tout particulièrement l’équipe du Centre régional de recherche et de formation à la prise en charge clinique de Fann – le CRCF – qui appuie l’organisation des Journées scientifiques sida du Sénégal depuis leur création. Ce numéro spécial sur la 3^e^ édition des JSSS est constitué par une sélection des résumés des communications effectuée par le Comité de rédaction de la revue *Médecine tropicale et santé internationale (MTSI)* que nous remercions.

**Figure 1:** 3^e^ Journées scientifiques sida du Sénégal (JSSS 2022), crédit photo : B. Taverne (IRD) 2022 3rd Scientific Aids Days of Senegal (JSSS 2022), photo credit: B. Taverne (IRD) 2022

**Mots-clés** : Journées scientifiques sida du Sénégal, JSSS, Adolescent, Adulte, API, Antirétroviraux (ARV), Autonomie, Autostigmatisation, Bisexualité, Charge virale, Co-infection, Comorbidités, Connaissances attitudes pratiques, Conseil et dépistage à l’initiative du prestataire (CDIP), Covid 19, Décentralisation, Dépistage, Diagnostic précoce, Échantillon naso-pharyngé, Échec thérapeutique, Échec virologique, Enfant, Épidémiologie, GeneXpert, Hépatite virale B, Hôpital, Herpès génital, Inhibiteurs enzymatiques, Inhibiteurs de la transcriptase inverse, Intégration, Jeunes leaders transformationnels (JLT), Ligne de traitement, Marrainage, Mbao, Mortalité, Guichet unique, Organisations identitaires, Pairs HSH, Papillomavirus humain (PVH), Parrainage des EVVIH, Personnes vivant avec le VIH (PvVIH), Point-of-Care (POC), Prestations de soins différenciés (PSD), Prévention, Ratio CD4/CD8, RT PCR, Santé sexuelle et reproductive (SSR), Sénégal, Séronégatif, Sida, Soins, Stratégie « Ndeye-Dické », Traitement antirétroviral (TAR), Tuberculose, Virus de l’immunodéficience humaine (VIH), Bignona, Fann, Guédiawaye, Mbour, Fatick, Kaolack, Saint-Louis, Thiès, Dakar, Casamance, Sénégal, Afrique subsaharienne

**Figure 2:** Affiche des 3^e^ Journées scientifiques sida du Sénégal (JSSS 2022) Poster of the 3rd Scientific Aids Days of Senegal (JSSS 2022)

# Research article: 161 - Suppression de la charge virale chez les patients co-infectés VIH-VHB sous traitement dans la cohorte SEN-B (septembre 2019 – novembre 2021) au Sénégal

**Published:** 2024-02-23

**Authors:** Messan Kodzo AKOTIA, Melissa Sandrine PANDI, Adrià RAMÍREZ MENA, Ousseynou NDIAYE, Moussa SEYDI, Gilles WANDELER

**Affiliations:** 1 Centre régional de recherche et de formation à la prise en charge clinique de Fann (CRCF), Dakar, Sénégal; 2 Faculté des sciences et technologies, Université Cheikh Anta Diop, Dakar; 3 Département des maladies infectieuses, Hôpital universitaire de Berne, Université de Berne, Berne, Suisse; 4 Institut de médecine sociale et préventive, Université de Berne; 5 Service des maladies infectieuses et tropicales du Centre hospitalier national universitaire (SMIT/CHNU) de Fann, Dakar; 6 Haute école des sciences de la santé, Université de Berne

## Contexte et objectif

La co-infection VIHVHB demeure un problème de santé publique majeur en Afrique subsaharienne. Au Sénégal, la prévalence de cette co-infection est de 3,2 %. Afin d’évaluer les bénéfices et les risques liés au traitement de cette infection, la mise en place d’études observationnelles au Sénégal constitue une urgence de santé publique d’où naquit le projet SEN-B dans lequel les patients infectés par le VIH et le VHB suivis au Centre de traitement ambulatoire (CTA) et au SMIT du CHNU de Fann constituent la cohorte SEN-B. L’objectif était d’évaluer la suppression de la charge virale VIH et VHB chez les patients co-infectés sous traitement dans la cohorte SEN-B.

## Méthodes

Il s’agissait d’une étude transversale, de septembre 2019 à novembre 2021, portant sur le plasma des patients co-infectés sous traitement dans la cohorte SEN-B ayant au moins 18 ans révolus avec un consentement libre, éclairé, écrit et signé. La quantification de la charge virale plasmatique du VIH-1 et du VHB a été réalisée grâce aux systèmes Cobas Ampliprep et Cobas Taqman 48, seuil de détection 20 copies/ml pour le VIH-1 et 20 UI/ml pour le VHB. Les analyses statistiques ont été faites à l’aide de RStudio R x64 4.0.1.

## Résultats

Sur 111 patients inclus, 59 étaient de sexe féminin et 52 de sexe masculin soit un sex-ratio Femme/Homme de 1,13. L’âge médian des patients était de 45,5 ans (IQR 37,25-54,00) avec des extrêmes allant de 18 à 76 ans. Avec une durée médiane du traitement de 5,37 ans (IQR 3,24-8,39), 105 des patients étaient sous une ligne contenant du ténofovir disoproxil fumarate (TDF) (87,57 % sous TDF/3TC et 12,43 % sous TDF/FTC). La proportion de suppression de la réplication du VIH-1 était de 75 % et celle du VHB de 81,1 % au sein de la cohorte. Parmi les 111 patients co-infectés, 107 patients (96,4 %) avaient réalisé une mesure de la charge virale (CV) VHB, 100 patients (90,1 %) une mesure de CV VIH-1 et en tout 99 des patients (89,19 %) avaient une mesure de CV à la fois VHB et VIH disponible. Ainsi sur ces 99 patients, 66 patients (73,3 %) avaient des CV VIH-VHB indétectables, 16 patients (16,16 %) avaient des CV VIH détectables et VHB indétectables, 9 patients (9,09 %) avaient à la fois des CV VIH-VHB détectables et 8 patients (8,08 %) avaient une CV VIH indétectable et une CV VHB détectable. Sur 6 patients en suspicion d’échec virologique, 3 ont été confirmés.

## Conclusion

Cette étude confirme l’efficacité de la mise sous traitement précoce des patients co-infectés sous un traitement antirétroviral contenant du TDF afin de leur assurer une bonne prise en charge clinique et d’éviter les complications liées au VHB. En perspective, nous aurons à identifier les causes liées à la non-suppression de la CV VHB chez les patients ayant une CV VIH indétectable.

# Research article: 81 - Étude de la séroprévalence du SARS-CoV-2 au sein de l’École supérieure polytechnique (ESP) de Dakar

**Published:** 2024-02-23

**Authors:** Clémence Stéphanie Chloé ANOUMBA NDIAYE, Fatou THIAM, Assane SENGHOR, Idy DIOP, Sarbanding SaNÉ, Seynabou COUNDOUL, Sophie Déli TENE, Djibaba DJOUMOI, Betty KANE, Cheikh Momar NGUER, Abou Abdallah Malick DIOUARA, Massamba DIOUF

**Affiliations:** 1 Groupe de recherche Biotechnologies appliquées & Bioprocédés environnementaux (GRBA-BE), École supérieure polytechnique (ESP), Université Cheikh Anta Diop, Dakar, Sénégal; 2 Laboratoire d’imagerie médicale et bio-informatique (LIMBI), École supérieure polytechnique (ESP), Université Cheikh Anta Diop, Dakar; 3 Laboratoire de santé publique, Institut d’odontologie et de stomatologie, Faculté de médecine, de pharmacie et d’odontologie, Université Cheikh Anta Diop, Dakar

## Description du problème

La crise sanitaire de Covid-19 eut des conséquences désastreuses sur plusieurs secteurs d’activité dont l’enseignement. En avril 2020, la Banque mondiale a estimé que les universités ont été fermées dans 175 pays avec plus de 220 millions d’étudiants impactés. Au Sénégal, malgré les stratégies de lutte, le virus continue à se propager par vagues poussant le gouvernement à mettre en place des stratégies de prévention et de surveillance afin d’enrayer la circulation du virus sur le territoire. Les études séro-épidémiologiques, moins coûteuses que celles moléculaires rentrent en droite ligne avec ces stratégies. Cependant au niveau des universités, lieux propices à l’apparition de clusters, il est constaté un manque criant de données de séroprévalence du SARS-CoV-2. Ainsi, cette étude a pour objectif de déterminer la séroprévalence du SARS-CoV-2 au sein de l’ESP, afin d’améliorer la stratégie de lutte anti-Covid-19.

## Activités réalisées et résultats obtenus

Nous avons mené une étude transversale monocentrique avec un échantillonnage stratifié en étudiants, personnel administratif technique et de service (PATS), personnel enseignant et de recherche (PER) au sein de la communauté de l’ESP. Les cibles ont été invitées à participer à l’étude par e-mail et à renseigner des données sociodémographiques, cliniques, d’exposition et de vaccination *via* un formulaire d’enquête. Après avoir signé un consentement éclairé, chaque participant a subi une ponction veineuse de 10 ml sur tube EDTA. Des tests ELISA ont été réalisés pour déterminer la séropositivité en IgM pour les infections récentes et courantes (kit Wantai SARS-CoV-2 IgM) et en IgG pour celles antérieures (kit Wantai SARS-CoV-2 IgG).

Un total de 616 participants a été dénombré dont 90,9 % d’étudiants, 2,6 % de PER et 6,5 % de PATS avec un sex-ratio F/H = 1,7. L’âge moyen était de 22,8 ans. Globalement, les résultats préliminaires montrent que 19,7 % des participants étaient positifs à la fois aux anti-IgM et anti-IgG du SARSCoV-2. La séroprévalence IgM montre un taux de 18,9 % avec une plus forte séroprévalence chez les hommes (25 %) comparée aux femmes (15,2 %). Selon les groupes de population, la séroprévalence IgM était de 18,5 %, 16,7 % et 23,8 % respectivement chez les étudiants, les PER et les PATS. Chez les personnes vaccinées, une plus faible séroprévalence IgM a été retrouvée chez celles ayant reçu deux doses de vaccin comparées à celles ayant reçu une dose.

La séroprévalence IgG était de 93 % pour un taux moyen de 21,1 U/ml. Le taux moyen d’IgG était plus élevé chez les personnes vaccinées comparées aux non vaccinées (97,3 % et 22,5 U/ml contre 92 % et 20,6 U/ml). Les personnes vaccinées avec le vaccin AstraZeneca présentent les taux moyens d’IgG les plus élevés.

## Leçons apprises et étapes futures

Ces résultats préliminaires montrent qu’une grande majorité des étudiants et du personnel de l’ESP a été exposée au SARS-CoV-2. D’autres analyses sont en cours afin de déterminer les proportions en anticorps neutralisants, et de faire des corrélations entre la séroprévalence IgM et IgG et les facteurs sociodémographiques et cliniques. À terme, dans une certaine mesure, les résultats attendus renseigneront sur la réponse immunitaire des participants face à la Covid-19.

# Research article: 139 - Recrudescence de la stigmatisation et de la discrimination dans les structures d’offre de service VIH chez les professionnelles du sexe, les hommes ayant des relations sexuelles avec d’autres hommes et les consommateurs de drogues injectables suivis par ENDA Santé dans le cadre du projet FEVE régional à Dakar, Mbour et Ziguinchor

**Published:** 2024-02-23

**Authors:** Ibrahima BÂ, Sidy Mokhtar NDIAYE, Mouhamadou BÂ, Floriane KALONJI, Nguissali TURPIN, Fatou Maria DRAMÉ, Daouda DIOUF

**Affiliations:** ENDA Santé

## Objectif

En Afrique et en particulier au Sénégal, nous notons ces dernières années une recrudescence de la stigmatisation et de la discrimination des professionnelles du sexe (PS), des hommes ayant des relations sexuelles avec d’autres hommes (HSH) et des consommateurs de drogues injectables (CDI) vivant avec le VIH, entraînée par la persistance d’un contexte socioculturel difficile selon diverses études au Sénégal (CNLS, 2018). Cette situation influe fortement sur leur accès aux services de prévention et de prise en charge du VIH, ce qui renforce davantage leur vulnérabilité sanitaire.

ENDA Santé met en œuvre un projet régional au Sénégal afin de contribuer à l’amélioration de la prise en charge des personnes vivant avec le VIH (PvVIH) et des personnes à risques face à l’épidémie du VIH et des infections sexuellement transmissibles (IST). C’est dans ce cadre qu’une recherche évaluative avait été réalisée en 2018 et 2021 auprès des bénéficiaires du projet dans le but d’apprécier l’évolution du degré de discrimination et de stigmatisation dans les structures d’offre de service.

## Méthodes

Il s’agit d’une évaluation quantitative à mi-parcours et à la fin du projet. Le procédé d’échantillonnage de type stratifié et proportionnel à la taille des bénéficiaires des zones d’intervention a été utilisé. Les données ont été collectées à l’aide de tablettes. Le traitement et l’analyse ont été réalisés par Stata 15.

## Résultats obtenus

En 2018, 733 personnes ont été interrogées, constituées de 46 % de PS, 32 % de HSH et 21 % de CDI. En 2021, ils étaient 746 soit 32 % de HSH, 46 % de PS et 22 % de CDI. En 2018, la moyenne d’âge était de 32 ans contre 35 ans en 2021. Plus de 48 % des personnes enquêtées en 2018 n’ont pas été scolarisées ou présentent un faible niveau d’instruction, contre 46 % en 2021. Environ la plupart (73 %) ont participé aux deux évaluations réalisées en 2018 et 2021. En 2021, 28 % des bénéficiaires des services d’ENDA Santé et qui fréquentent les structures d’offre de service ont affirmé avoir eu peur de se soigner parce que d’autres personnes pourraient découvrir leur statut, contre 19 % en 2018. 25 % ont renoncé à accéder aux soins par peur que d’autres personnes connaissent leur statut en 2021, contre 18 % en 2018. Environ 20 % des bénéficiaires en 2021 ont eu le sentiment qu’ils n’ont pas été bien traités dans les structures de santé, contre 10 % en 2018. Ceux qui ont eu le sentiment qu’un membre du personnel de santé a fait des remarques négatives ou des commérages se situent à 22 % en 2021, contre 11 % en 2018. Un peu plus de 16 % ont affirmé qu’un membre du personnel de santé a refusé de leur offrir des services du fait de leur profil en 2021, contre 5 % en 2018.

## Conclusion

L’augmentation de la stigmatisation et de la discrimination des personnes les plus touchées par le VIH constitue un sérieux obstacle dans l’accès aux soins de ces dernières et risque de porter un sérieux coup à plusieurs années de réponse au VIH au Sénégal ayant abouti à des résultats très satisfaisants. Dès lors, il est urgent que des stratégies innovantes soient mises en place à l’échelle nationale pour améliorer l’environnement de la réponse au VIH.

# Research article: 98 - Continuité des services de dispensation des antirétroviraux en période de Covid-19 : l’association Bokk Yakaar de Fatick met à contribution les motos « Jakarta »

**Published:** 2024-02-23

**Authors:** Ibrahima BÂ, Magatte KA, Oumy NDIAYE, Séverine CARILLON

**Affiliations:** 1 Bokk Yakaar, Fatick, Sénégal; 2 Centre régional de recherche et de formation à la prise en charge clinique de Fann (CRCF), Dakar, Sénégal

## Objectifs

L’avènement de la pandémie de Covid-19 au Sénégal en mars 2020 a bouleversé le dispositif médical de la prise en charge (PEC) des personnes vivant avec le VIH (PvVIH). Pour faire face aux restrictions de déplacement imposées par le Sénégal, l’association Bokk Yakaar de Fatick a mis en place un dispositif communautaire pour assurer la continuité des services.

L’objectif était de permettre aux PvVIH d’accéder à leurs antirétroviraux (ARV) à domicile et d’éviter les ruptures de traitement. Ce dispositif a permis en outre de limiter les déplacements des patients afin de réduire leurs risques de contact avec le coronavirus.

## Matériels et méthodes

Dès le premier cas de Covid-19 début mars 2020, l’association Bokk Yakaar a alerté les districts sanitaires sur la nécessité de fournir un traitement ARV multi-mois à leurs patients. Avec l’instauration de l’état d’urgence et la restriction des déplacements inter-urbains, l’association a mis à la disposition des sites de PEC des 7 districts sanitaires de la région ses motos ainsi que le carburant nécessaire pour l’acheminement des ARV et des kits d’hygiène dans les villages. Ainsi, les bénévoles et relais de Bokk Yakaar, en coordination avec les dispensateurs, ont identifié les localités où les patients ne pouvaient pas accéder aux postes de santé afin de leur livrer leurs médicaments. De plus, les bénévoles ont été formés sur la Covid-19 et équipés contre la maladie pour sensibiliser à domicile les familles. Des cartes de communication ont été mises à la disposition des médiateurs en santé et des assistants sociaux pour faire le suivi psychosocial à distance avec les patients.

## Résultats

Du 23 mars au 30 octobre 2020, l’association a pu acheminer à 203 patients leurs médicaments dans les villages (Fatick 6, Niakhar 10, Gossas 47, Dioffior 49, Passy 5 et Sokone 86). Au-delà de cette dispensation à domicile, à travers les médiateurs des autres régions, Bokk Yakaar a appuyé les patients bloqués dans ces localités pour obtenir leurs ARV grâce aux groupes WhatsApp.

En plus de la dispensation des ARV par les motos, les médiateurs et dispensateurs devaient appeler au moins 5 personnes pour leur expliquer les mesures barrières contre la Covid-19 et s’enquérir de leurs besoins psychologiques liés au VIH. Toutes ces informations ont ensuite été transcrites dans une fiche de suivi élaborée par Bokk Yakaar. L’association, en collaboration avec ses partenaires, a appuyé la fourniture aux PvVIH de kits d’hygiène et de produits alimentaires pour faire face au confinement et lutter efficacement contre la pandémie.

## Conclusion

Grâce à cette anticipation et au dispositif mis en place par l’association Bokk Yakaar, les PvVIH de la région n’ont pas eu de rupture de traitement. Cet exemple a inspiré le niveau national, à savoir la Division de lutte contre le sida et les IST (DLSI) et le Conseil national de lutte contre le sida (CNLS), pour accélérer la stratégie de dispensation communautaire des ARV au Sénégal. La place des PvVIH dans la dispensation communautaire est capitale, surtout en période de pandémie, car elles sont parvenues à démontrer leur capacité de résilience et d’adaptation.

# Research article: 210 - Expérience du district sanitaire de Kolda dans la mise en œuvre du conseil et dépistage du VIH à l’initiative du prestataire (CDIP)

**Published:** 2024-02-23

**Authors:** Khardiata BÂ, Thierno Cherif SY, Souleymane SAGNA, Mahamady SOUANÉ, Ibrahima SARR, Adama DIALLO, Fatou SAMA, Ousmane DIENG, Cheikh Oumar GUEYE, Aminata THIAM, Selly BÂ, Jules Bashi BAGENDABANGA

**Affiliations:** 1 District sanitaire de Kolda, Ministère de la santé et de l’action sociale (MSAS), Sénégal; 2 EpiC USAID/FHI 360, Sénégal

## Description du problème

En dehors du recours tardif aux soins noté dans nos contrées, beaucoup d’occasions manquées de dépistage ont été décrites dans la littérature avec un poids important du diagnostic tardif. Connaissant les bénéfices d’un traitement antirétroviral institué tôt, la mise en œuvre de stratégies innovantes et ciblées de dépistage permettrait d’améliorer la connaissance du statut et le recours précoce aux soins et par conséquent la mise en route d’un traitement.

## Activités menées et résultats obtenus

Durant la période allant d’octobre 2021 à juillet 2022, l’équipe du district sanitaire de Kolda avec l’appui technique et financier du projet EpiC (atteindre les cibles et maintenir le contrôle de l’épidémie) a rendu systématique le conseil et dépistage du VIH à l’initiative du prestataire (CDIP).

Le CDIP respecte les mêmes exigences et étapes que celles décrites dans le conseil de base : une séance d’information de groupe dans les salles d’attente suivie d’une autre de conseil individuel avec possibilité d’optout « consentement présumé » et d’opt-in « consentement explicite ».

Les populations suivantes, à l’exception des femmes enceintes, ont été ciblées à savoir : les enfants malnutris et leurs parents; les patients présentant une tuberculose, une hépatite virale, une IST, ou tout signe évocateur de l’infection à VIH, un accident d’exposition au sang (AES); toute autre personne exposée au risque d’infection VIH (partenaires de personnes vivant avec le VIH, populations clés, multi-partenariat sexuel, clients de professionnelles du sexe, jeunes, routiers…); les couples et futurs mariés et les patients hospitalisés.

Elles ont bénéficié d’une proposition systématique de dépistage. L’équipe a également envisagé le dépistage du partenaire. Selon le résultat du test, un soutien au maintien ou à l’adoption de comportements à moindre risque a été offert en cas de résultat négatif et une mise sous ARV précoce avec démarrage de la prophylaxie à l’INH en cas de séropositivité.

Ainsi, sur 1108 personnes prioritaires éligibles, 980 ont reçu la proposition du test dont 569 femmes/411 hommes soit un sexratio de 1,4 témoignant de la prédominance féminine. L’acceptation était de 99,5 % (975/980) avec une séropositivité de 37,2 % et un sex-ratio (F/H) de 2,7. La mise sous traitement ARV a été effective chez tous les cas positifs et dans les 24 heures suivant la confirmation. La prophylaxie à l’isoniazide a été initiée chez 87 % d’entre eux.

Toutefois, plus de la moitié des clients dépistés (69 %) étaient aux stades 3 et 4, témoignant d’un diagnostic tardif dans cette zone mais également du profil de patients présentant des signes évocateurs d’infection à VIH.

## Leçons apprises et étapes futures

Le CDIP est efficace et peu coûteux, cependant les clients sont le plus souvent dépistés en stade avancé d’où la nécessité de l’associer à d’autres stratégies telles que l’index testing.

# Research article: 51 - Acceptabilité et faisabilité de la thérapie interpersonnelle dans la prise en charge de la dépression chez des personnes vivant avec le VIH au Sénégal

**Published:** 2024-02-23

**Authors:** Charlotte BERNARD, Salaheddine ZIADEH, Judicaël Malick TINE, Abibatou DIAW, Ibrahima NDIAYE, Oumar SAMBA, Thierry BOTTAI, Laurent JACQUESY, Ndeye Fatou NGOM, François DABIS, Nathalie De REKENEIRE, Moussa SEYDI

**Affiliations:** 1 Université de Bordeaux, Institut national de la santé et de la recherche médicale (INSERM), Institut de recherche pour le développement (IRD), Bordeaux Population Health Research Centre, UMR 1219, Team GHiGS, Bordeaux, France; 2 Global Mental Health Lab, Teachers College, Columbia University, New York, États-Unis; 3 Université libanaise, Faculté de santé publique, Sidon, Liban; 4 Service des maladies infectieuses et tropicales, Centre hospitalier national universitaire (CHNU) de Fann, Dakar, Sénégal; 5 Centre de traitement ambulatoire, CHNU de Fann, Dakar, Sénégal; 6 Service de psychiatrie, CHNU de Fann, Dakar, Sénégal; 7 Pôle de psychiatrie, Centre hospitalier de Martigues, France; 8 Psychiatre indépendant, président de CREATIP, Annecy, France; 9 Fondation Mérieux, Lyon, France

## Introduction

La dépression est un trouble mental fréquent chez les personnes vivant avec le VIH (PvVIH), ayant des conséquences non négligeables sur leur prise en charge. Dans les pays à ressources limitées, l’Organisation mondiale de la santé (OMS) recommande la thérapie interpersonnelle de groupe (TIP-G) pour la prise en charge de la dépression. La faisabilité et l’acceptabilité de la TIP-G ont été évaluées au Sénégal chez les PvVIH présentant une dépression.

## Méthodes

Un dépistage de la dépression a été effectué par le Patient Health Questionnaire (PHQ-9) chez des PvVIH suivies dans le service des maladies infectieuses et tropicales et le centre de traitement ambulatoire du Centre hospitalier national universitaire (CHNU) de Fann à Dakar. Les patients avec un PHQ-9 ≥ 5 rencontraient un psychiatre pour confirmer le diagnostic de dépression. Après confirmation et conformément au manuel de l’OMS sur la mise en place de la TIP-G, chaque patient bénéficiait d’une séance individuelle puis de 8 séances de groupe hebdomadaires. Les groupes de TIP-G étaient menés par un assistant social ou un médiateur formé. Les taux de refus, d’abandon, d’absence et la satisfaction globale vis-à-vis de la thérapie ont été évalués, ainsi que l’évolution de la sévérité des symptômes dépressifs.

## Résultats

Le diagnostic de dépression a été confirmé pour 75 des patients interrogés. Cinq patients (6 %) ont refusé de participer à la TIP-G. Parmi les 70 ayant participé, 13 % ont abandonné après au moins 3 séances de thérapie, le plus souvent pour des raisons indépendantes de leur volonté (56 %) (hospitalisation, déménagement, inondation, problème de garde d’enfants). La majorité des patients ayant complété la TIP-G (97 %) ont assisté à 7 séances ou plus et les absences étaient justifiées (décès dans la famille, maladie, contrainte de travail). Les patients ayant fait la visite de fin d’intervention rapportent tous un niveau de satisfaction globale élevé vis-à-vis de la TIP-G. Avant le début de la thérapie, 23 % des patients présentaient des symptômes dépressifs légers, 49 % des symptômes modérés, 27 % des symptômes modérément sévères à sévères. En fin de la thérapie, seuls 22 % avaient des symptômes dépressifs, de sévérité légère à modérée (20 % et 2 %, respectivement). Entre le début et la fin de la thérapie, l’amélioration des symptômes dépressifs était significative (83 % ± 23 %, t = 19 – séries appariées, p

## Conclusion

L’utilisation de la TIP-G au Sénégal est à notre connaissance une première en Afrique de l’Ouest. Cette thérapie semble bien acceptée, faisable et efficace pour traiter la dépression chez les PvVIH sénégalaises. Le format de groupe est un atout pour briser l’isolement, permettre les échanges entre pairs et offrir un accès plus aisé au traitement de la dépression dans les centres de prise en charge du VIH. L’évaluation 3 mois après la fin de la thérapie permettra d’observer les effets à plus long terme de la thérapie.

# Research article: 265 - Évaluation de la maturité du traitement différencié au Sénégal

**Published:** 2024-02-23

**Authors:** Kouro BOUSSO, Papa Oumar DIAGNE, Aminata THIAM, Karim DIOP, Fatou FALL, Bintou SYLLA, Khadija FALL, Ndeye Binta NDIAYE, Alassane Moussa NIANG, Cheikh Tidiane NDOUR

**Affiliations:** 1 Division de lutte contre le sida et les IST (DLSI), Ministère de la santé, Sénégal; 2 EpiC USAID/FHI 360, Sénégal

## Contexte

En septembre 2020, le Sénégal a intégré le réseau CQUIN (Coverage, Quality and Impact Network) qui est un réseau d’apprentissage regroupant 21 pays d’Afrique, conçu pour accélérer la mise à l’échelle de la prestation de services différenciés à travers l’appui technique, les communautés de pratique, les visites de pays à pays ainsi que les réunions thématiques annuelles. En septembre 2022, le Sénégal a adopté et renseigné le nouveau tableau de bord 2.0 sur le traitement différencié utilisé par l’ensemble des pays du réseau.

## Objectif

Il s’agit d’une auto-évaluation qui a pour objectif de mesurer les progrès accomplis en matière de traitement différencié, et d’identifier les lacunes à des fins d’élaboration d’un plan d’action.

## Méthodologie

Il s’agissait d’une activité participative et inclusive avec l’implication des parties prenantes à savoir le Ministère de la santé (Division de lutte contre le sida et les IST, Direction de la Qualité de la sécurité et de l’hygiène hospitalière, Pharmacie nationale d’approvisionnement), le Conseil national de lutte contre le sida, les bénéficiaires – Réseau national des Associations de personnes vivant avec le VIH/SIDA, Réseau national des populations clés, ABOYA (Association nationale de femmes vivant avec le VIH au Sénégal) -, le Centre de traitement ambulatoire ainsi que les partenaires techniques et financiers. 18 domaines ont été évalués avec une échelle de maturité allant du rouge au vert foncé, à savoir de l’étape préliminaire à l’étape d’implémentation très évoluée.

## Résultats

Au total sur les 18 domaines, seuls 4 ont atteint un niveau de maturité maximale. Ce sont les politiques, les orientations opérationnelles, la coordination, la couverture de traitement différencié dans les établissements. Par contre, des efforts doivent être fournis sur les plans de la mise à l’échelle, du suivi de l’évaluation, de la tuberculose/VIH, des services de santé maternelle et infantile différenciés, de l’intégration des services de planning familial dans les modèles de traitements antirétroviraux (TAR) différenciés, de la qualité et de l’impact des services de TAR différencié.

## Conclusion

L’auto-évaluation a permis au pays de disposer de données de base. Le défi réside dans le virage du rouge au vert foncé et le maintien des acquis.

# Research article: 231 - Prévalence et facteurs associés à la positivité de la sérologie VIH parmi les populations clés de la plateforme SENCAS au Sénégal

**Published:** 2024-02-23

**Authors:** Kouro BOUSSO

**Affiliations:** Division de lutte contre le sida et les IST (DLSI), Ministère de la santé, Sénégal

## Contexte

Les populations les plus vulnérables sont touchées de manière disproportionnée par le VIH à l’échelle mondiale et au Sénégal. Cette étude analyse la prévalence du VIH et les facteurs associés dans les populations clés inscrites au niveau de la plateforme de notification unique appelée SENCAS entre 2012 et 2019 en collaboration avec le CDC (Center for Disease Control and Prevention).

## Méthodes

Nous avons réalisé une étude transversale avec une population composée de 13 475 sujets dont 8384 hommes ayant des relations sexuelles avec d’autres hommes (HSH), 5083 professionnelles du sexe (PS) et 8 consommateurs de drogues injectables (CDI) ayant tous bénéficié d’un dépistage du VIH.

Les variables relatives aux circonstances de contamination et aux données sociodémographiques ont été analysées. Les prévalences et les rapports de cotes ont été calculés avec des intervalles de confiance à 95 %, et un modèle de régression logistique a été réalisé pour identifier les facteurs associés à la positivité de la sérologie VIH à l’aide du logiciel Stata 16.

## Résultats

Globalement de 26 ans (22-31), l’âge médian était de 29 ans, 25 ans et 38,5 ans respectivement chez les PS, les HSH et les CDI. La prévalence du VIH était de 4,58 % chez les PS, 12,50 % chez les CDI et 11,61 % dans le groupe des HSH.

Le modèle explicatif a montré une prévalence plus élevée chez les HSH (OR = 3,14; IC 95% : 2.5- 3,7); les populations clés appartenant à la catégorie d’âge inférieur à 33 ans (OR = 1,19; IC 95% : 1,02-1,38); celles qui n’étaient pas de nationalité sénégalaise (OR = 2,2; IC 95% : 1.6- 3); celles en union au moment de l’enquête (OR = 1,16; IC 95% : 1,3-2); les veufs (OR = 5,8; IC 95% : 3,7-9); celles n’ayant reçu qu’une éducation en arabe (OR = 1,16; IC 95% : 1,18- 2), le niveau d’étude secondaire étant un facteur protecteur; celles qui exerçaient un travail (OR = 1,26; IC 95% : 1-1,5); et celles qui avaient un enfant ou plus (OR = 2; IC 95% : 1,7-2,3). Au plan géographique, les prévalences étaient significativement plus élevées au niveau des régions de Dakar (OR = 2; IC 95% : 1,6-2,6), Thiès (OR = 2,3; IC 95% : 1,7-3), Kaolack (OR = 2,3; IC 95% : 1.6- 3,2), Saint-Louis (OR = 2,6; IC 95% : 1,8-3,8) et Sédhiou (OR = 1,6; IC 95% : 1,1-2,5).

## Conclusion

Une prévalence élevée de l’infection a été constatée dans les groupes clés, confirmant le caractère concentré de l’épidémie au Sénégal. Ce constat justifie amplement la mise en œuvre de stratégies innovantes notamment de prévention avec la prophylaxie pré-exposition (PrEP) ciblant ces populations hautement vulnérables au premier rang desquelles les jeunes HSH.

# Research article: 67 - Dépistage des dysplasies cervicales et des infections à papiNomavirus humain (PVH) par les sages-femmes : expérience du programme « Casamance Research program on HIV-Resistance and Sexual Health » CARES à Ziguinchor, Sénégal

**Published:** 2024-02-23

**Authors:** Sally CAMARA, Noël Magloire MANGA, Assane NDOYE, Benjamin Amaye SAMBOU, Mame Aissé THIOUBOU, Vic ARENDT, Henri GOEDERTZ, Boubacar DIOUF

**Affiliations:** 1 ENDA Santé; 2 Université Assane Seck, Ziguinchor, Sénégal; 3 Centre hospitalier régional de Ziguinchor, Sénégal

## Objectif

Le dépistage et la prise en charge des dysplasies cervicales PVH induites constituent la principale stratégie de lutte contre le cancer du col chez l’adulte au Sénégal. L’implication des sages-femmes, maillon important du système de santé et premier contact de la majorité des femmes en âge de reproduction dans la mise en œuvre de cette stratégie, est un véritable challenge. L’objectif de notre travail est de partager l’expérience d’une stratégie de dépistage par des sagesfemmes des dysplasies cervicales et de l’infection à PVH par trois méthodes au niveau des structures de santé surtout périphériques.

## Méthodologie

Il s’agit d’une étude prospective multicentrique réalisée au niveau de 17 postes de santé, 2 centres de santé et 2 hôpitaux de la région médicale de Ziguinchor. Les sages-femmes de ces structures ont été formées à la réalisation du dépistage par inspection visuelle (IVA/IVL), à la réalisation de frottis cervico-utérins (FCU) et de prélèvements pour la recherche de PVH par GeneXpert. Les femmes consentantes appartenant à la population générale ou à des populations clés ont été dépistées. Une prise en charge par cryothérapie ou thermothérapie et conisation du col (LEEP) a été réalisée en fonction du type de lésions.

## Résultats

Entre octobre 2019 et juin 2022, 6356 femmes de la population générale, 945 femmes vivant avec le VIH et 288 professionnelles du sexe ont été dépistées par la technique IVA/IVL. La proportion de cols suspects était respectivement de 4,7 %, 2,2 % et 4,5 % pour ces différentes populations.

La recherche de PVH au GeneXpert a été réalisée chez 1282 femmes de la population générale, 603 femmes vivant avec le VIH et 132 professionnelles du sexe. La proportion de cas positifs était respectivement de 6 %, 21 % et 0 % pour ces différentes populations. Un FCU a été réalisé chez 4927 femmes de la population générale, 909 femmes vivant avec le VIH et 216 professionnelles du sexe.

Après confirmation par les gynécologues, 4 patientes ont bénéficié d’une cryothérapie, 42 d’une thermothérapie et 21 d’une LEEP (conisation du col) avec l’appui des gynécologues. Un cas d’hystérectomie a été réalisé sur cancer avancé. 7 néoplasies avancées et 1 tumeur bourgeonnante et saignante du col ont été référées.

## Conclusion

Cette étude de mise en œuvre démontre l’intérêt et la faisabilité de l’implication des sages-femmes dans le dépistage des dysplasies cervicales PVH induites en zone décentralisée. La prévalence de l’infection à PVH est élevée chez les femmes vivant avec le VIH et dans la population générale.

# Research article: 129 - Aptitude à la transition dans les soins chez les adolescents et jeunes adultes vivant avec le VIH en Afrique de l’Ouest

**Published:** 2024-02-23

**Authors:** Fatou Kiné NDIAYE, Aminata DIACK, Mariama KANE, Cécile CAMES, Gérès AHOGNON, Arthur Christian SAWADOGO, Caroline YONABA, Sylvie OUÉDRAOGO, Tatiana OUÉDRAOGO, Jocelyne KYELEM, Fatimata BARRY, Hermann KABORE

**Affiliations:** 1 Réseau Enfants et VIH en Afrique, Dakar, Sénégal; 2 REGIPIV-BF (Réseau national pour une plus grande implication des personnes infectées et affectées par le VIH/sida au Burkina Faso), Ouagadougou, Burkina Faso; 3 Centre hospitalier universitaire Yalgado Ouédraogo, Ouagadougou, Burkina Faso; 4 Centre hospitalier universitaire Charles de Gaulle, Ouagadougou, Burkina Faso; 5 Centre hospitalier Roi Baudouin, Guédiawaye, Sénégal; 6 Centre hospitalier de Pissy, Ouagadougou, Burkina Faso; 7 Centre hospitalier de Kossodo, Ouagadougou, Burkina Faso; 8 Centre hospitalier national d’enfants Albert Royer, Dakar, Sénégal; 9 UMI233 TransVIHMI, UR1175, Institut de recherche pour le développement (IRD), Dakar, Sénégal

## Objectifs

Pour les adolescents et jeunes adultes vivant avec le VIH (AJAvVIH), le transfert des services de pédiatrie vers la médecine adulte comporte des risques de baisse d’observance au traitement antirétroviral (TAR), de rupture de soins et d’augmentation de la morbi-mortalité. Un processus accompagné et coordonné de transition (PACTE) dans les soins a été mis en place dans le cadre du programme TRANSITIONS au Sénégal et au Burkina Faso pour promouvoir la santé et le bien-être des patients. L’objectif de cette analyse est d’évaluer le niveau d’aptitude des jeunes à l’entrée dans le PACTE et d’explorer les facteurs associés.

## Matériels et méthodes

Le PACTE inclut les AJAvVIH de 15 à 24 ans informés de leur statut VIH, sous TAR et suivis dans 4 sites hospitaliers pédiatriques au Burkina Faso et 2 au Sénégal. Il comporte 3 phases : préparation en pédiatrie (± 6 mois), transition en service de médecine adulte et engagement dans les soins adultes. La préparation consiste à informer les patients sur le PACTE, présenter et collecter le choix du site adulte et renforcer l’éducation thérapeutique, en privilégiant les séances de groupe. À l’issue de la 1^re^ phase, l’aptitude à la transition est mesurée par 18 questions dans 3 dimensions : autonomie, connaissances et compétences du patient dans sa prise en charge du VIH. Un score moyen allant de 0 (moins bonne aptitude à la transition) à 100 (meilleure aptitude à la transition) est calculé. Les facteurs psycho-sociodémographiques associés à la valeur du score ont été explorés à l’aide d’un modèle de régression linéaire généralisée. Le modèle a été ajusté pour le sexe, le pays et l’âge à l’annonce du statut VIH.

## Résultats

Entre avril et septembre 2021, 194 AJAvVIH ont intégré la phase préparatoire du PACTE, dont : 48 % sont des filles, 54 % ont moins de 19 ans, 67 % sont suivis au Burkina Faso, 53 % vivent avec leur mère, 92 % ont été scolarisés, 54 % ont connu leur statut VIH avant 15 ans et 44 % sont actifs sexuellement. Le score moyen d’aptitude à la transition était 71 (écart-type = 14).

Les facteurs associés à un score d’aptitude plus bas étaient d’avoir moins de 19 ans (-8,7 ± 1,9, p < 0,0001), ne pas avoir dépassé le niveau primaire à l’école (-8,4 ± 2,2, p = 0,0002), ne pas avoir partagé son statut VIH avec l’entourage (-3,8 ± 1,8, p = 0,04), ne pas souhaiter le faire (-3,9 ± 2,2, p = 0,07) et être orphelin (-4,5 ± 2,7, p = 0,09).

## Conclusion

Le niveau d’aptitude à la transition dans les soins était satisfaisant dans cette population, malgré les restrictions sanitaires qui ont impacté la phase de préparation dans les deux pays. Cependant, une attention particulière au cours du PACTE doit être portée aux plus jeunes et aux plus vulnérables socialement. Enfin, le PACTE devrait être une opportunité de soutenir les jeunes qui souffrent d’isolement et d’autostigmatisation, dans le partage de sérologie avec leur entourage.

# Research article: 122 - Un dispositif d’annonce standardisée de la sérologie VIH permet le maintien de la suppression virologique à 24 mois chez les adolescents suivis au Centre hospitalier national d’enfants Albert Royer, Dakar

**Published:** 2024-02-23

**Authors:** >Ndèye Fatou DIALLO, Aminata DIACK, Fatou Kiné NDIAYE, Khady SIDIBÉ, Astou Diop DIÈYE, Fatou NDIAYE, Mohamed Lamine Ibrahima SOUANÉ, Cécile CAMES

**Affiliations:** 1 Centre hospitalier national d’enfants Albert Royer (CHNEAR), Dakar, Sénégal; 2 TransVIHMI, Université de Montpellier, INSERM, IRD, Montpellier, France

## Objectifs

Depuis 2011, l’OMS préconise que le processus d’annonce du statut sérologique des enfants d’âge scolaire soit achevé avant l’âge de 12 ans. Une annonce tardive ou mal conduite est associée à un risque de baisse de l’observance aux antirétroviraux (ARV), d’échec thérapeutique et de désengagement des soins. Pourtant, le taux d’annonce dans les files actives en Afrique de l’Ouest reste très faible. Outre la réticence des parents, le manque de formation des soignants et l’absence de protocole d’annonce constituent des facteurs institutionnels déterminants. La présente analyse rétrospective décrit l’impact d’un dispositif d’annonce standardisée (DAS) de leur sérologie VIH aux enfants et adolescents sur la rétention dans les soins et le maintien ou l’atteinte de la suppression virologique (SV) à 24 mois.

## Matériels et méthodes

À partir de janvier 2015, un DAS a été mis en place dans le suivi de routine de la cohorte du CHNEAR, articulant une phase « pré-annonce », l’annonce formelle « le jour J », et le suivi « post-annonce » à court et moyen terme. Pour être éligible au DAS, les enfants infectés par le VIH-1 devaient être âgés d’au moins 7 ans, ne pas présenter de troubles mentaux, avoir un répondant stable. L’analyse a inclus ceux sous ARV depuis au moins 1 an avant d’intégrer le dispositif d’annonce avec une mesure de charge virale dans les 12 mois précédant la date d’annonce et au moins une mesure dans les 24 mois suivants. Les facteurs associés à l’échec virologique à 24 mois post-annonce ont été identifiés par un modèle de régression logistique pas à pas. La SV a été définie comme une charge virale < 1000 copies/ml de sang.

## Résultats

Parmi 172 enfants bénéficiaires du DAS, 119 ont été inclus dans l’analyse : 45 % de filles, âge médian à l’annonce 12,6 ans (IQR = 11,8-13,8) et 70 % en 1^re^ ligne. À 24 mois post-annonce, 116 étaient toujours suivis, 2 ont été transférés en service adulte et 1 enfant était perdu de vue.

En pré-annonce, 79 % des participants étaient en SV. En post-annonce, la SV était maintenue à 78 % au cours de la 1^re^ année (test apparié de MacNemar non significatif) et à 81 % au cours de la 2^e^ année (NS).

Être en 2^e^ ligne d’ARV (aOR = 3,0; IC 95% : 1,1-8,5), présenter une charge virale détectable (3,2; 1,0-9,7) et souffrir de malnutrition chronique (3,0; 1,1-8,3), en pré-annonce, étaient associés à un échec virologique en post-annonce.

## Conclusion

Le DAS a permis de réaliser l’annonce auprès d’un grand nombre d’enfants et d’adolescents tout en maintenant les acquis en matière de SV et d’engagement dans les soins dans la cohorte du CHNEAR. Ces résultats plaident pour la systématisation de l’annonce standardisée et précoce aux enfants et adolescents. La charge virale annuelle est un outil majeur de prise en charge en post-annonce et devrait être garantie par le programme national.

# Research article: 203 - Évaluation des modifications du poids corporel après passage des inhibiteurs non nucléotidiques de la transcriptase inverse (INNTI) au dolutégravir (DTG) : étude de cohorte de personnes vivant avec le VIH suivies au CRCF/CHNU Fann, Dakar, Sénégal

**Published:** 2024-02-23

**Authors:** Ndèye Amy NDIAYE, Aicha DIALLO, Khady HANN, Aminata NIANG, Séverine CARILLON

**Affiliations:** 1 Centre régional de recherche et de formation à la prise en charge clinique de Fann (CRCF), Dakar, Sénégal; 2 Centre hospitalier national universitaire de Fann (CHNU Fann), Dakar, Sénégal

## Contexte

Depuis 2019 a été recommandée au Sénégal une trithérapie antirétrovirale à base de dolutégravir en alertant sur une éventuelle prise de poids lors de son utilisation. Or, la prise de poids et les modifications du poids corporel déjà constatées dans le traitement du VIH avec les régimes à base d’INNTI restent problématique car pouvant entraîner de graves comorbidités (telles que HTA, diabète) dont la prise en charge n’est pas à ce jour intégrée dans l’offre globale des soins VIH.

## Objectif

Explorer si la prise de poids sous dolutégravir était associée à un retour à un état général satisfaisant ou à une obésité pouvant entraîner ou aggraver un facteur de risque cardiovasculaire chez des patients exposés aux INNTI.

## Méthode

À partir de la base de données relatives au traitement antirétroviral de la pharmacie du CRCF ont été sélectionnés tous les patients régulièrement suivis âgés de plus de 18 ans ayant été exposés aux INNTI et recevant du dolutégravir (DTG) depuis au moins 6 mois durant la période de septembre 2019 à juin 2022. Puis nous avons procédé à une analyse multivariée entre les facteurs démographiques, le régime antirétroviral et les modifications du poids corporel.

## Résultats

301 patients étaient régulièrement suivis sous DTG, et avaient été exposés à l’éfavirenz (EFV) 159 femmes et 94 hommes, ou à la névirapine (NVP) 32 femmes et 16 hommes. Les hommes étaient plus fréquemment âgés de 25 à 49 ans (90/110) et les femmes de plus de 50 ans (88/191).

- La prise de poids survenait chez 116/191 des femmes entre 1-38 kg avec une durée moyenne du traitement sous DTG de 14 mois (6-21) mois, et chez 68/110 des hommes entre 1-21 kg avec une durée moyenne du traitement sous DTG de 27 mois (6-47) mois.
- Chez les femmes exposées à l’EFV (103/116) la prise de poids était le plus souvent entre 0-5 kg (53/103), de 5-10 kg (38/103) et + 10 kg (12/103). Parmi celles exposées à la NVP (13/116) : 0-5 kg (6/13), 5-10 kg (3/13) et + 10 kg (3/13).
- Chez les hommes exposés à l’EFV (56/68) la prise de poids était entre 0-5 kg (32/56), de 5-10 kg (18/56) et + 10 kg (6/56). Parmi ceux exposés à la NVP (12/68) : 0-5 kg (7/12), de 5-10 kg (5/12) et + 10 kg (0/12).
- Une perte de poids avait été relevée chez 28/159 des femmes et 21/94 des hommes exposés à l’EFV, et 12/32 des femmes, 4/16 des hommes exposés à la NVP.
- Une obésité sous DTG était confirmée chez 100/301 des patients avec un IMC > 25, dont 76 femmes (58 exposées à l’EFV, 18 à la NVP) et 24 hommes (21 exposés à l’EFV, 3 à la NVP).
- Une prise de poids sous DTG chez des patients expérimentés aux INNTI n’était pas systématique, était plus fréquente chez les femmes ayant déjà été exposées à l’EFV à 14 mois et, était rarement associée à une obésité dans moins de 1 % des cas (0,65).

## Conclusion

Il est nécessaire d’accorder une attention particulière à l’évolution du poids chez les femmes âgées diagnostiquées d’une ou de plusieurs comorbidités.

# Research article: 85 - Expérience du district sanitaire d’Oussouye dans la mise en œuvre du marrainage des femmes enceintes séropositives

**Published:** 2024-02-23

**Authors:** Omar CISSÉ, Diombraise Gabriel DIATTA

**Affiliations:** District sanitaire d’Oussouye, Initiative sénégalaise d’accès aux antirétroviraux (ISAARV) Ziguinchor

## Introduction

En marche vers l’élimination de la transmission mère-enfant du VIH, le Sénégal a mis en œuvre depuis 2018 une stratégie de marrainage de femmes enceintes séropositives et de leurs enfants, dénommée « Ndeye-Dické ».

L’accès aux services de prévention de la transmission mère-enfant (PTME) a fait l’objet d’une extension importante jusqu’au niveau des postes de santé, offrant ainsi des services de PTME à travers la délégation des tâches et l’intégration des services, autorisant le personnel paramédical à prendre en charge le couple mère-enfant dans le cadre de l’élimination de la transmission mère-enfant (eTME).

En 2021, la région de Ziguinchor était à 51 % de mise sous traitement antirétroviral (TARV) des femmes enceintes séropositives, parmi lesquelles 17 % ont bénéficié d’une mesure de la charge virale, tandis que 29 % des enfants nés de mères séropositives avaient bénéficié de PCR dans les délais.

C’est dans ce contexte que nous avons réalisé cette étude dont l’objectif est d’évaluer l’impact de cette stratégie dans le suivi du couple mère-enfant dans le district sanitaire d’Oussouye.

## Méthodologie

Il s’est agi d’une étude rétrospective descriptive portant sur les femmes enceintes séropositives et leur enfant durant l’année 2021.

Les rendez-vous étaient programmés afin d’assurer la disponibilité du paquet de soins (intégration des services). Le suivi se faisait avec les structures de référence lorsque les femmes accouchaient dans les hôpitaux de référence.

Le suivi des femmes enceintes était assuré par les sages-femmes qui transmettaient les informations au point focal PTME. La relance se faisait lors des réunions de coordination par le partage de la maquette PTME et le recueil des difficultés.

## Résultats

Durant l’année 2021, toutes les femmes enceintes reçues au premier contact en consultation prénatale ont bénéficié du dépistage du VIH (100 %), celles séropositives (16) ont toutes été marrainées par une sagefemme avec 100 % de mise sous TARV.

Au cours du suivi, 77 % (7/9) des femmes enceintes séropositives éligibles ont bénéficié d’une mesure de la charge virale, et tous les résultats reçus étaient indétectables (2).

Tous les nouveau-nés de mères séropositives ont bénéficié d’une prophylaxie à la naissance (7), de même que les nourrissons (8) qui ont tous bénéficié d’une PCR dans les délais (100 %), sans aucun résultat positif reçu (2). Concernant la sérologie définitive, 8 sur les 9 suivis en ont bénéficié.

## Conclusion

La délégation des tâches bien conduite dans un contexte d’intégration des services dans le district d’Oussouye a permis d’enregistrer des résultats encourageants. Il devient alors opportun de documenter les bonnes pratiques et de les partager pour une mise à l’échelle au niveau régional, voire national, pour l’atteinte des objectifs d’élimination de la transmission mère-enfant du VIH.

# Research article: 68 - Impact de la décentralisation du diagnostic et du suivi virologique du VIH au Sénégal : cas d’étude au niveau de la région de Louga

**Published:** 2024-02-23

**Authors:** Khady Diatou COULIBALY, Bousso NIANG, Ndeye Marame DIONE, Brianan KIERNAN, Ndeye Fatou NGOM, Karim DIOP, Halimatou DIOP-NDIAYE, Coumba TOURÉ KANE, Cheikh Tidiane NDOUR

**Affiliations:** 1 Division de lutte contre le sida et les IST (DLSI), Ministère de la santé, Sénégal; 2 Laboratoire de bactériologie et de virologie, Centre hospitalier national universitaire Aristide Le Dantec, Dakar, Sénégal; 3 Hôpital Dalal Jamm/IRESSEF (Institut de recherche en santé, de surveillance épidémiologique et de formation), Sénégal; 4 UNICEF; 5 Clinton Health Access Initiative (CHAI)

## Contexte

En 2017, au Sénégal, la mesure de la Charge virale (CV) et le Diagnostic précoce (DP) du VIH étaient limités aux tests de laboratoires centralisés d’où un long délai de transport, de rendu des résultats et de prise en charge des patients. Avec une file active de 1122 PvVIH sous ARV, la région de Louga située à 200 km de la capitale, ne disposait pas de plateforme conventionnelle malgré ses 7 centres de santé et ses 2 hôpitaux. En 2018, le Sénégal s’est donc engagé dans la décentralisation du DP et de la CV à travers le projet « Point-of-Care » (POC) qui consistait à optimiser les GeneXpert pour le VIH-1.

## Objectifs

Cette étude visait à évaluer les performances de la région de Louga en matière de DP des enfants et de mesure de la CV du VIH-1.

## Méthodologie

Des études rétrospectives et prospectives ont été réalisées. La population d’étude était constituée de nourrissons nés de mères séropositives dits enfants exposés au VIH à partir de 6 semaines de vie et de personnes vivant avec le VIH ayant besoin d’un test CV pour le suivi au 6^e^ mois de mise sous ARV ou lors de leur suivi annuel, de la région de Louga. Les données ont été obtenues à partir des rapports de taux de survie et des revues CV et DP. Une collecte et une analyse des données de la CV et du DP ont été faites pour les périodes de janvier à décembre de 2017 à 2021.

## Résultats

Les résultats de cette étude ont montré un accès faible aux tests de CV et DP entre 2017 et 2018, avec un taux de réalisation des tests de 0 % (CV) et 2 % (DP). L’optimisation du GeneXpert avec le projet POC a permis d’obtenir une évolution de la mesure de la CV et du DP de 52,3 % et 50 % en 2020, et 60,6 % et 75 % en 2021. Le taux de réalisation des tests était de 94,6 % pour la CV et de 100 % pour le DP. Les résultats étaient rendus en moins de 24 h. Au total 86,5 % ont eu une suppression virale, dont 72,22 % des enfants de moins de 15 ans. Le taux de positivité du DP était de 0 %. Parmi les patients présentant une CV > 1000 cp/ml (13,5 %) le délai médian pour obtenir un conseil sur l’adhérence a été réduit de 1 à 3 jours.

## Conclusion

La décentralisation de la mesure de la CV et du DP a permis, au niveau de la région de Louga, l’amélioration des taux de réalisation des tests du VIH et du temps de rendu des résultats, permettant ainsi une meilleure prise en charge clinique des patients. Cependant cette performance de 2021 avait été limitée, en cette période de pandémie de Covid-19, par des ruptures en cartouches de CV et DP, une faible demande en CV et DP, des pannes de modules du POC et un nombre élevé de patients sans résultats.

# Research article: 174 - Statut immunologique des gestantes vis-à-vis du VHB et effet protecteur de la vaccination chez les nourrissons de mères positives au VHB

**Published:** 2024-02-23

**Authors:** Sahal DARAR DIRIR, Ambroise AHOUDI, Mohamed Houmed ABOUBAKAR, Halimatou DIOPNDIAYE

**Affiliations:** 1 Laboratoire de bactériologie et de virologie (LBV), Centre hospitalier national universitaire Aristide Le Dantec, Dakar, Sénégal; 2 Institut de recherche en santé, de surveillance épidémiologique et de formation (IRESSEF), Sénégal

## Objectifs

L’objectif de cette étude était de déterminer le statut immunologique des gestantes vis-à-vis du VHB et d’évaluer l’effet protecteur de la vaccination chez les nourrissons de mères positives au VHB.

Les objectifs spécifiques étaient :

- déterminer le statut immunologique des gestantes;
- déterminer la prévalence de la transmission mère-enfant du VHB;
- évaluer la réponse vaccinale des nourrissons de mères positives au VHB.

## Méthode

Une étude prospective d’octobre 2021 à février 2022 a été menée dans 9 centres de consultation prénatale de la ville de Djibouti. Les femmes enceintes porteuses de l’AgHBs et leurs nouveau-nés ont été recrutés dans l’étude. Les femmes étaient testées pour les différents marqueurs du VHB (AgHBe, Ac anti-HBs et anti-HBc) avec l’automate VIDAS.

Pour les nourrissons nés de mères positives, des injections initiales d’HBIG (immunoglobuline) et du vaccin contre l’hépatite ont été effectuées dans les heures suivant la naissance. Les taux d’infection par le VHB chez les nourrissons ont été évalués à l’âge de 7 mois au laboratoire de CSl pour la recherche de l’AgHBs et des Ac anti-HBs. La réponse vaccinale était jugée selon le taux d’Ac anti-HBs obtenu après la troisième dose, l’absence de réponse (taux 100 mUI/ml).

## Résultats

Sur 880 femmes enceintes, 88 étaient porteuses de l’AgHBs soit un taux de 9,3 %. Seules, 56 ont accepté de poursuivre l’enquête. L’âge moyen était de 32 ans; les gestantes positives étaient grandes multipares (n = 13; 25 %), non éduquées à 44 % (n = 24) et sans emploi dans 60 % (n = 33) des cas. Aucune n’était vaccinée contre le VHB. La charge virale était disponible pour 56 gestantes et 1 % d’entre elles présentaient une charge virale supérieure à 50 000 copies/ml et un taux d’AgHBe détectable. La totalité des gestantes présentaient des Ac anti-HBc décrivant une infection ancienne.

Un total de 33 nourrissons âgés de 7 mois ont pu être dépistés parmi lesquels 36 % sont de sexe masculin et 64 % de sexe féminin, avec un poids moyen de 2,5 kg (min 1,8 kg et max 3,1 kg). Seul 1/33 nourrisson né de mère positive à AgHbs était infecté par le VHB. Pour la réponse vaccinale, 5 % d’entre eux n’avaient pas de réponse vaccinale, 25 % présentaient une réponse vaccinale faible et 70 % présentaient une réponse vaccinale élevée.

## Conclusion

Au terme de cette étude, on peut dire que malgré une prévalence élevée, très peu de gestantes présentaient les facteurs prédictifs (charge virale élevée + AgHBe positif) de la transmission mère-enfant (TME). On remarque que l’HBIG plus le vaccin contre le VHB préviennent de manière efficace la TME du VHB.

# Research article: 40 - Séroprévalence et facteurs de risque associés à l’infection par le virus de l’hépatite B chez les étudiants de N’Djamena, au Tchad

**Published:** 2024-02-23

**Authors:** Nalda DEBSIKREO, Birwé Léon MANKRÉO, Azoukalné MOUKÉNET, Merwa OUANGKAKE, Nathan MARA, Nafissatou Leye DIOUF, Gora LO, Halimatou DlOP-NDIAYE, Ali Mahamat MOUSSA, Ndèye Coumba TOURÉ KANE, Françoise LUNEL-FABIANI

**Affiliations:** 1 Université Cheikh Anta Diop, Dakar, Sénégal; 2 Institut de recherche en santé, de surveillance épidémiologique et de formation (IRESSEF), Sénégal; 3 Université de N’Djaména, Tchad; 4 Hôpital de la Paix, N’Djaména, Tchad; 5 Centre hospitalier universitaire de référence, N’Djaména, Tchad; 6 Centre hospitalier universitaire d’Angers, France

## Objectif

L’hépatite virale est une menace majeure de santé publique. Au Tchad, la recherche du virus de l’hépatite B (VHB) n’est pas systématique. Ainsi, la plupart des personnes à risque telles que les étudiants ignorent leur statut. La plupart des personnes infectées de façon chronique par le VHB ne présentent aucun symptôme, ou très peu. Notre étude a cherché à déterminer la séroprévalence du VHB et à identifier les facteurs de risque en milieu estudiantin et jeune afin d’informer les politiques sur la nécessité d’implémenter des mesures visant à éliminer les hépatites virales à l’horizon 2030, comme recommandé par l’OMS.

## Méthodes

Nous avons mené une étude transversale du 3 au 23 juillet 2021 auprès des étudiants des universités de N’Djaména et d’Emi Koussi sélectionnés aléatoirement. Un questionnaire structuré a été utilisé pour recueillir des données sur les caractéristiques sociodémographiques et les facteurs de risque. La recherche de l’AgHBs avait été effectuée à l’aide du kit de test rapide de Determine HBsAg” (Alere Determine™ HBsAg, Waltham, MA, États-Unis). Des analyses descriptives et une modélisation par régressions logistiques ont été menées pour déterminer la relation entre les variables dépendantes et les facteurs sociodémographiques. L’Odds ratio (OR) et l’intervalle de confiance (IC) à 95 % ont été utilisés comme indicateurs de la force de l’association. Une p value de 0,05 a été utilisée pour indiquer la signification statistique des relations.

## Résultats

Cette étude a inclus 457 participants et la séroprévalence de l’AgHBs était de 14,87 %. Les étudiants dont l’âge était compris entre 26 et 35 ans étaient 1,11 fois moins susceptibles de contracter l’infection par le VHB (OR = 0,90, IC 95% : 0,42-1,80; p = 0,032). Les étudiants dont la mère n’était pas infectée par le VHB étaient 2,08 fois moins susceptibles de contracter l’infection par le VHB (OR = 0,48, IC 95% : 0,10-4,26; p = 0,027). Toutefois, les étudiants qui vivaient en colocation ou en famille avaient respectivement 5,41 fois (OR = 5,41, IC 95% : 1,55-25,6; p = 0,025) et 2,69 fois (OR = 2,69, IC 95% : 0,90-11,7; p = 0,025) plus de risque de contracter l’infection par le VHB que ceux qui vivaient seul.

## Conclusion

La prévalence de l’hépatite B est élevée chez les étudiants tchadiens. Nous recommandons de fournir un dépistage, une vaccination contre le VHB ainsi qu’une amélioration des conditions d’habitation des étudiants.

# Research article: 225 - L’hésitation vaccinale pour la Covid-19 et ses motifs au Sénégal, évolutions et discussion de la notion

**Published:** 2024-02-23

**Authors:** Alice DESCLAUX, Khoudia SOW, Équipe CORAF, Sénégal Programme ARIACOV

**Affiliations:** 1 TransVIHMI, Université de Montpellier, IRD, INSERM, France; 2 Centre régional de recherche et de formation à la prise en charge clinique de Fann (CRCF), Dakar, Sénégal

## Objectifs

La vaccination est présentée comme une stratégie incontournable pour mettre fin à la circulation du SARS-CoV-2 et à la pandémie de Covid. Or, cette stratégie se heurte aux réticences des populations que l’OMS qualifie d’hésitation vaccinale, une notion qui recouvre à la fois des positions anti-vaccins très affirmées et des craintes conduisant à différer la vaccination. De nombreuses études ont quantifié cette hésitation aux niveaux local, national et international; des travaux plus larges mais souvent limités aux discours précisent les motifs invoqués par les personnes qui refusent de se faire vacciner. Cette communication vise à présenter les résultats d’une approche ethnographique de l’hésitation vaccinale au Sénégal en 2021, et à comprendre ses déterminants en lien avec les perceptions et les contextes.

## Méthode

Dans le cadre du projet CORAF (Coronavirus Anthropologie Afrique) développé au sein du programme ARIACOV (Appui à la riposte africaine contre le Covid, financé par l’AFD/IRD), nous avons mené des enquêtes par entretiens individuels, certaines répétées, sur les perceptions du vaccin en population et auprès d’acteurs de santé, complétées par une veille continue des médias et des réseaux sociaux centrée sur le vaccin et la vaccination, d’octobre 2020 à décembre 2021. Des enquêtes ont également été menées au Cameroun, au Bénin et au Burkina Faso.

## Résultats

Le suivi quantitatif hebdomadaire de l’acceptabilité du vaccin au Sénégal montre une évolution par phases que nos données qualitatives permettent d’expliquer à partir des contextes. Six phases peuvent être distinguées. Ces phases sont liées notamment à la dynamique du vaccin et de la vaccination (représentations sociales avant, puis après la mise à disposition), aux informations en circulation au niveau global, à la dynamique épidémique (survenue d’une nouvelle vague épidémique) et à l’évolution du dispositif de vaccination (ruptures de stock). Parmi les motifs d’hésitation, certains suivent l’évolution de ces phases, mais d’autres restent présents pendant toute la durée de l’enquête. La comparaison avec les autres pays montre des tendances communes et des différences qui soulignent notamment l’importance des attitudes des professionnels de santé et de l’histoire de la contestation face à d’autres vaccins.

## Conclusion

L’évolution de l’acceptabilité du vaccin au Sénégal au cours de l’année 2021 nous a conduits à proposer des recommandations stratégiques, qui doivent aujourd’hui être remises en contexte et devront être de nouveau discutées en cas de campagne de vaccination de masse face à un variant du SARS-CoV-2. Ces résultats conduisent aussi à discuter la notion d’hésitation et ses modèles explicatifs utilisés en santé globale qui incriminent les attitudes des populations en occultant les aspects limitants des dispositifs tels que les obstacles à l’approvisionnement en vaccins.

# Research article: 120 – Adaptation des soins durant la Covid-19 dans l’unité de prise en charge des enfants et adolescents vivant avec le VIH au Centre hospitalier national d’enfants Albert Royer

**Published:** 2024-02-23

**Authors:** Aichatou DIA, Fatou Kiné NDIAYE, Astou Diop DIÈYE, Fatou NDIAYE, Aminata DIACK, Aida DIAGNE, Mohamed Lamine Ibrahima SOUANÉ, Khady SIDIBÉ, Ndèye Fatou DIALLO, Papa Moctar FAYE

**Affiliations:** Centre hospitalier national d’enfants Albert Royer (CHNEAR), Dakar, Sénégal

## Contexte

Suite à la crise sanitaire liée à la pandémie de Covid-19, les restrictions sur les déplacements à Dakar et en inter-urbain ont engendré des difficultés d’accès aux structures de santé. Au CHNEAR, l’équipe de prise en charge des enfants et adolescents vivant avec le VIH a très tôt modifié l’organisation des soins, dans le but d’assurer à cette population vulnérable une continuité du suivi.

## Objectifs

Ce travail a pour but de décrire les stratégies développées au cours du processus de réorganisation des soins et les conséquences sur la rétention dans les soins et la charge virale (CV).

## Méthode

Des comités médico-techniques ont permis de répartir les patients en 3 groupes : ceux vivant à Dakar, en succès thérapeutique (CV < 1000 copies/ml) pour lesquels la consultation a été décalée de 6 mois et les parents invités à récupérer la dotation en ARV à la pharmacie; ceux vivant à Dakar avec CV élevée (CV > 1000 copies/ml) pour lesquels la visite médicale a été maintenue avec dotation en ARV pour 6 mois; et un troisième groupe constitué par les patients vivant hors de Dakar, pour lesquels les médicaments étaient délivrés par les structures de prise en charge régionales.

L’acheminement des médicaments à domicile ou dans un site de prise en charge proche du domicile a été proposé pour les patients de Dakar ayant éprouvé des difficultés d’accès. Tous les patients pouvaient bénéficier d’une consultation en urgence et un appel téléphonique mensuel était planifié pour rassurer, renforcer en ETP (éducation thérapeutique du patient) et promouvoir les mesures anti-Covid. Toutes ces mesures ont été appliquées de mars à août 2020 et les parents en ont été informés par téléphone. L’évaluation sur la rétention dans les soins et sur la charge virale a été faite à l’issue du processus à partir de septembre 2020.

## Résultats

Les stratégies ont concerné 269 patients. Parmi ceux de Dakar (n = 224), 180 étaient en succès thérapeutique, et près de la moitié (n = 83) ont insisté pour être vus en consultation; 44 avaient une CV élevée, parmi lesquels 27 se sont présentés; 18 patients ont demandé à être dotés en ARV à domicile (n = 3) ou dans une structure proche du domicile (n = 15). Quarante-trois sur les 45 patients vivant hors de Dakar ont bénéficié des mesures. Au bout du processus, le maintien dans les soins a été observé chez 260 patients (96,6 %). La CV mesurée chez 230 patients, avant et après les mesures, montrait une proportion comparable de patients en succès thérapeutique (84,34 % et 85,21 %).

## Conclusion

Ces résultats satisfaisants témoignent de l’utilité de ces mesures d’adaptation pour prévenir la rupture de soins et le maintien en succès thérapeutique.

# Research article: 87 - L’autodépistage au Sénégal… Performances du test oral Sedia Asanté HIV-1/2 aux mains d’utilisateurs formés

**Published:** 2024-02-23

**Authors:** Aminata DIA, Mariama MANE, Mame Khardiata NDIAYE, Thiaba Laye MBAYE, Astou GUEYE, Ibrahima TRAORÉ, Soda NDIAYE, Jean Philippe DIATTA, Papa Alassane DIAW, Souleymane MBOUP

**Affiliations:** 1 Institut de recherche en santé, de surveillance épidémiologique et de formation (IRESSEF), Sénégal; 2 IHS, Institut d’Hygiène Sociale, Dakar, Sénégal; 3 Centre de Conseil Adi, Dakar, Sénégal; 4 District sanitaire de Ziguinchor, Sénégal

## Objectif

Accroître l’accès au dépistage du VIH en utilisant des méthodes novatrices est essentiel pour réduire l’incidence de la transmission et améliorer l’accès au traitement et au soutien pour les personnes vivant avec le VIH. L’autodépistage du VIH est une approche alternative à fort impact, à faible coût et habilitante pour ceux qui ne pourraient pas autrement se faire tester, notamment les populations clés et les autres personnes à risque élevé d’infection par le VIH.

L’objectif principal était d’évaluer la performance du test oral Sedia Asanté™ HIV1/2 lorsqu’il est effectué par un utilisateur professionnel formé (médecin/infirmière) comparée à l’algorithme de test national de référence et un ELISA de 4^e^ génération (EIA), et de confirmer l’interprétation du résultat du test par un deuxième lecteur professionnel en aveugle.

Il s’agit d’une étude prospective, contrôlée et anonyme sur 500 participants de 18 ans et plus. Les participants ont été recrutés dans les sites de dépistage. Les utilisateurs formés ont effectué le test de dépistage du VIH à l’aide d’un prélèvement oral. L’interprétation des résultats par l’utilisateur professionnel a ensuite été comparée aux résultats de divers autres tests de diagnostic rapides (TDR) et ELISA.

Il a été constaté qu’un plus grand nombre de participants à l’étude étaient des femmes (69,2 % contre 30,8 % d’hommes). Les participants âgés de 26 à 35 ans représentaient 38,6 %, 22 % des participants étaient âgés de 36 à 45 ans et les 18 à 25 ans représentaient 19,8 %.

- Autotest VIH vs TDR
  La sensibilité et la spécificité de l’autotest lu par l’utilisateur formé étaient élevées par rapport au résultat du test de confirmation rapide (Determine HIV-1/2). La sensibilité atteignait 100 % et la spécificité était de 100 %. Les tests rapides SD Bioline HIV-1/2 et Multisure HIV-1/2 n’ont été effectués que sur les cas positifs résultant de l’autotest VIH et/ou du test rapide Determine HIV-1/2. Il s’est avéré qu’il y avait 53 infections par le VIH-1, 8 par le VIH-2, et 2 double infections VIH-1 et 2.
- Autotest VIH *vs* ELISA
  En comparant le résultat de l’autotest avec le test ELISA en laboratoire, il y avait 1 cas de discordance (0 faux positif et 1 faux négatif). Conformément au protocole de l’étude, l’échantillon a également été testé à l’aide du test Western Blot et a été déterminé comme ayant p24 seulement.

L’évaluation de la performance clinique de l’autotest Sedia Asanté™ VIH-1/2 utilisant la salive par rapport à l’utilisation professionnelle des tests de diagnostic rapide et des tests immunologiques de 4^e^ génération (EIA) en laboratoire a montré des performances élevées. Sur la base de la performance du test, de la facilité d’utilisation pour les professionnels effectuant le test et de l’interprétation très facile des résultats, Sedia Asanté™ VIH1/2 est une potentielle solution de rechange pour le dépistage du VIH. Il serait intéressant d’évaluer ce test aux mains des utilisateurs non formés.

# Research article: 182 - Évolution de la résistance aux ARV et diversité génétique du VIH entre 2004 et 2015 chez les hommes ayant des rapports sexuels avec d’autres hommes (HSH) au Sénégal

**Published:** 2024-02-23

**Authors:** Diabou DIAGNE, Nafissatou LEYE, Halimatou DIOP-NDIAYE, Yacine DIA, Ousseynou NDIAYE, Ndeye Aminata DIAW, Sada DIALLO, Fatou Maria DRAMÉ, Daouda DIOUF, Souleymane MBOUP, Stefan BARAL, Coumba TOURÉ KANE

**Affiliations:** 1 Institut de recherche en santé, de surveillance épidémiologique et de formation (IRESSEF), Sénégal; 2 Laboratoire de bactériologie et de virologie, Centre hospitalier national universitaire Aristide Le Dantec, Dakar, Sénégal; 3 ENDA Santé, Sénégal; 4 Département de santé publique, Université Johns-Hopkins, États-Unis

## Objectif

Au Sénégal, la résistance du virus de l’immunodéficience humaine (VIH) aux antirétroviraux (ARV) a été largement étudiée dans la population générale avec des taux allant de faibles (< 5 %) à modérés (5-15 %). Cependant, il est important de noter qu’il existe très peu de données décrivant ces résistances parmi les hommes ayant des rapports sexuels avec d’autres hommes (HSH). L’objectif de cette étude était de documenter l’évolution de la résistance et de la diversité génétique du VIH-1 chez les HSH au Sénégal.

## Méthode

Les HSH étudiés provenaient d’une part des enquêtes ELIHOS visant à évaluer les interventions de prévention des IST et du VIH auprès des homosexuels masculins au Sénégal de 2004 et 2007, et d’autre part de l’étude prospective HIV Prévention du 2.0 (HP2) en 2015.

Les séquences de 2004 à 2007 ont été téléchargées de la base de données de HIV Los Alamos (www.hiv.lanl.gov). Pour les HSH de 2015, les échantillons ayant une charge virale (CV) supérieure à 1000 copies/ml ont été extraits par Qiagen et le séquençage Sanger du gène Pol a été réalisé en utilisant la technique ANRS. L’interprétation de la résistance a été effectuée sur l’algorithme de Stanford. Les analyses phylogénétiques ont été faites à l’aide du logiciel SeaView.

## Résultats

Nous avons inclus dans cette analyse 220 séquences dont 69 de 2004, 59 de 2007 et 92 de 2015. La population d’étude était relativement jeune avec un âge médian de 24 ans en 2004, 27 ans en 2007 et 24 ans en 2015. Les taux de résistance étaient de 13,04 % en 2004, 18,64 % en 2007 et 29,35 % en 2015. La mutation E138A associée aux inhibiteurs non nucléotidiques de la transcriptase inverse (INNTI) a été retrouvée majoritairement chez les HSH en 2004, 2007 et 2015. L’analyse phylogénétique des séquences montre une prédominance du sous-type C (40,58 %) suivi du CRF02_AG (26,09 %) et du sous-type B (18,84 %) en 2004. Cependant il faut noter qu’à partir de 2007, il y a une diminution progressive du sous-type C (38,98 % en 2007 et 44,57 % en 2015) et du sous-type B (13,56 % en 2007 et 3,26 % en 2015) au profit du CRF02_AG avec 42,37 % en 2007 et 47,83 % en 2015. La présence de nombreux autres recombinants a aussi été notée.

## Conclusion

Ces résultats montrent une augmentation progressive du taux de résistance aux ARV et une variation de la diversité génétique du VIH chez les HSH au Sénégal. Ces résultats traduisent la nécessité de l’amélioration des interventions ciblées de prévention et de prise en charge pour briser la chaîne de transmission parmi les populations clés, ainsi que d’une surveillance régulière de la résistance au sein de ce groupe.

# Research article: 47 - La bisexualité masculine au Sénégal, entre stratégie de dissimulation et orientation sexuelle assumée : enjeux liés au VIH

**Published:** 2024-02-23

**Authors:** Abdoulaye DIALLO, Gabrièle LABORDE-BALEN, Christophe BROQUA

**Affiliations:** 1 Centre régional de recherche et de formation à la prise en charge clinique de Fann (CRCF), Dakar, Sénégal; 2 TransVIHMI, Université de Montpellier, INSERM, IRD, Montpellier, France; 3 CNRS, Paris, France

## Contexte

Au Sénégal, en 2021, les hommes ayant des relations sexuelles avec des hommes (HSH) sont victimes de stigmatisation, de violences et de poursuites pénales. Contraints de se cacher, ils sont aussi fortement exposés au VIH avec une prévalence de 27 % *versus* 0,3 % dans la population générale. Une partie des HSH sénégalais sont mariés ou entretiennent des relations avec des partenaires féminines. Ces comportements sont souvent considérés comme une « façade » pour éviter d’être identifiés comme homosexuels. Comment les HSH bisexuels sénégalais vivent-ils leurs relations avec leurs partenaires féminines ? Quelles conséquences leur éventuelle séropositivité a-t-elle sur leur conjugalité hétérosexuelle?

## Méthode

Une étude anthropologique a été menée de 2019 à 2021 à Dakar, auprès de 38 HSH âgés de 19 à 45 ans. Des entretiens semi-directifs ont été enregistrés, retranscrits, puis ont fait l’objet d’une analyse de contenu.

## Résultats

Rares sont les personnes rencontrées qui ont des relations exclusivement homosexuelles. La plupart ont aussi des « copines » et d’autres sont même mariés. Les relations avec leurs partenaires féminines sont diverses. Elles constituent chez certains une façade pour rassurer les familles sur leur orientation sexuelle, tandis que chez d’autres, elles traduisent une double attirance. La pression familiale au mariage est forte à partir de 25-30 ans. Le mariage hétérosexuel répond pour certains à un souhait, pour d’autres à une manière de rentrer dans une « normalité » sociale; certains pensent que le mariage réduira leur « envie d’aller voir des hommes ». La plupart n’envisagent pas de dévoiler leur orientation sexuelle à leur épouse.

Le désir d’enfant est général chez tous les HSH de l’étude, même chez ceux qui n’ont pas de partenaires féminines. Certains envisagent l’adoption hors mariage, d’un enfant de la famille. Ceux qui sont dans une démarche de migration projettent une adoption au sein d’une union homosexuelle une fois à l’étranger.

Les HSH séropositifs dévoilent rarement leur statut sérologique dans leur famille ou avec leurs partenaires masculins ou féminins, sauf parfois avec leur mère. Ceux qui envisagent le mariage ont le sentiment d’un dilemme entre le désir de fonder une famille et la crainte de transmettre le virus.

## Conclusion

La bisexualité assumée est la conséquence d’une double attirance et du désir de fonder une famille. Elle est parfois présentée comme une adaptation permettant de répondre à la violence sociale à l’égard de l’homosexualité. Les bisexuels séropositifs expriment leur crainte de transmettre le virus à leur (future) conjointe. Ces observations suggèrent la nécessité d’adapter le dispositif sanitaire et social – écoute, appui à l’observance thérapeutique, médiation familiale – destiné aux HSH afin de prendre en compte la possibilité d’une bisexualité qui n’est pas nécessairement dévoilée et d’accompagner ainsi les personnes dans la diversité de leurs aspirations.

# Research article: 248 - Des associations d’Hommes ayant des relations sexuelles avec d’autres hommes (HSH), lieux de refuge et espaces de conflits devenus indispensables

**Published:** 2024-02-23

**Authors:** Abdoulaye DIALLO, Khoudia SOW, Cheikh Bamba DIEYE, Aissatou SALL, Karim DIOP

**Affiliations:** 1 Centre régional de recherche et de formation à la prise en charge clinique de Fann (CRCF), Dakar, Sénégal; 2 Conseil national de lutte contre le sida et IST (CNLS), Sénégal

## Contexte et objectifs

Dans un contexte social marqué par une hostilité et un rejet social croissant vis-à-vis des homosexuels, les associations de pairs se développent de plus en plus, y compris dans les régions. L’objectif est d’analyser l’évolution des perceptions, approches et activités menées au sein des associations de HSH enquêtées et d’identifier les stratégies de résiliences adoptées.

## Méthodes

Une enquête qualitative a été menée entre décembre 2021 et juillet 2022 dans 8 régions du Sénégal : Dakar, Louga, Saint-Louis, Matam, Tambacounda, Kaffrine, Kaolack et Fatick. 59 entretiens individuels et 15 focus groups ont été effectués auprès des HSH, et 10 entretiens individuels avec le personnel de santé. Au total, 180 HSH et 10 associations de pairs ont été enquêtés.

## Résultats

Ces dernières années, le nombre d’associations identitaires a augmenté, notamment à Dakar et dans quelques régions. À Dakar, diverses associations ont été répertoriées dont l’une a mis en place une cellule transgenre. À Kaolack, au moins 2 structures sont actives alors que Tambacounda, Louga et Saint-Louis n’en comptent qu’une. À Kaffrine et Matam, il n’y en a pas, les activités étant menées par les médiateurs. Les associations sensibilisent les membres sur la prévention du VIH, orientent et facilitent l’accès aux services sanitaires tout en fournissant un appui psychosocial ou financier notamment dans des cas d’expulsion, ou de diverses violences dont sont victimes leurs membres. Elles servent de lieu de refuge, d’empathie et de partage pour les HSH en leur permettant de surmonter leur peur, leur isolement et de raffermir les liens entre pairs ou faciliter des rencontres de partenaires. Certains espèrent également des appuis pour émigrer. Dans de nombreuses régions, les associations qui évitent les rassemblements ont de plus en plus de mal à mener leurs activités au sein d’un local dédié, par peur d’être identifiés et agressés. De plus en plus, les activités sont virtuelles à travers les réseaux sociaux ou sont menées avec les médiateurs dans les structures de santé. Au fil des années, les leaders associatifs rapportent les sollicitations croissantes de leurs membres qui font face à de multiples contraintes psychologiques, sociales, financières ou professionnelles. En outre, les nombreux cas de rejet, d’exclusion, de difficulté d’accès aux soins ou de poursuites pénales contre des HSH poussent un nombre croissant d’entre eux à se mettre « sous protection » des associations. Or certains enquêtés rapportent que l’adhésion associative induit une visibilité qui expose des personnes jusque-là soucieuses de préserver la confidentialité de leur orientation sexuelle, et divers conflits entre pairs sont assez fréquents. Dans ce contexte certains HSH, notamment les plus âgés ou les plus nantis, évitent ces espaces tout en gardant un lien avec un acteur associatif.

## Conclusion

Dans un contexte d’exacerbation de violences multiformes contre les HSH, les associations sont de plus en plus indispensables. Elles constituent des lieux d’expression et de socialisation entre pairs HSH, mais la nécessité de travailler dans une forme de clandestinité et les sollicitations de soutien de leurs membres constituent des restrictions majeures à leur efficacité.

# Research article: 58 - Infection à VIH-2 au Sénégal : prévalence et échecs virologiques chez les patients de la cohorte CARES de la région de Ziguinchor

**Published:** 2024-02-23

**Authors:** Kalilou DIALLO, Mame Aïssé THIOUBOU, Henri GOEDERTZ, Chabi BINDIA, Jérome-Henri DIAGNE, Benjamin Amaye SAMBOU, Ousseynou CISSÉ, Tito TAMBA, Vic ARENDT, Daouda DIOUF, Carole DEVAUX, Noël Magloire MANGA

**Affiliations:** 1 Hôpital régional de la Paix, Ziguinchor, Sénégal; 2 Université Assane Seck, Ziguinchor, Sénégal; 3 ONG SAN ACCESS, Luxembourg; 4 ENDA Santé, Sénégal; 5 Centre de santé de Ziguinchor, Sénégal; 6 Centre de Santé de Bignona, Sénégal; 7 Luxembourg Institute of Health; 8 Centre hospitalier de Luxembourg, Luxembourg

## Objectifs

Évaluer la prévalence du VIH-2 et déterminer les facteurs associés aux échecs thérapeutiques chez les patients sous traitement antirétroviral depuis au moins 12 mois dans la région de Ziguinchor.

## Patients et méthodes

Étude de cohorte multicentrique descriptive et analytique portant sur les patients suivis dans 4 sites de dispensation d’ARV de la région (Centres de santé de Bignona et Ziguinchor, Hôpital régional et de la Paix), réalisée dans le cadre du programme CARES du 1^er^ janvier 2019 au 30 septembre 2021. L’échec virologique a été défini comme toute charge virale CV > 1000 cp/ml après 6 mois de traitement ARV. Les données ont été saisies et analysées grâce aux logiciels EpiData et Stata 16.

## Résultats

Sur 2071 patients inclus, nous avons colligé 251 cas d’infection à VIH-2 soit une prévalence de 12,12 %. La majorité des patients étaient de sexe féminin (79,28 %), soit un sex-ratio F/H = 3,8. L’âge moyen était de 51 ± 12 ans [12-84 ans] avec une prédominance de la tranche d’âge de 45 à 60 ans (49 %). L’association VIH-1 et VIH-2 était notée chez 44 patients (2,12 %). La prévalence de la co-infection VIH-VHB était de 7,5 %. Le schéma antirétroviral associait 2 INTI (inhibiteurs nucléosidiques de la transcriptase inverse) à l’IP (inhibiteur de protéase) chez 80,5 % des cas et 2 INTI + DTG (dolutégravir) chez 6 % des cas. La charge virale réalisée chez 122 (48,6 %) patients était indétectable.

## Conclusion

La prévalence du VIH-2 reste faible comparée au VIH-1. L’échec virologique au cours de cette infection est important dans notre cohorte, dont l’identification des facteurs déterminants devra faire l’objet d’études de plus grande envergure.

# Research article: 54 - Évaluation de l’aspect clinique et de la prévalence du VIH chez les populations clés (hommes ayant des relations sexuelles avec d’autres hommes et professionnels du sexe) à bord de la clinique mobile de Mbour

**Published:** 2024-02-23

**Authors:** Papa Amadou DIALLO, Moussa BA, Makhoudia NIANG, Anne Marie MENDY, Ismaila CAMARA, Maimouna NDIAYE, Mamadou Lamine SAM, Ibrahima Khalil DIAW

**Affiliations:** 1 District sanitaire de Ziguinchor, Sénégal; 2 District sanitaire de Mbour, Sénégal; 3 Établissement de santé publique de Grand-Mbour, Sénégal; 4 ANCS (Alliance nationale des communautés pour la santé), Dakar, Sénégal

## Contexte

L’épidémie de VIH au Sénégal est de type concentré, avec une prévalence basse dans la population générale estimée à 0,4 % en 2020 (CNLS, 2020) et élevée dans les populations clés les plus exposées au risque du VIH – 5,8 % chez les professionnelles du sexe (PS), rapport ENSC 2019; 27,6 % chez les hommes ayant des relations sexuelles avec les hommes (HSH) en 2017, ELIHoS. Si la proportion de PvVIH qui ignorent leur statut sérologique diminue, l’atteinte des quelques personnes restantes qui sont asymptomatiques et qui ne sont pas en contact avec le système de santé reste un défi crucial. C’est dans ce contexte qu’une clinique mobile a été mise en place à Mbour pour atteindre et prendre en charge les populations difficiles d’accès. L’objectif de cette communication est d’évaluer la prise en charge à bord de la clinique mobile.

## Méthodologie

Il s’agit d’une étude rétrospective descriptive à partir des informations recueillies dans les dossiers médicaux de patients VIH, des registres de consultation et de laboratoire dans la clinique mobile et des fiches de séances de dépistage du 25 décembre 2019 au 20 janvier 2020. Nous avons inclus dans notre étude tous les patients qui ont fréquenté la clinique mobile et qui ont bénéficié au moins d’un dépistage. La clinique mobile comporte une équipe pluridisciplinaire, à savoir des agents de santé (médecins, assistants sociaux, laborantins, sages-femmes et infirmiers renforcés sur la prise en charge du VIH en cas d’absence du médecin) et des acteurs communautaires (superviseur sur site chargé de coordonner les déplacements de la clinique, des relais communautaires médiateurs de proximité et un chauffeur). La voiture banalisée comporte : un siège avant destiné au conseil, à l’arrière une table de consultation, une source lumineuse, un équipement de laboratoire, des espaces de stockage pour les intrants et une source d’eau.

## Résultats

Au total, nous avons reçu 40 clients à bord de la clinique. L’âge moyen était de 25,15 ± 5,88 ans avec une médiane de 23 ans. Les extrêmes variaient entre 19 et 42 ans. Il y avait une prédominance masculine (31 soit 77,5 %) avec un sex-ratio de 3,44. Parmi les patients, 85 % étaient célibataires (34).

La moitié (50 %) de la population d’étude avaient déjà réalisé un test de dépistage VIH au cours de leur vie. L’utilisation de préservatif n’était pas systématique dans 80 % des cas; 30 % des personnes ont eu recours aux drogues; 92,5 % de la population d’étude avaient de multiples partenaires sexuels. Le taux de séropositivité était de 35 %, soit 14 patients dans la population d’étude. Il était de 41,94 % (13) chez les HSH et de 11,11 % (1) chez les PS (p = 0,0514). Tous les patients séropositifs étaient de profil VIH-1 et prenaient un traitement TLD (ténofovir, lamivudine et dolutégravir) avec 100 % de lien au traitement. Le stade clinique moyen selon l’OMS était de 1,5 ± 0,5, variant de 1 à 2. Les infections opportunistes digestives étaient les plus fréquentes (28,57 %) à savoir candidose buccale, diarrhée; 25 % des patients étaient atteints d’IST dont des douleurs abdominopelviennes, des écoulements et des infections urinaires (5 % pour chaque pathologie). D’autres pathologies étaient rencontrées à 7,5 % pour les maladies hémorroïdaires et 5 % chacun pour les prurits oculaires et la lombalgie.

## Conclusion

La clinique mobile est une stratégie capitale permettant de dépister des personnes habituellement difficiles à atteindre, et de les traiter pour le VIH et les infections opportunistes. L’environnement juridicosocial peut constituer un obstacle à cette stratégie.

# Research article: 22 - L’expérience des personnes ayant le « Covid long » au Sénégal

**Published:** 2024-02-23

**Authors:** Thierno Madiou DIALLO, Alice DESCLAUX, Khoudia SOW

**Affiliations:** 1 Centre régional de recherche et de formation à la prise en charge clinique de Fann (CRCF), Dakar, Sénégal; 2 Institut de recherche pour le développement (IRD)

## Objectifs

Le Covid long est « une affection qui survient chez des personnes présentant des antécédents d’infection probable ou confirmée de Covid-19, généralement 3 mois après l’apparition de la phase aiguë avec des symptômes qui persistent au moins 2 mois et qui ne peuvent être expliqués par un autre diagnostic » (OMS, 2021). Plusieurs études réalisées en Europe et aux États-Unis montrent la présence de symptômes chez plus de 20 % des patients après 5 semaines de guérison et chez plus de 10 % des patients après 3 mois. En Afrique, les études en santé publique ou sciences sociales sur le Covid long sont rares. Cette communication vise à décrire l’expérience de personnes présentant les symptômes d’un « Covid long » au Sénégal.

## Matériels et méthodes

Dans le cadre du projet CORAFMOB/ANRS (documentation des mobilisations communautaires face au Covid-19 et ses enjeux sociaux au Sénégal et au Burkina Faso), une recherche qualitative a été menée pour analyser l’expérience des patients Covid ayant été pris en charge en extra-hospitalier dans l’un des Centres de traitement des épidémies (CTE) de Dakar. Des entretiens individuels approfondis ont été réalisés à Dakar d’août 2020 à février 2021 avec des personnes ayant séjourné dans ce CTE. Les répondants ont été interrogés 15 jours après leur sortie du CTE puis 6 mois plus tard. Les entretiens ont été enregistrés puis transcrits et ont fait l’objet d’une analyse thématique de contenu avec Word. Les précautions d’éthique usuelles ont été respectées (autorisation du comité d’éthique, consentement éclairé des participants, anonymisation, respect de la confidentialité).

## Résultats

Parmi les 53 répondants, après 6 mois de guérison, 11 personnes ont eu des symptômes persistants dont 7 femmes et 4 hommes. Les principaux symptômes persistants identifiés sont : une fatigue chronique, des insomnies, un essoufflement, des maux de tête et une sensation d’oppression. Face à cette situation, le discours médical est axé sur l’origine psychologique de ces symptômes, et aucune stratégie de prise charge n’est proposée. Livrées à elles-mêmes, les personnes présentant les symptômes d’un Covid long se tournent vers l’automédication et les réseaux sociaux pour chercher des réponses, une écoute et du soutien. La plupart ont vu leur vie socio-professionnelle fortement impactée par le Covid long : baisse de leur capacité de concentration, des absences répétées au travail, une mauvaise qualité du sommeil, une réduction des activités de loisir et une fatigabilité lors des activités de la vie courante.

## Conclusion

Malgré le fort impact socioprofessionnel, le Covid long reste invisible et méconnu des acteurs sanitaires. Ces observations interrogent sur la prise en charge et l’accompagnement psychosocial des « guéris » ou « survivants » d’une épidémie qui sont la plupart du temps abandonnés et livrés à leur propre sort. Ces résultats interpellent les professionnels de santé, les autorités sanitaires et les associations de personnes atteintes de Covid-19 ainsi que la société civile.

# Research article: 242 - Invisibilisations, normalisations et nouvelles vulnérabilités chez les travailleuses du sexe au Sénégal

**Published:** 2024-02-23

**Authors:** Thierno Madiou DIALLO, Babacar THIAM, Safiatou THIAM, Christine Awa DIOUF, Khoudia SOW

**Affiliations:** 1 Centre régional de recherche et de formation à la prise en charge clinique de Fann (CRCF), Dakar, Sénégal; 2 Comité de suivi, de veille et d’alerte, Sénégal; 3 Conseil national de lutte contre le sida et les IST (CNLS), Sénégal

## Objectif

Au Sénégal, la prévalence du VIH chez les travailleuses du sexe (TS) demeure élevée (5,8 % en 2019) en raison de différentes formes de vulnérabilité qui évoluent et sont peu connues. L’objectif de cette communication est de décrire les modalités d’évolution du travail du sexe et d’analyser les formes de vulnérabilité au VIH qui persistent.

## Méthodologie

L’étude a été menée dans le cadre d’un projet sur les populations clés supervisé par le CNLS et financé par le FM (Fonds mondial de lutte contre le sida). Une approche qualitative combinant des entretiens individuels (32) et des focus groups (17) a été adoptée. La collecte de données s’est déroulée entre février et juin 2022 dans 8 régions (Dakar, Saint-Louis, Louga, Matam, Kaolack, Kaffrine, Fatick et Tambacounda). Au total, 156 TS et 25 agents de santé ont été interrogés. Les données ont été analysées à l’aide de l’application Dedoose.

## Résultats

Les TS rencontrées, âgées de 20 à 50 ans, sont majoritairement divorcées ou veuves avec des enfants en charge. Pour la plupart d’entre elles, le travail du sexe est réalisé à temps partiel en complément des « petits boulots » (commerce, restauration…). Les TS enquêtées ont expliqué la quasi-disparition du racolage pour de nouvelles formes de mise en contact avec les clients par l’intermédiaire des réseaux sociaux. Elles s’associent entre pairs pour louer des appartements afin d’y recevoir les clients à des heures précises avec l’aide de numéros téléphoniques dédiés exclusivement à leurs activités soutenues par des « courtiers » qui servent d’intermédiaires avec les clients des sites Internet. Les rencontres avec les clients se font le plus souvent durant la journée et de moins en moins la nuit. Certaines des TS ont rapporté exercer leur activité dans des salons de massage. Les femmes enquêtées attestent des changements en expliquant que les jeunes générations de TS ne fréquentent quasiment plus les bars ou espaces de nuit, qu’elles ont adopté un port vestimentaire banal, et que l’usage de drogue et d’alcool tend à diminuer. Ces données peuvent être analysées comme des stratégies de dissimulation, de normalisation voire de professionnalisation du travail du sexe avec l’aide des nouvelles technologies de l’information et de la communication. Cette invisibilisation du travail du sexe laisse apparaître de nouvelles formes de vulnérabilité. En effet, les femmes enquêtées ont rapporté qu’elles subissent des chantages de la part des gestionnaires des sites Internet et peuvent être filmées à leur insu et exposées au risque de la divulgation des vidéos et des images. De plus, cette invisibilisation renforce la clandestinité des pratiques qui rende les TS peu accessibles aux interventions de prévention contre le VIH, au suivi médical régulier alors qu’elles expliquent avoir de plus en plus de mal à accéder à des préservatifs gratuits et que certains de leurs clients leur promettent plus d’argent pour des relations non protégées en leur proposant parfois de faire un test rapide de VIH.

## Conclusion

Au Sénégal, les évolutions dans le travail du sexe font apparaître de nouvelles vulnérabilités socio-sanitaires qu’il est impératif de prendre en compte dans les interventions du programme sida.

# Research article: 29 - Rôle des Jeunes leaders transformationnels dans l’amélioration de la santé sexuelle et reproductive des adolescentsjeunes dans le district sanitaire d’Oussouye de janvier 2019 à décembre 2020

**Published:** 2024-02-23

**Authors:** Diombraise Gabriel DIATTA

**Affiliations:** Conseil national de lutte contre le sida (CNLS), Sénégal Gestionnaire des données VIH/IST, District sanitaire d’Oussouye

## Introduction

Au Sénégal, selon l’EDS V (Enquête démographique et de santé), les rapports sexuels sont précoces avec l’âge du 1^er^ rapport entre 10 et 12 ans chez les filles et garçons, 16 % des filles sont victimes de grossesses précoces non désirées, et 23,5 % d’avortements clandestins sont enregistrés chez les adolescentes-jeunes. Certaines filles sont victimes de violences et d’abus sexuels, en particulier les moins de 15 ans parmi lesquelles 17,5 % ont été excisées. Le taux de prévalence contraceptive est très faible chez les filles de 15 à 19 ans avec 6,6 % en 2018. D’autre part, 79 % des filles ont des complications liées à l’accouchement.

Ce constat a motivé l’adoption de stratégies comprenant la réorganisation des services, l’aménagement des espaces d’accueil et le renforcement des capacités des prestataires et des acteurs communautaires à offrir des services conviviaux de santé sexuelle et reproductive aux adolescents-jeunes.

Le district sanitaire d’Oussouye se trouve dans la région de Ziguinchor où les adolescents-jeunes ont un accès limité à l’information et aux services de santé reproductive de qualité avec 31,1 % des besoins non satisfaits (EDS V), ce qui les expose à des comportements à risque.

## Objectif

Décrire le rôle des Jeunes leaders transformationnels (JLT) dans l’amélioration de la santé sexuelle et reproductive des adolescents-jeunes dans le district sanitaire d’Oussouye.

## Méthodes

Il s’agissait d’une étude descriptive des stratégies mises en œuvre par les JLT. Ils étaient sélectionnés sur la base de leur engagement et formés en leadership transformationnel et sur les techniques de communication avec leurs pairs.

Les activités étaient axées sur le plaidoyer, les émissions radios, les forums et les entretiens individuels. La cible était les adolescentsjeunes (10-24 ans).

Les données collectées sont issues des rapports périodiques fournis par le district.

## Résultats

Au total, les activités ont concerné 8 journées de plaidoyer auprès des collectivités territoriales et conseils municipaux, 24 émissions radios, 12 forums et 1800 entretiens individuels. Ces activités ont permis à 3000 ados-jeunes de connaître leur statut sérologique VIH, d’enrôler 10 jeunes séropositifs dans les soins VIH, de diagnostiquer et traiter 1183 cas d’infections sexuellement transmissibles, de distribuer 5873 préservatifs et 1000 lubrifiants, d’enrôler 600 adolescentes-jeunes dans la planification familiale. Le taux de prévalence contraceptive chez les ados-jeunes est passé de 10,05 % en 2019 à 15,17 % en mars 2021, soit une hausse de 5,12 %.

## Conclusion

Les JLT jouent un rôle important dans l’accès à l’information et aux services de santé sexuelle et reproductive des adolescents-jeunes. Cela s’est traduit par l’amélioration des indicateurs de la santé sexuelle et reproductive. Dans cette perspective, il est opportun de mettre en place des mécanismes adéquats visant au renforcement de la mise en œuvre, à l’extension et à la pérennisation de l’initiative « Jeunes leaders transformationnels » dans tous les programmes de santé.

# Research article: 28 - Le parrainage par les prestataires : une stratégie communautaire d’amélioration de la prise en charge des enfants et adolescents VIH suivis au niveau du district sanitaire d’Oussouye (Sénégal)

**Published:** 2024-02-23

**Authors:** Diombraise Gabriel DIATTA

**Affiliations:** Conseil national de lutte contre le sida (CNLS), Sénégal Assistant social, Gestionnaire des données VIH/IST, District sanitaire d’Oussouye

## Introduction

Les résultats de l’enquête EnPRISE en 2015 ont montré un fort taux d’échec thérapeutique (64 %) chez les enfants/adolescents vivant avec le VIH (EAv-VIH), sous traitement ARV, suivis dans un contexte décentralisé. 60 % des enfants de l’étude étaient orphelins d’au moins un des parents. L’absence de répondant fiable serait une des raisons de l’échec du suivi de ces enfants/adolescents.

En 2019, une stratégie dénommée « Tutorat des enfants vivant avec le VIH » a été initiée par la Division de lutte contre le sida et les IST (DLSI) à l’échelle nationale. Elle vise à améliorer le suivi des enfants et adolescents, en désignant des prestataires de soins comme tuteurs pour assurer un accompagnement médical et social.

L’objectif de cette étude est d’évaluer la mise en œuvre de cette stratégie dans le district sanitaire d’Oussouye.

## Méthodologie

Il s’agit d’une étude descriptive et transversale de la stratégie du Tutorat, mise en œuvre auprès de 14 adolescents et enfants parrainés au district sanitaire d’Oussouye. Pour chaque enfant/adolescent, un diagnostic social est réalisé. Il prend en compte : le respect des rendez-vous, l’observance, l’état nutritionnel, l’évolution de la croissance, l’évolution clinique, la présence d’infections opportunistes, les problèmes de scolarisation… Les parents ou tuteurs légaux sont informés et signent un consentement éclairé avant l’inclusion de l’enfant dans le programme.

Les prestataires concernés sont des médecins, assistants sociaux, sages-femmes, infirmiers et médiateurs du district sanitaire d’Oussouye. Ils s’engagent à devenir tuteur secondaire d’un ou plusieurs enfants (3 au maximum). Les activités menées sont : visites à domiciles, appels téléphoniques, distribution communautaire d’ARV, fourniture de kits alimentaires et scolaires. Les tuteurs veillent aussi au respect du suivi médical, à l’observance en particulier du prélèvement régulier pour mesurer la charge virale (CV).

## Résultats

Tous les prestataires de soins engagés dans le programme ont parrainé au moins 1 enfant. De janvier à novembre 2020, 73 appels téléphoniques pour rappel de rendez-vous et 102 visites à domicile ont été effectués pour le suivi psychosocial, et 42 sorties de distribution communautaire ont été réalisées pour les 14 enfants inscrits dans ce programme. Des appuis alimentaires ont été fournis à 12 enfants et des kits scolaires à 9 enfants. Le suivi médical a été renforcé, 14 enfants ont bénéficié d’un prélèvement pour mesurer la CV. Les résultats ont montré que 10/14 (74 %) enfants ont une CV indétectable, ce qui démontre une bonne observance.

## Conclusion

Le parrainage des enfants/adolescents vivant avec le VIH apporte un appui social et nutritionnel. Il permet un meilleur suivi sur le plan médical, favorise le respect des rendez-vous, l’observance des traitements ARV et la suppression de la charge virale.

La pérennisation de telles interventions pourrait contribuer à une meilleure rétention dans les soins des enfants et adolescents vivant avec le VIH.

# Research article: 96 - La mise en réseau des acteurs de lutte contre le VIH pour la création d’experts locaux et le partage d’expériences sur les populations clés en Afrique de l’Ouest

**Published:** 2024-02-23

**Authors:** Ngoundji DIENG, Sidy Mokhtar NDIAYE, Nguissali TURPIN, Fatou Maria DRAMÉ, Ibrahima BÂ, Daouda DIOUF

**Affiliations:** ENDA Santé

## Description du problème

Les acteurs communautaires en Afrique de l’Ouest bénéficient de plusieurs programmes de renforcement de capacités dans le domaine du VIH, mais le suivi et la capitalisation des savoirs et expériences acquis ont toujours été un défi. À travers son projet « Renforcement de capacités et capitalisation des expériences pour une meilleure prise en charge du VIH en Afrique de l’Ouest (ReCCAP) », ENDA Santé a procédé à la mise en place de réseaux d’acteurs intervenant sur le VIH, bénéficiant de formations sur la cartographie et l’estimation de la taille des populations clés dans les pays de mise en œuvre du projet (Côte d’Ivoire, Guinée, Guinée-Bissau et Sénégal). L’objectif est de former progressivement un réseau d’experts locaux en vue d’initier un partage d’expériences Sud-Sud sur les domaines du VIH et des populations clés en Afrique de l’Ouest.

## Activités réalisées et résultats

À la suite de chaque formation régionale et nationale de renforcement de capacités sur la cartographie et l’estimation des tailles des populations clés et sur la Covid-19, la coordination du projet organise la mise en réseau des bénéficiaires à travers des plateformes virtuelles. Ces dernières mettent en relation plusieurs profils d’acteurs impliqués dans le VIH : professeurs d’université, formateurs, médecins en charge des PvVIH, membres des ONG, prestataires de soins, personnes vivant avec le VIH, populations clés, etc. Ces espaces de discussion complètent la formation et constituent un relais dans la diffusion d’informations et d’activités des projets en lien avec les populations clés, comme l’organisation de webinaires.

Au total, 18 plateformes virtuelles (dont 17 groupes WhatsApp et la plateforme digitale www.carto4health.org) ont été créées en Côte d’Ivoire, en Guinée, en Guinée-Bissau et au Sénégal, soit près de 400 acteurs impliqués dans le VIH.

Elles ont favorisé la mise en place de pools d’experts locaux interconnectés sur les domaines de la cartographie et de l’estimation de la taille des populations.

Elles ont permis la vulgarisation d’initiatives innovantes et le partage d’information sur les thématiques du VIH et des populations clés. Elles ont renforcé les liens entre les acteurs universitaires et gouvernementaux, les acteurs de la société civile et les populations clés.

## Leçons apprises et étapes futures

La mise en place de ces réseaux d’acteurs intervenant dans la lutte contre le VIH est capitale, surtout dans un contexte hostile aux populations clés pour l’accès à l’information, au partage de bonnes pratiques dans des expériences et études portant sur les populations clés. Ces plateformes nous ont permis de maintenir la dynamique de groupe et la perpétuation du savoir acquis durant les formations régionales et nationales sur les populations clés.

# Research article: 163 - Dispensation des autotests de dépistage du VIH : quels apports des dispensateurs indépendants dans l’atteinte du 1^er^ 95 ?

**Published:** 2024-02-23

**Authors:** Penda DIENG, Nguissali TURPIN, Katy DIOP, Aminata DIALLO, Ibrahima BÂ, Sidy Mokhtar NDIAYE, Fatou Kiné SOGODOGO

**Affiliations:** ENDA Santé

## Contexte et objectif

Le Sénégal connaît une épidémie concentrée avec une prévalence nationale de 0,5 % dans la population générale (EDSC – Enquête démographique et de santé continue – 2017), 27,6 % chez les HSH (ECHSH – Enquête combinée chez les HSH – 2017), 5,8 % chez les professionnels du sexe (ENSC – Enquête nationale de surveillance combinée – 2019) et 5,2 % chez les consommateurs de drogues injectables (UDSEN – Usager de drogue Sénégal, ANRS12243 – 2011). L’objectif des 3x95 est encore loin d’être atteint et d’importants efforts restent à fournir. Fin 2018, suivant la cascade de soins, seules 72 % des PvVIH connaissaient leur statut (GAM – Global AIDS Monitoring – 2018). Le recours au dépistage est particulièrement faible dans certaines populations clés. C’est dans ce contexte que le projet ATLAS est venu contribuer à l’atteinte du 1^er^ 95 en mettant à disposition des autotests de dépistage du VIH aux populations vulnérables et cachées.

Ainsi le volet communautaire du projet ATLAS, porté par ENDA Santé, vise, à travers l’autotest, à contribuer à atteindre les populations cachées dans les régions de Dakar, Thiès et Ziguinchor.

L’objectif de cette approche est d’améliorer l’accès au dépistage des populations clés – professionnels du sexe (PS), hommes ayant des relations sexuelles avec d’autres hommes (HSH) et usagers de drogues (UD).

## Activités réalisées et résultats obtenus

Au total, 30 dispensateurs indépendants ont été choisis dans les 3 régions de Dakar, Thiès et Ziguinchor (18 PS, 10 HSH et 2 UD). Ils ont été formés sur la dispensation des autotests avant démarrage au niveau communautaire. Des ateliers ont également été organisés pour une mise à jour de la cartographie des points chauds ou sites de rencontres des populations clés. Les dispensateurs ont effectué des sorties nocturnes au niveau des points chauds et des visites à domicile.

Sur la période allant de juillet 2019 à décembre 2021, toutes stratégies confondues (associatif, indépendant et clinique mobile), 45 296 autotests de dépistages du VIH (ADVIH) ont été dispensés par le volet communautaire, dont 28 247 réalisés par les indépendants soit 62 % du nombre total d’autotests distribués au niveau communautaire – 64,5 % (18 256/28 247) *via* le canal des PS, 32,9 % (9 305/28 247) *via* le canal des HSH et 2,4 % (686/28 247) *via* le canal des UD.

## Leçons apprises et étapes futures

Ces différents résultats montrent que plus de la moitié des ADVIH ont été dispensés par les pairs éducateurs indépendants à travers les visites à domicile et sorties nocturnes. Ainsi cette approche a permis d’offrir l’autotest à un nombre important de populations clés, surtout celles ayant un faible accès aux services de prévention.

Cette approche pourrait être utilisée par d’autres intervenants dans la réponse au VIH pour accélérer l’atteinte du 1^er^ 95 au niveau communautaire. Toutetois le dispositif de suivi mis en place n’a pas permis de capitaliser la totalité des cas réactifs. Aussi un renforcement de capacités permettrait à ces dispensateurs d’améliorer les résultats obtenus.

# Research article: 77 - Profil des patients tuberculeux séronégatifs au VIH hospitalisés à la Clinique des maladies infectieuses de Fann, Dakar (Sénégal) : prévalence et facteurs associés au décès

**Published:** 2024-02-23

**Authors:** Alassane DIEYE, Alassane SARR, Samba NIANG, Amadou Diop DIA, Diatou DIAGUEYE, Daye KA, Viviane Marie Pierre CISSÉ, Ndèye Aissatou LAKHE, Khardiata DIALLOMBAYE, Seynabou LO, Ndéye Méry DIA-BADIANE, Moussa SEYDI

**Affiliations:** 1 UFR des Sciences de la santé, Université Gaston Berger, Saint-Louis, Sénégal; 2 Service de médecine, Centre hospitalier régional de Saint-Louis, Sénégal; 3 Service des maladies infectieuses et tropicales, Centre hospitalier national universitaire de Fann, Dakar, Sénégal

## Introduction

La tuberculose est une maladie bactérienne, contagieuse, à transmission principalement aérienne. Elle est responsable d’une morbi-mortalité très importante même chez les patients séronégatifs au VIH. L’objectif de cette étude était de décrire les aspects épidémiologiques, cliniques, paracliniques, thérapeutiques, évolutifs et d’identifier les facteurs associés au décès chez les patients tuberculeux non infectés par le VIH suivis à la Clinique des maladies infectieuses de Fann.

## Méthodologie

Étude transversale, rétrospective, descriptive et à visée analytique allant du 1^er^ janvier 2018 au 31 décembre 2019.

## Résultats

Cent trente patients ont été inclus avec une prévalence hospitalière de 7,4 %. L’âge moyen était de 38 ans (± 17 ans) avec un sex-ratio de 2,1. Quatre-vingts patients (61,5 %) provenaient de la banlieue dakaroise. Sur le plan professionnel, 59 patients (45,4 %) étaient sans emploi. Les signes classiques de la tuberculose ont été retrouvés : toux chronique (67,7 %), fièvre vespéro-nocturne (97,7 %), altération de l’état général (93,1 %). La tuberculose pulmonaire était la plus représentée (75,4 %). La TB ganglionnaire était la localisation extra-pulmonaire la plus fréquente (36,2 %). La bacilloscopie était positive chez 38 patients (35,2 %). Le GeneXpert’ MTB/RIF (Cepheid, Californie) était positif chez 33 patients (45,2 %). Le délai moyen de mise sous traitement était de 4,5 jours (± 3). Le traitement antituberculeux (RHZE) était instauré chez 123 patients (94,6 %). La durée d’hospitalisation moyenne était de 17 jours (± 12). Les complications rencontrées chez 71 patients (54,6 %) étaient dominées par la surinfection et les troubles ioniques avec 27 cas chacun (20,8 %). L’évolution était favorable dans 94 cas (72,3 %) et le décès déploré dans 32 cas (24,6 %). Les facteurs associés au décès en analyse multivariée étaient : le diabète sucré (p = 0,04); la non-mise sous traitement (p = 0,006); le délai de mise sous traitement supérieur à 10 jours (p = 0,05); la survenue de complications en cours d’hospitalisation (p = 0,002); la durée d’hospitalisation supérieure à 20 jours (p = 0,001).

## Conclusion

Notre travail avait montré que la tuberculose reste une maladie grave même chez les patients non infectés par le VIH malgré la gratuité du traitement. La mortalité reste élevée dans notre série et était liée à plusieurs facteurs. Une meilleure connaissance de ces facteurs permettrait de mieux orienter les stratégies de prise en charge afin de réduire la morbi-mortalité liée à la tuberculose.

# Research article: 121 - Profil des hommes ayant des rapports sexuels avec des hommes infectés par le VIH suivis au Centre hospitalier régional de Saint-Louis (Sénégal)

**Published:** 2024-02-23

**Authors:** Alassane DIEYE, Alassane SARR, Amadou Diop DIA, Diatou DIA-GUEYE, Demba MAKALOU, Ndeye Fatou DIEDHIOU, Ndeye Penda YADE, Mamoune NDIAYE, Ahmed DIENG, Seynabou LO, Ndeye Méry DIA-BADIANE

**Affiliations:** 1 UFR des Sciences de la santé (UFR2S), Université Gaston Berger, Saint-Louis, Sénégal; 2 Service de médecine, Centre hospitalier régional de Saint-Louis, Sénégal

## Introduction

Le contrôle de l’infection dans les populations clés est incontournable pour espérer une élimination de l’infection d’ici 2030. Au Sénégal, l’épidémie est de type concentré avec une forte prévalence dans les populations clés notamment chez les hommes ayant des rapports sexuels avec des hommes (HSH). L’objectif de cette étude était de décrire les aspects épidémiologiques, cliniques, immuno-virologiques, thérapeutiques et évolutifs de l’infection à VIH chez les HSH suivis au Centre hospitalier régional (CHR) de Saint-Louis (Sénégal).

## Méthodologie

Étude de cohorte rétrospective, transversale, descriptive et à visée analytique chez les HSH séropositifs au VIH sous traitement ARV, suivis au CHR de Saint-Louis, sur la période de mai 2006 à août 2022.

## Résultats

Nous avons colligé 131 dossiers de HSH parmi les 333 patients suivis dans notre cohorte, soit une prévalence de 39 %. L’âge moyen était de 30 ans (± 13,2). La tranche d’âge la plus représentative était comprise entre 16 et 30 ans avec 83 cas (63,4 %). Nos patients étaient des célibataires dans 108 cas/129 (83,7 %). Ils étaient instruits dans 98 cas/103 (95 %). La majorité des patients exerçaient dans le secteur informel avec 79 cas/125 (63,2 %). La principale porte d’entrée aux soins était le dépistage volontaire avec 97 cas/131 (74 %). À l’inclusion, les stades 1 et 3 de l’OMS étaient prédominants avec respectivement 94 cas/118 (79,7 %) et 10 cas/118 (8,5 %). L’indice de masse corporel moyen actuel [21,3 kg/m^2^ (± 3,4)] était supérieur à celui de l’inclusion [19,8 kg/m^2^ (± 3,4)] (p = 0,4). Le VIH-1 prédominait avec 129 cas (98,5 %). Le taux moyen de lymphocytes TCD4+ actuel [421 cellules/mm^3^ (± 255)] était supérieur à celui de l’inclusion [387 cellules/mm^3^ (± 328)] (p = 0,4). La charge virale moyenne était de 3115 copies/ml (± 11 212). Une charge virale indétectable (CV < 50 copies/mm^3^) était retrouvée chez 57 patients/77 (74 %). L’AgHBs était positif chez 6 patients/64 soit une sérop révalence de 9,4 %. Le schéma d’ARV le plus prescrit était TDF-3TC-DTG avec 84 cas (64,1 %). Les chimioprophylaxies au cotrimoxazole et à l’INH étaient prescrites respectivement dans 27 cas (20,6 %) et 21 cas (16 %). Nous avions enregistré 37 perdus de vue (20,6 %), 8 transferts (6,1 %) et 4 décès (3,1 %).

## Conclusion

Dans notre cohorte, l’infection à VIH touche particulièrement les jeunes HSH célibataires. Une charge virale indétectable était obtenue chez la majorité des HSH. L’évolution était marquée par un taux élevé de perdus de vue qui constitue un frein à la prise en charge.

# Research article: 234 - Évaluation du programme d’élimination de la transmission mère-enfant du VIH au Sénégal à travers les résultats de diagnostic précoce des enfants exposés

**Published:** 2024-02-23

**Authors:** Ndeye Marame DIONE, Khadidia FALL, Diakhou MBAYE, Karim DIOP, Kouro BOUSSO, Babacar FAYE, Halimatou DIOP-NDIAYE, Abdoul Mazid DIONE, Ndeye Fatou NGOM, Cheikh Tidiane NDOUR

**Affiliations:** 1 Division de lutte contre le sida et les IST (DLSI), Ministère de la santé, Sénégal; 2 Conseil national de lutte contre le sida (CNLS), Sénégal; 3 Laboratoire de bactériologie et de virologie (LBV), Centre hospitalier national universitaire Aristide Le Dantec (HALD), Dakar, Sénégal; 4 Laboratoire de biologie médicale, Hôpital militaire de Ouakam, Dakar; 5 UNICEF

## Contexte

Le programme de prévention de la transmission mère-enfant du VIH (PTME) du Sénégal, a pris de l’ampleur en 2005 couplé à partir de 2007 à l’utilisation des tests rapides au niveau des postes de santé, à l’adoption de l’option B+, diagnostic précoce des enfants nés de mère séropositives par la méthode de prélèvement sur papier buvard fin 2011, et à la décentralisation du diagnostic précoce par les *points of care* (points de soin) en 2018.

## Objectifs

Évaluer le programme d’élimination de la transmission mère-enfant du VIH au Sénégal.

## Méthodologie

Il s’agit d’une étude rétrospective effectuée sur les enfants nés de mères séropositives du 1^er^ janvier 2016 au 31 décembre 2021 et âgés de 0 à 52 semaines dans tous les sites PTME du pays. Les enfants nés de mères séropositives sont constitués de trois groupes de couples mère-enfant entièrement suivis avec PTME complète, couples mère-enfant n’ayant pas bénéficié de traitement complet ou de prophylaxie et couples mère-enfant n’ayant pas du tout bénéficié de traitement ou de prophylaxie. Les données de ces enfants obtenues *via* le diagnostic précoce ont été analysées.

## Résultats

Au total, 4170 prélèvements ont été collectés chez des enfants exposés avec un sex-ratio de 1,01 et un âge médian de 6 semaines [0-52]. Pour rechercher le VIH, tous les prélèvements ont été testés. Le traitement par la trithérapie ARV chez les mères a été noté dans 96,7 % des cas (n = 4034) et la prophylaxie ARV chez les enfants dans 83 % (n = 3480).

Le VIH a été détecté dans 181 cas, soit une prévalence de 4 % [IC95% : 2,1-4,4 %]. Avec une prévalence de 2 % chez les enfants entièrement pris en charge dans le cadre de la PTME complète (couples mère-enfant ayant bénéficié de traitement et l’enfant mis sous allaitement maternel protégé), 11 % chez les enfants PTME incomplète (couples mèreenfant n’ayant pas bénéficié de traitement complet ou de prophylaxie; mère qui est dépistée en fin de grossesse ou au moment de l’accouchement et/ou enfant n’ayant pas bénéficié de prophylaxie). Par contre le taux d’infection au VIH est 18 fois plus élevé (36 %) chez les enfants hors PTME (couples mère-enfant n’ayant pas bénéficié de traitement ou de prophylaxie avec allaitement maternel exclusif seul).

## Conclusion

Cette étude a montré un taux élevé de VIH chez les enfants sans prévention antirétrovirale, mettant en évidence la nécessité de dépister toutes les femmes enceintes pour le VIH et de promouvoir le traitement par la trithérapie ARV et la prophylaxie des ARV chez les enfants. Par contre, la prévalence plus faible chez ceux sous prévention prouve l’efficacité des ARV dans la réduction de la transmission verticale du VIH.

# Research article: 37 - Profil épidémio-clinique et évolutif de l’herpès génital chez l’adulte à Dakar

**Published:** 2024-02-23

**Authors:** Assane DIOP, Saraye OSMAN, Mame Tene NDIAYE, Biram SECK, Saer DIADIE, Niar NDOUR, Boubabar Ahi DIATTA, Maodo NDIAYE, Moussa SEYDI, Fatimata LY, Suzanne Oumou NIANG

**Affiliations:** 1 Dermatologie, Hôpital Institut d’hygiène sociale, Dakar, Sénégal; 3 Dermatologie, Centre hospitalier national d’enfants Albert Royer, Dakar; 4 Dermatologie, Centre hospitalier national universitaire Aristide Le Dantec, Dakar; 5 Service des maladies infectieuses et tropicales, Centre hospitalier national universitaire de Fann, Dakar

## Introduction

L’herpès génital est la première cause d’ulcérations génitales dans le monde. En Afrique de l’Ouest, les études sur l’herpès génital sont rares. Notre objectif était de déterminer les aspects épidémiocliniques et évolutifs de l’herpès génital à Dakar.

## Méthodes

Il s’agit d’une étude transversale prospective, réalisée sur la période du 15 mai au 15 décembre 2019. Elle était effectuée dans les deux services de référence de Dermatologie de Dakar et aux Maladies infectieuses de Fann. Était inclus tout patient âgé de plus de 18 ans présentant un herpès génital. Le diagnostic était clinique et paraclinique. L’analyse était effectuée grâce au logiciel IBM SPSS 24.

## Résultats

Nous avions recensé 38 patients, soit 44,2 % des cas d’ulcération génitale. L’âge moyen était de 39,6 ans et le sex-ratio de 0,40. La tranche d’âge de 30 à 47 ans représentait 50 % (n = 19) des patients. 15,8 % (n = 6) venaient de l’intérieur du pays et 5,2 % (n = 2) de l’étranger. Les mariés représentaient 44,7 % (n = 17). 66 % (n = 25) étaient non instruits, les professionnelles du sexe représentaient 26 % (n = 7) des femmes, et un patient était un homme ayant des relations sexuelles avec d’autres hommes. L’interrogatoire était incomplet chez 4 patients qui présentaient une méningo-encéphalite d’allure herpétique. L’activité sexuelle était débutée avant 16 ans chez 61,76 % (n = 21). Le rapport sexuel était génital chez 61,76 % (n = 21), oro-génital chez 20,6 % (n = 7) et ano-génital chez 8,8 % (n = 3). Un multi-partenariat était noté chez 44 % (n = 15) et l’utilisation du préservatif n’était systématique que chez 1 patient. 41,2 % (n = 14) rapportaient des ulcérations génitales à répétition. Il existait une infection à VIH chez 44,7 % (n = 17) et un diabète chez 21 % (n = 8). On notait une douleur à type de brûlure chez tous et un prurit chez 81,6 %. Les lésions étaient à type d’ulcérations (78,94 %), d’érosions (15,78 %) et de vésicules (5,28 %). L’atteinte extra-génitale était objectivée chez 57,9 % (n = 22) et les adénopathies inguinales chez 39,47 % (n = 15). Des condylomes étaient notés chez 8 % (n = 3). La PCR objectivait HSV-2 chez 3 patients sur les 6 qui l’avaient effectuée. L’histopathologie était contributive chez 2 patients qui présentaient une forme ulcéro-bourgeonnante. La sérologie syphilitique et l’AgHbs étaient négatifs chez tous. Tous les patients étaient sous aciclovir ou valaciclovir par voie générale. La guérison était complète à J28 chez 94,7 % (n = 36), 2 décès par méningo-encéphalite sur infection à HIV étaient notés. L’atteinte extra-génitale (p = 0,00008) et le retard de guérison (p = 0,00006) étaient liés à l’infection à VIH.

## Conclusion

L’herpès génital est la première cause d’ulcération génitale. Le multi-partenariat et l’absence de port de préservatifs sont les principaux facteurs de risque. En outre, il constitue un co-facteur important de l’infection à VIH qui est son principal facteur aggravant.

# Research article: 194 - Expérience de la PTME en milieu de travail : exemple de la Compagnie sucrière sénégalaise

**Published:** 2024-02-23

**Authors:** Boubacar SANÉ, Aboubacar GASSAMA, Moussa DIAGANA, Abdou A. NIANG, Modou Mbacké GUEYE, Souleymane NIANG, Boury DIOP

**Affiliations:** Compagnie sucrière sénégalaise (CSS)

## Introduction

La mise en place d’une stratégie de prévention de la transmission mèreenfant (PTME) pour les femmes séropositives devient une nécessité du fait de l’augmentation des agents féminins, de la mise en place d’une consultation prénatale dans l’entreprise et du fait que la CSS constitue le 3^e^ site dans la prise en charge des PvVIH. Cette stratégie participera à la réduction de la transmission mère-enfant.

## Méthodologie

L’étude porte sur les dossiers de consultation prénatale et des sujets vivant avec le VIH de 2015 à 2022.

La sage-femme dispose des tests de diagnostic rapide pour le VIH avec une confirmation au niveau du laboratoire.

Nous avons suivi 19 femmes enceintes séropositives avec 23 grossesses dont la moyenne d’âge était de 38 ans, la plus âgée ayant 45 ans et la plus jeune 31 ans, toutes ces femmes étant sous trithérapie – AZT + 3TC + NEV / TDF + 3TC + EFV / TDF + 3TC + DTG selon les recommandations en vigueur – et toutes les femmes ayant choisi l’allaitement maternel protégé et étant du profil 1.

Les 19 femmes ont bénéficié d’une charge virale durant le dernier tiers de la grossesse. Un suivi lors des consultations prénatales a été instauré avec un accent particulier lors du dernier tiers.

Un plan d’accouchement était mis en place dès la 37^e^ semaine d’aménorrhée sous la supervision de la sage-femme en collaboration avec l’équipe de prise en charge et de la patiente.

Le site d’accouchement déjà identifié pouvait être soit l’EPS 1 de Richard-Toll, le Centre de santé ou les postes les plus proches.

Des dispositions ont été prises pour assurer la continuité des ARV chez la maman et la forme pédiatrique pour l’enfant toujours sous la supervision de la sage-femme.

Tous les 23 nouveau-nés ont bénéficié d’un prélèvement PCR1 à 6 semaines et les résultats sont revenus avant la date de la 2^e^ PCR. Le processus de sevrage à M12 a été mis en place et la sérologie définitive effectuée à M14.

## Résultats

Sur les 23 PCR1 effectuées, 20 sont revenues négatives, 1 cas positif et 2 cas sont en attente.

Sur 21 nouveau-nés, 20 ont eu une sérologie négative à M14 après sevrage et 1 cas de sérologie positive concerne le cas de la PCR1 qui était positive au départ, qui a néanmoins bénéficié d’un traitement.

Quatre femmes ont eu chacune 2 grossesses successives avec charge virale indétectable et ont donné naissance à un premier et un deuxième enfant tous négatifs.

Sur les 19 femmes suivies, 18 ont eu une charge virale indétectable, pour 1 la charge virale est supérieure à 1000 copies/ml et la mère de l’enfant est positive.

## Conclusion

Malgré la charge de travail dans l’entreprise, la sage-femme avec l’appui de l’équipe de prise en charge s’attelle à préserver la santé des femmes séropositives et à participer à l’élimination de la transmission mère-enfant du VIH.

# Research article: 109 - Vieillir en bonne santé avec le VIH : caractéristiques de la population âgée sous traitement antirétroviral au Cameroun et au Sénégal et données sur les facteurs de risque cardiovasculaires

**Published:** 2024-02-23

**Authors:** El Hadji Bara DIOP, Laura CIAFFI, Modibo SAIDOU, Gabrièle LABORDE-BALEN, Madjiguène GUEYE, Samuel Birama SY, Ndeye Fatou NGOM GUEYE, Kiné NDIAYE, Karim DIOP, Babacar FAYE, Bernard TAVERNE, Serge Clotaire BILLONG, Mamadou COUME

**Affiliations:** 1 Centre régional de recherche et de formation à la prise en charge clinique de Fann (CRCF), Dakar, Sénégal; 2 Projet VIHeillir, Cameroun et Sénégal; 3 Comité national de lutte contre le sida (CNLS), Yaoundé, Cameroun; 4 TransVIHMI, Université de Montpellier, INSERM, IRD, Montpellier, France; 5 Centre de traitement ambulatoire, Centre hospitalier national universitaire de Fann, Dakar, Sénégal; 6 Hôpital militaire de Ouakam, Dakar, Sénégal; 7 Service gériatrie, Centre hospitalier national universitaire de Fann, Dakar, Sénégal; 8 Faculté de Médecine et des sciences biomédicales, Université de Yaoundé I, Cameroun

## Objectifs

L’accès élargi aux antirétroviraux (ARV) a transformé l’infection à VIH en une maladie chronique avec un vieillissement progressif de la population sous ARV. Au Cameroun comme au Sénégal, les personnes âgées de plus de 50 ans vivant avec le VIH (PAvVIH) représentent 24 % des files actives (premier semestre 2021). Le projet VIHeillir financé par Expertise France a pour but d’intégrer la prise en charge des comorbidités chez les PAvVIH dans 5 centres pilotes (2 au Cameroun et 3 au Sénégal). Cette analyse visait à présenter les caractéristiques sociodémographiques et médicales des PAvVIH sous traitement ARV et les données sur les facteurs de risque cardiovasculaires.

## Matériel et méthodes

Après standardisation et simplification des protocoles de prise en charge avec les experts et les autorités de chaque pays, le personnel des centres impliqués a été formé et le matériel nécessaire au dépistage de l’hypertension artérielle (HTA) et du diabète a été mis à disposition des équipes. La disponibilité de médicaments à coût réduit a été assurée par l’accès aux initiatives nationales déjà prévues par les États. Chaque PAvVIH se présentant en consultation pouvait accéder au programme VIHeillir en signant un consentement à l’utilisation des données. Ensuite, des informations démographiques, sur la qualité de vie et les facteurs de risque cardiovasculaires sont collectées (RedCap). Plusieurs mesures de la pression artérielle et une glycémie mesurée par glucomètre ou au laboratoire assurent le dépistage de HTA (gratuit) et du diabète (1000 CFA restant à la charge du patient).

## Résultats

Entre juin 2021 et août 2022, 1452 PAvVIH ont été enregistrées, dont 1098 au Cameroun et 354 au Sénégal. Nous observons une prédominance féminine (66,6 %, n = 967) et l’âge moyen est de 59 ans (50-84 ans). La durée médiane de l’infection au VIH est de 12 années. S’agissant des facteurs de risque des maladies cardiovasculaires et métaboliques : 21 % (305/1452) des patients ont des antécédents familiaux de maladies cardiovasculaires, 16 % (233/1452) sont obèses, 67 % (973/1452) ne pratiquent aucune activité physique, 6 % (87/1452) sont tabagiques et 30 % (305/635) consomment de l’alcool. Nous observons une valeur tensionnelle supérieure à la normale chez 40 % (436/1452) des patients, ceux qui ont un diagnostic confirmé d’HTA étant au nombre 378 (26 %). La glycémie à jeun avec des valeurs supérieures à 1,26 g/L a été retrouvée chez 279 personnes, soit 19 % des cas. Le diagnostic a été confirmé chez 116 patients (8 %).

## Conclusions

L’analyse préliminaire du profil clinique et biologique des PAvVIH révèle une prévalence élevée des facteurs de risque cardiovasculaires, notamment l’HTA, le diabète de type 2 et l’obésité. Toutefois, l’accès au diagnostic et au traitement est limité. Pour conserver les acquis de la réduction de la morbi-mortalité chez les PAvVIH, l’accès à la prévention et aux soins des maladies métaboliques et cardiovasculaires est indispensable.

# Research article: 5 - Changement des profils génétiques des souches de VIH-1 au Sénégal

**Published:** 2024-02-23

**Authors:** Abou Abdallah Malick DIOUARA, Aissatou Ahmet NIANG, Mohammadou Lamine DIA, Fatoumata DIALLO, Papa Adama MBOUP, Mengué FALL, Adjratou Aissatou BA, Ndeye Coumba TOURÉ, Abdoulaye Banire DIALLO, Halimatou DIOPNDIAYE

**Affiliations:** 1 Laboratoire de bactériologie et de virologie, Centre hospitalier national universitaire de Fann, Université Cheikh Anta Diop, Dakar, Sénégal; 2 Groupe de recherche Biotechnologies appliquées & Bioprocédés environnementaux (GRBA-BE), École supérieure polytechnique (ESP), Université Cheikh Anta Diop, Dakar; 3 Laboratoire de bactériologie et de virologie, Centre hospitalier national universitaire Aristide Le Dantec, Université Cheikh Anta Diop, Dakar; 4 Laboratoire Bioinformatique, Université du Québec à Montréal, Montréal, Canada

## Objectif

L’épidémie de VIH-1 au Sénégal est caractérisée par une grande diversité génétique des souches circulantes avec la co-circulation de plusieurs sous-types et formes recombinantes. Ce travail avait pour objectifs de déterminer l’évolution de la diversité génétique des séquences des gènes gag et env de VIH-1 au Sénégal depuis le début de l’épidémie.

## Matériels et méthodes

L’étude a porté sur l’ensemble des séquences des gènes gag [790-1921] et env [2353-3554] du VIH-1 disponibles en ligne. La taille minimale des séquences était fixée à 500 nucléotides pour être éligibles. Le filtrage a été fait avec un script python et les séquences ont été sauvegardées dans une base de données (Laragon). Les données recueillies ont été stratifiées en intervalles de 5 ans sur toute la période de la collecte. Pour le gène env, tous les codons correspondant aux positions connues de mutations de résistance aux antirétroviraux ont été supprimés dans l’alignement des séquences qui a été effectué par le logiciel MUSCLE. Pour affiner les alignements de séquences, les positions avec les gaps ont été supprimées à l’aide du programme « Gblocks » du logiciel SeaView. L’arbre phylogénétique a été réalisé à l’aide du logiciel SeaView et les séquences représentant plus de 5 % de l’effectif ont été considérées pour l’analyse de l’évolution.

## Résultats

De 1990 à 2014, 286 et 172 séquences des gènes gag et env ont été respectivement collectées. Après filtrage, 281 séquences de gag ont été retenues avec 17 variants et une prédominance du CRF02_ AG (51 %), suivi de C (16 %), B (10 %) et A3 (6 %). Pour env, 169 séquences ont été retenues avec 21 variants et une prédominance de CRF02_AG (33 %), suivi de C (23 %), B (10 %) et A1 (9 %).

Ainsi de 1990 à 1994, le nombre de séquences gag et env est respectivement de 5 et 41 avec pour gag, 3 D, 1 C et 1 CRF02_AG et pour env 41 % de CRF02_AG, suivi de A1, C et D. De 1995 à 1999, 181 séquences de gag et 60 de env ont été notées avec respectivement 64 % de CRF02_AG suivis de A3 et B, et 58 % de CRF02_AG suivis de C et D.

Entre 2000 et 2004, seules 2 séquences de gag (A3) et de env (2 A3 et 2 CRF02_AG) ont été obtenues.

De 2005 à 2009, le variant majoritaire était C pour gag (n = 93) et env (n = 60) avec respectivement 37 % et 46 % suivis de CRF02_AG et B.

De 2010 à 2014, 3 séquences gag et env ont été retenues avec 2 CRF02_AGtCRF02_AG/CRF06_cpx.

## Conclusion

Il est noté au fil des années une baisse de la proportion de CRF02_AG au profit du sous-type C qui est plus largement représenté dans le groupe des hommes ayant des relations sexuelles avec d’autres hommes. Ces résultats doivent cependant être confirmés par l’analyse d’autres gènes, en particulier le gène pol.

# Research article: 223 - Perceptions des adaptations du CEPIAD à la Covid-19 par les consommateurs de drogues injectables (CDI)

**Published:** 2024-02-23

**Authors:** Mouhamet DIOP, Alice DESCLAUX, Khoudia SOW

**Affiliations:** 1 Centre régional de recherche et de formation à la prise en charge clinique de Fann (CRCF), Dakar, Sénégal; 2 Université Assane Seck, Ziguinchor, Sénégal; 3 Institut de recherche pour le développement (IRD)

## Objectifs

L’épidémie de Covid-19 a eu des impacts sur le fonctionnement des systèmes de santé qui ont suscité plusieurs réactions et adaptations. Au Centre de prise en charge intégrée des addictions de Dakar (CEPIAD), ouvert depuis 2014 comme projet pilote de Réduction des risques, le personnel a défini un ensemble de dispositions en phase avec les recommandations de l’ONUDC (Office des Nations unies contre la drogue et le crime) pour assurer la continuité des soins et le respect des mesures préventives. L’objectif de cette présentation est de décrire les perceptions des patients à propos des adaptations du CEPIAD à la Covid-19.

## Méthodes

La démarche repose sur des observations et des entretiens individuels semistructurés réalisés auprès de 22 consommateurs de drogues injectables (CDI) recevant un traitement par méthadone dans le centre où nous travaillons en immersion depuis 5 ans. Les entretiens ont été transcrits, nettoyés et anonymisés et les données ont été traitées par une analyse de contenu.

## Résultats

Les CDI recevant de la méthadone ont globalement une bonne connaissance des modes de contamination pour la Covid-19 et estiment que « la maladie n’épargne personne ». Selon eux, la consommation d’héroïne ou de cocaïne augmenterait les risques de transmission car « la Covid affecte les poumons » et sous l’effet de la drogue, ou de l’état de manque, ils n’accorderaient pas d’attention aux mesures barrières. Au CEPIAD, l’application des mesures préventives a imposé une limite de la circulation des patients, le planning des consultations a été redéfini et l’emport à domicile de la méthadone a été généralisé à tous les patients pour éviter leurs déplacements et la promiscuité dans les transports en commun. Les adaptations du CEPIAD sont appréciées par la majorité des patients, notamment l’emport généralisé de la méthadone. Les CDI perçoivent la fréquentation du centre comme un facteur de protection car ils y sont obligés de respecter les recommandations contrôlées par les pairs médiateurs et par un vigile. Cependant, l’interruption de certaines activités et la suspension de la libre circulation des patients dans le centre limitent la socialisation informelle, ce que regrettent certains CDI. Ils apprécient globalement le fait qu’aucun cas de Covid-19 n’ait été enregistré au CEPIAD.

## Conclusion

Les résultats montrent que les mesures préventives de la Covid-19 ont été bien appliquées dans une population prise en charge pour des addictions. Les perceptions des patients mettent en évidence les acquis du programme de Réduction des risques qui a installé une démarche d’hygiène et de gestion des risques dans cette population.

# Research article: 70 - Décentralisation du test Xpert HPV pour le diagnostic précoce des lésions précancéreuses du col de l’utérus au Sénégal : résultats de l’étude pilote au niveau de quatre districts sanitaires

**Published:** 2024-02-23

**Authors:** Oumy DIOP, Assane DIENG, Yacine DIENG

**Affiliations:** 1 Laboratoire de bactériologie et de virologie, Centre hospitalier national universitaire Aristide Le Dantec, Dakar, Sénégal; 2 Division de lutte contre les maladies non transmissibles, Ministère de la santé, Dakar

## Introduction

L’infection au papillomavirus humain (PVH), responsable de cancer du col de l’utérus, est un problème majeur de santé publique au Sénégal où le dépistage repose essentiellement sur l’Inspection visuelle à l’acide acétique (IVA) qui selon les recommandations de l’OMS doit être remplacée par les techniques moléculaires. C’est ainsi qu’une étude pilote a été initiée pour évaluer la faisabilité opérationnelle de l’utilisation du test PVH pour le dépistage des lésions précancéreuses au Sénégal.

## Méthodologie

Il s’agit d’une étude pilote réalisée entre septembre et décembre 2020 au niveau de quatre districts sanitaires (Dakar centre, Guédiawaye, Mbour et Fatick) et portant sur toutes les femmes âgées de 30 à 54 ans consentantes.

Un prélèvement au niveau de l’endocol par une sage-femme ou un auto-prélèvement a été réalisé à l’aide respectivement d’une cytobrosse ou d’un écouvillon. Les échantillons conditionnés dans du ThinPreP ont été acheminés dans le laboratoire du site où la détection de l’ADN du PVH a été réalisée avec le test Xpert HPV (Cepheid) qui détecte les PVH-16, -18/45 et 12 autres génotypes à haut risque oncogène. Un prélèvement sanguin a aussi été réalisé pour le dépistage du VIH. Tous les échantillons positifs et un dixième des négatifs ont été acheminés au Laboratoire de bactériologie et de virologie du CHU Le Dantec pour faire l’objet d’un contrôle de qualité. L’analyse des données a été réalisée en utilisant les logiciels RStudio et Excel.

## Résultat

Sur une cible estimée à 1800 femmes, 1580 femmes ont pu bénéficier d’un dépistage du PVH soit un taux d’acceptation de 87,8 %. La médiane d’âge était de 40 ans et celle du premier rapport sexuel était de 21 ans. La grande majorité des femmes dépistées (90 %) étaient mariées et la médiane du nombre de leurs enfants était de 4.

L’ADN du PVH a été retrouvé chez 194 femmes testées (12 %) avec des différences par district allant de 9 % (à Mbour) à 14 % (à Dakar centre). L’analyse des génotypes a retrouvé 26 cas de PVH-16 (12 %), 40 cas de PVH-18/45 (18 %) et 151 autres types (70 %).

Les résultats du contrôle de qualité des tests PVH ont montré des taux de discordance variant de 5,2 % à Dakar centre à 12,3 % à Wakhinane sur la base d’un test PVH positif ou négatif et de 7,5 % à Fatick à 21,1 % à Wakhinane selon le génotype.

Six femmes ont été dépistées positives au VIH soit une prévalence de 0,4 % dont 2 coinfections VIH/PVH positives.

## Conclusion

Les résultats de ce pilote ont montré l’intérêt de l’utilisation du test Xpert HPV dans le dépistage du PVH en routine et ont permis d’identifier les sites nécessitant un accompagnement pour des résultats de qualité.

# Research article: 221 - SEN’RT-Biobanking : vers la mise en place d’un système de gestion en temps réel d’une Biobanque nationale

**Published:** 2024-02-23

**Authors:** Abou Abdallah Malick DIOURA, Idy DIOP, Mame Diarra DIONE, Zinguinzou N’GUISSAN, Sophie Délia TENE, Seynabou COUNDOUL, Sarbanding SANÉ, Fatou THIAM, Alé KANE, Nouhou DIABY, Mame Ndew MBAYE, Coumba TOURÉ KANE

**Affiliations:** 1 Groupe de recherche Biotechnologies appliquées & Bioprocédés environnementaux (GRBA-BE), École supérieure polytechnique (ESP), Université Cheikh Anta Diop, Dakar, Sénégal; 2 Laboratoire d’imagerie médicale et bio-informatique, École supérieure polytechnique (ESP), Université Cheikh Anta Diop (UCAD), Dakar; 3 UFR des Sciences agronomiques, de l’aquaculture et des technologies alimentaires (S2ATA), Université Gaston Berger (UGB), Saint-Louis, Sénégal; 4 Laboratoire de traitement des eaux usées (LATEU), Institut fondamental d’Afrique Noire (IFAN), Université Cheikh Anta Diop, Dakar; 5 Université du Sine Saloum El Hâdj Ibrahima Niass (USSEIN), Sénégal; 6 Institut de recherche en santé, de surveillance épidémiologique et de formation (IRESSEF), Sénégal

## Description du problème

La conservation, le stockage et la gestion des ressources biologiques et données associées constituent des défis multifacettes pour les services de laboratoire d’analyses et de recherche. En effet, la fiabilité des résultats de même que le niveau de satisfaction des usagers résident essentiellement sur des aspects liés à son système de management de la qualité (SMQ). Par ailleurs, l’une des causes d’erreurs de diagnostics biologiques menant à des résultats erronés aux conséquences potentiellement désastreuses, pourrait être liée à un défaut d’intégrité et/ou de bonne conservation des échantillons ou encore une mauvaise tenue des métadonnées.

Dès lors, il est question de tirer profit de la mutualisation des ressources biologiques et de la réutilisation des reliquats de spécimens pour pallier les coûts associés à la collecte de spécimens d’une part et d’autre part les difficultés d’accès aux échantillons cliniques; le tout pour le développement de la recherche. Par conséquent, ce projet vise l’installation d’un système automatisé d’identification et de monitoring en temps réel de ressources biologiques.

## Activités réalisées et résultats obtenus

Depuis la conception de l’architecture de la plateforme en juillet 2020, la mise en œuvre des travaux a impliqué des partenaires nationaux et internationaux. La première phase a donné lieu à un prototype de plateforme « RT-Biobanking » dimensionnée pour des applications en santé. Dans le cadre du programme iLEAD de la fondation Bill & Melinda Gates, ce prototype a été présenté aux différentes parties prenantes y compris de potentiels utilisateurs finaux en juin 2021 à l’IRESSEF de Diamniadio. De cette expérience, nous avons retenu la nécessité d’étendre cette plateforme à l’élevage, à l’agrobusiness et à des applications « One Health » (environnement, santé humaine et animale).

Cette seconde phase du projet bénéficie de l’appui de l’École supérieure polytechnique (ESP) de l’UCAD à travers son programme « Fonds d’impulsion à la recherche ». Le développement de modules spécifiques couvrant ces champs et l’intégration des données sont en cours. À titre d’exemple, au 18 septembre 2022, 4859 échantillons de différentes catégories ont été répertoriés dans la seule Biobanque du GRBA-BE de l’ESP. La répartition des échantillons selon les types de matrices fait état de 3846 spécimens biologiques de types Plasma, Buffy Coat et tissus biopsiques. Le reste de la Biobanque est constitué de produits carnés, de produits horticoles et d’échantillons environnementaux (eaux usées, mixte eaux usées et eaux de pluies, sols). Les lieux de collecte de ces échantillons étaient entre autres Dakar (plus de 15 sites), Saint-Louis, Ziguinchor, Kédougou, Sédhiou, Richard Toll et Kolda.

## Leçons apprises et étapes futures

Les résultats préliminaires obtenus ont montré une facilité d’utilisation du prototype expérimental de la plateforme SEN’RT-Biobanking. Les métadonnées entreposées et les résultats issus de différentes études pourraient faciliter le développement et l’intégration de modules intelligents pour la surveillance et la prédiction d’événements épidémiologiques.

# Research article: 132 - Phase de décentralisation : prophylaxie pré-exposition continue chez les populations clés à l’hôpital régional de Saint-Louis du Sénégal

**Published:** 2024-02-23

**Authors:** Daouda DIOUF, Alassane SARR, Ahmed DIENG, Ndeye Fatou DIEDHIOU, Mame Mbeugué GAYE

**Affiliations:** 1 Centre hospitalier régional de Saint-Louis, Sénégal; 2 EpiC USAID/FHI 360, Sénégal

## Objectif

La prophylaxie pré-exposition (PrEP) est une nouvelle stratégie de prévention du VIH recommandée par l’OMS depuis septembre 2015. Au Sénégal, la PrEP continue a été adoptée et proposée aux populations clés (pop-clés) notamment les hommes ayant des relations sexuelles avec d’autres hommes (HSH). L’objectif de notre travail est de relater les résultats provisoires sur les aspects épidémiologiques, cliniques et immuno-biologiques de la PrEP au sein de la structure.

## Méthodes

C’est une étude rétrospective et descriptive de la PrEP continue chez les popclés au niveau de l’hôpital régional de Saint-Louis sur une durée de 13 mois allant d’août 2021 à septembre 2022. Les données ont été collectées à partir du registre de suivi et des dossiers individuels de patients.

## Résultats obtenus

Au cours de l’étude, nous avons recensé 60 pop-clés qui ont accepté la proposition de la PrEP dont 58 HSH, une femme transgenre et le conjoint d’un couple séro-différent. L’âge moyen des clients était de 25,75 ans avec des extrêmes de 16 et 51 ans. La majeure partie des patients (88,3 %) étaient célibataires et 11,7 % mariés. Le dépistage de l’infection par le VIH est revenu positif chez 5 patients soit 8,3 %. Le dépistage de l’infection par le VHB a été réalisé chez 46 patients soit 76,6 % et un seul test est revenu positif. Le risque élevé d’infection par le VIH était dominé par les rapports vaginaux et anaux sans protection qui étaient présents chez 43 patients (71,66 %) suivis du multi-partenariat présent dans 63,33 % des cas. L’antécédent d’IST n’était présent que dans 5 % des cas. La recherche d’IST à l’inclusion a été réalisée chez 34 personnes soit 55 % et est revenue positive dans 1 cas.

L’ensemble des patients avaient une fonction rénale normale.

Au total, 54 patients (90 %) ont bénéficié de la PrEP et les 10 % restants ont présenté un test positif à l’infection par le HIV ou le VHB. Le traitement utilisé était le Truvada (TDF/FTC). Nous n’avons recensé aucune infection par le VIH au cours du suivi.

## Conclusion

La PrEP est une méthode efficace de prévention de la transmission de l’infection par le VIH. Selon les recommandations de l’OMS, le Sénégal s’est inscrit dans une dynamique de vulgarisation de cette méthode dans le cadre de la décentralisation dans les régions. Sa mise en œuvre, notamment dans notre contexte d’épidémie de type concentrée, va contribuer à la perspective de l’élimination de l’infection par le VIH en 2030.

# Research article: 12 - Facteurs associés au passage en deuxième ligne de traitement et à l’échec thérapeutique chez l’enfant vivant avec le VIH à Guédiawaye, Sénégal

**Published:** 2024-02-23

**Authors:** Jean Baptiste Niokhor DIOUF, Mariama KANE, Aissata DIALLO, Seydina Mouhamad BÂ

**Affiliations:** Centre hospitalier Roi Baudouin, Guédiawaye, Sénégal

## Introduction

L’infection à VIH de l’enfant constitue un problème majeur de santé publique. L’objectif de cette étude réalisée au service de pédiatrie du Centre hospitalier Roi Baudouin de Guédiawaye était d’évaluer les aspects épidémiologiques, cliniques, thérapeutiques et évolutifs d’une cohorte d’enfants atteints de VIH sur une période de 15 ans et de déterminer les facteurs associés à l’arrêt thérapeutique et au passage en deuxième ligne.

## Patients et méthodes

Il s’agissait d’une étude transversale descriptive et analytique sur une période allant d’août 2004 à décembre 2019 au Centre hospitalier Roi Baudouin de Guédiawaye. À travers cette étude nous avons évalué l’âge au moment du diagnostic, l’âge actuel, le sexe, le statut d’orphelin, les aspects thérapeutiques, l’annonce du statut et l’évolution.

## Résultats

Au total, 129 dossiers de patients étaient étudiés sur une période de 15 ans (de 2004 à 2019). Les enfants séropositifs de profil I représentaient 93,8 % et il y avait une prédominance masculine avec un sex-ratio de 1,43. L’âge moyen actuel des enfants était de 12,2 ± 4,5 et la médiane de 14 ans. Les orphelins de père ou de mère représentaient respectivement 24,0 % et 20,2 % tandis que les doubles orphelins représentaient 11,6 %. L’âge moyen au début du traitement ARV était de 7,6 ± 4,3 ans et la médiane de 7,5 ans. L’âge moyen de démarrage de la deuxième ligne était de 13,7 ans avec un écart type de 2,6 et la médiane était de 13 ans. L’association AZT + 3TC + NVP était la plus utilisée (29,5 %) suivie de l’association TDF + FTC + NVP (26,2 %). L’âge moyen au moment de l’arrêt du traitement était de 13,3 ± 4,3 ans et la médiane de 15 ans. Plus de la moitié des enfants (57,0 %, n = 69) étaient informés de leur statut. Le taux de mortalité était de 3,9 %. Les facteurs associés à l’arrêt thérapeutique étaient le sexe (p value = 0,025 et OR = 3,2), le statut d’orphelin (p value = 0,027 et OR = 3,0) et la durée de suivi supérieure à 10 ans (p value = 0,013 et OR = 5,6). Les facteurs associés au passage en deuxième ligne étaient l’année d’inclusion (p value = 0,001 et OR = 15,6), la tranche d’âge (p value = 0,001 et OR = 19,2), le statut d’orphelin (p value = 0,040 et OR = 2,6), le régime thérapeutique (p value = 0,019 et OR = 5,7), la durée de la prise ARV (p value = 0,000 et OR = 38,0) et l’annonce (p value = 0,002 et OR = 4,7).

## Conclusion

La cohorte d’enfants infectés par le VIH et suivis à Guédiawaye reste dominée par les adolescents. Le statut d’orphelin, l’annonce du statut et la durée de suivi sont associés au passage en seconde ligne et à l’arrêt du traitement.

# Research article: 135 - Connaissances, attitudes et pratiques sur l’hépatite virale B du personnel de santé du Centre hospitalier national universitaire de Fann

**Published:** 2024-02-23

**Authors:** Papa Latyr Junior DIOUF

**Affiliations:** Service des maladies infectieuses et tropicales, Centre hospitalier national universitaire de Fann, Dakar, Sénégal

## Introduction

Le personnel de santé constitue l’un des groupes les plus à risque d’être affectés par l’hépatite virale B, particulièrement dans nos pays à ressources limitées. C’est dans ce contexte que nous avons mené cette étude dont les objectifs étaient de déterminer les connaissances, attitudes et pratiques du personnel de santé du CHNU de Fann sur l’hépatite virale B et d’identifier les facteurs associés.

## Méthodologie

Il s’agit d’une étude transversale, descriptive et à visée analytique réalisée à partir de questionnaires auto-administrés sur la période du 1^er^ mars au 10 mai 2021.

## Résultats

Cent cinquante-cinq fiches d’enquête ont été analysées. L’âge moyen du personnel était de 32 ans (± 8 ans). On notait une prédominance masculine (82/155) avec un sex-ratio de 1,12. Les médecins (45 %) étaient la catégorie professionnelle la plus représentée. Près de la moitié (51 %) des enquêtés avaient moins de 5 ans d’ancienneté. Cent trente-huit agents (89 %) avaient des connaissances sur l’hépatite virale B, principalement à travers la formation académique (80,4 %), les médias (34,8 %) et la discussion entre collègues (1,4 %). Selon eux, les groupes les plus affectés par l’hépatite B au Sénégal sont les professionnels du sexe (47 %), suivis des professionnels de santé (41,9 %) et des hommes ayant des rapports sexuels avec les hommes (41,3 %). Les principales voies de transmission étaient parentérale (85,2 %), sexuelle (80 %) et materno-fœtale (56,8 %). L’accident avec exposition au sang (86 %), le multi-partenariat sexuel (85 %) et la toxicomanie intraveineuse (77 %) étaient les facteurs de risque les plus fréquemment cités. Les complications de l’hépatite B les plus connues étaient la cirrhose (92 %), le cancer du foie (91 %) et l’insuffisance hépatocellulaire (75 %). Le principal facteur de risque de progression vers le cancer du foie était l’alcool (72 %). Quatre-vingt-huit agents (56,8 %) commençaient le traitement antiviral sur la base d’une atteinte hépatique et des critères biologiques. Les principales molécules efficaces sur le virus de l’hépatite B (VHB) étaient le ténofovir (51,6 %), l’interféron (40 %) et la lamivudine (26,4 %). Quatre-vingt-douze agents (59 %) référaient les patients à un confrère spécialisé devant un test positif au VHB. Les facteurs associés aux bonnes connaissances étaient : le service (p = 0,05), l’antécédent personnel de dépistage (p = 0,05), le nombre de sources de connaissances (p = 0,047), l’antécédent personnel de vaccination (p = 0,04) et la proposition d’une vaccination (p = 0,05). Les facteurs associés aux bonnes pratiques et attitudes étaient : l’âge (p = 0,01), la catégorie professionnelle (p = 0,045), l’ancienneté professionnelle (p = 0,045), l’antécédent personnel de vaccination (p = 0,025), la connaissance de l’existence d’un traitement efficace (p = 0,015) et le nombre de sources de connaissances (p = 0,047).

## Conclusion

Cette étude vient montrer les gaps à combler sur les connaissances, attitudes et pratiques du personnel du CHNU de Fann sur l’hépatite virale B pour une meilleure prise en charge des cas.

# Research article: 188 - Hétérogénéité du variant Omicron au Sénégal

**Published:** 2024-02-23

**Authors:** Cyrille Kouligueul DIÉDHIOU, Abdou PADANE, Khadim GUEYE, Samba NDLOUR, Aminata MBOUP, Nafissatou MBOW, Papa Alassane DIAW, Badara CISSÉ, Makhtar CAMARA, Ambroise AHOUIDI, Ndèye Coumba TOURÉ KANE, Souleymane MBOUP

**Affiliations:** 1 Institut de recherche en santé, de surveillance épidémiologique et de formation (IRESSEF), Dakar, Sénégal; 2 Institut hospitalo-universitaire Méditerranée Infection, Marseille, France; 3 UMR Vitrome, Campus international IRD-UCAD de Hann, Dakar, Sénégal

## Objectif

Depuis l’apparition de la première infection à Covid-19 en décembre 2019 à Wuhan, en Chine, différents variants du Coronavirus-2 du Syndrome respiratoire aigu sévère (SRAS-CoV-2) ont été identifiés dans le monde. Ainsi, un autre variant préoccupant (VOC) a été désigné Omicron (B.1.1.529) le 26 novembre 2021. Au Sénégal, la première souche Omicron a été séquencée à l’IRESSEF le 3 décembre 2021 et a coïncidé avec la forte augmentation des nouvelles infections, annonçant le début de la 4^e^ vague. Ce variant continue de présenter une grande variabilité génétique, malgré la diminution de l’incidence de la Covid-19 au Sénégal. Dans cette étude, nous analysons l’évolution génomique des lignées du variant Omicron détectées au Sénégal et leurs informations phylogénétiques.

## Méthodes

Mille cinquante (1050) écouvillons nasopharyngés provenant de patients positifs au SRAS-CoV-2 par RT-PCR ont été sélectionnés pour le séquençage de juin 2020 à avril 2022. Ces échantillons ont été séquencés à l’aide du protocole nCoV-2019 ARTIC V3 en utilisant l’instrument GridION (Oxford Nanopore Technology). Le génome a été assemblé à l’aide du flux de travail bio-informatique du pipeline ARTIC. Les sites web Nexclade et Pangolin ont été respectivement utilisés pour déterminer le clade et la lignée de chaque séquence génomique. L’analyse des mutations a été effectuée et confirmée en utilisant CoVsurver dans GISAID.

## Résultats obtenus

Tous les échantillons ont été séquencés avec succès. Ainsi, l’analyse génomique a montré que 337 échantillons appartenaient aux lignées du variant Omicron, dont 17,5 % de BA.1 (184/1050), 1,9 % de BA.1.1 (20/1050), 0,19 % de BA.1.17 (2/1050), 11,6 % de BA.2 (122/1050), 0,09 % de BA.2.3 (1/1050), 0,57 % de BA.3 (6/1050), 0,09 % de XE (1/1050), 0,09 % de XN (1/1050) et 213 appartenaient à d’autres variants en majorité Delta 20,28 % (213/1050). Les variants nommés XE et XN sont des lignées recombinantes de BA.1 et BA.2. Cependant, nous avons constaté une grande diversité de ce variant, malgré la diminution de l’incidence de la Covid-19 au Sénégal depuis mi-janvier 2022. Ainsi, BA.1 était la lignée prédominante de décembre à début janvier 2022, provoquant la poussée initiale d’Omicron et la 4^e^ vague au Sénégal. Par la suite, la fréquence de BA.2 a fortement augmenté en janvier 2022 et a remplacé les BA.1 à la mijanvier 2022. Cependant, la fréquence de BA.2 a continué d’augmenter jusqu’en février 2022, alors que le nombre de cas confirmés a commencé à diminuer en mi-janvier 2022 au Sénégal.

En outre, l’analyse phylogénétique des variants détectés à l’IRESSEF a montré que le variant Omicron n’a pas évolué à partir du premier VOC puisque son génome est différent de celui d’Alpha ou Delta.

## Conclusion

Une diversité significative du variant Omicron a été observée, malgré une diminution de l’incidence de la Covid-19 au Sénégal. En raison de la circulation du SRAS-CoV-2 dans la population générale, qui contribue à accroître les possibilités de recombinaison entre des variants génétiquement distincts, il est impératif de poursuivre et de renforcer la surveillance génomique à l’échelle mondiale et d’évaluer rapidement les caractéristiques biologiques des variants émergents potentiellement préoccupants qui pourraient être à l’origine de nouvelles vagues épidémiques.

# Research article: 153 - Étude de deux algorithmes de dépistage et de traitement du cancer du col de l’utérus (CCU)

**Published:** 2024-02-23

**Authors:** Aboubacry DRAMÉ, Birama DEMBELE, Awa Ba DIALLO, Fatma GUENOUNE, Amadou YOUM, Madeleine MBOW, Oumy DIONGUE, Mansour NIANG, Mame Yacine FALL, Mamadou SIDIBÉ, Halimatou DIOP-NDIAYE, Mamadou DIOP

**Affiliations:** 1 Agence nationale de la statistique et de la démographie (ANSD), Sénégal; 2 Institut Joliot-Curie du cancer, Centre hospitalier national universitaire Aristide Le Dantec, Dakar, Sénégal; 3 Laboratoire de bactériologie et de virologie (LBV), Centre hospitalier national universitaire Aristide Le Dantec, Dakar; 4 Ligue sénégalaise contre le cancer (LISCA)

## Introduction

Les papillomavirus humains (PVH) sont les agents étiologiques des cancers du col de l’utérus (CCU). La persistance des PVH à haut risque est le plus grand facteur de risque de progression de lésions précancéreuses et cancéreuses. L’ablation thermique est le traitement recommandé des néoplasies intra-épithéliales cervicales de haut grade (CIN) diagnostiquées histologiquement, afin de prévenir la progression vers le cancer du col de l’utérus. Ainsi, une étude pilote sur les algorithmes de dépistage et de traitement du CCU a été réalisée au Sénégal chez les femmes séronégatives au VIH dans le département de Guédiawaye. L’objectif de cette étude était de comparer l’efficacité des algorithmes de dépistage du cancer du col de l’utérus par le test PVH + triage IVA + traitement ou par le test PVH + traitement.

## Méthodologie

L’étude consistait à recruter des femmes séronégatives au VIH à qui un questionnaire sur les caractéristiques démographiques, reproductives et socio-économique a été soumis. Elles ont été aussi invitées à prélever elles-mêmes un échantillon vaginal et ensuite, une infirmière a réalisé un autre prélèvement au niveau du col de l’utérus. Les deux échantillons ont été utilisés pour le test PVH. Les femmes positives au PVH ont été randomisées en deux bras selon le ratio 3:1. Dans le bras 1, les femmes positives au PVH ont été testées aussi à l’inspection visuelle à l’acide acétique (IVA) et le traitement n’a été effectué que si l’IVA était positive. Dans le bras 2, les femmes positives au PVH ont été directement traitées par ablation thermique.

Toutes les femmes positives au PVH, traitées ou non, ont été rappelées à 12 mois pour une visite de suivi comprenant un test PVH, une IVA, une colposcopie et une biopsie.

## Résultats

Sur les 350 femmes recrutées, 55 ont été positives au PVH par auto-prélèvement et 40 par prélèvement d’une spécialiste. Au total, 63 femmes étaient positives au PVH et réparties dans les deux bras avec 48 femmes dans le bras 1 (30 IVA-, 18 IVA+ et une perdue de vue) et 15 femmes dans le bras 2 (avec 1 perdue de vue et 1 décès). Après 12 mois, 60 femmes ont bénéficié des tests finaux pour comparer les deux algorithmes. Les résultats ont montré que la clairance était de 61,7 % (19/47) pour le bras 1 contre 84,6 % (11/13) pour le bras 2. Cette clairance était de 53,3 % (16/30) pour les non-traitées du bras 1 et 76,5 % (13/17) pour les traitées de ce même bras. La persistance du PVH était de 33,3 % (20/60) qui reste élevée chez les femmes non traitées (43,8 %).

## Conclusion

Le traitement par ablation thermique reste le meilleur moyen de prévention contre les lésions précancéreuses et cancéreuses. Mais son application est efficace lorsqu’elle est effectuée dès que la femme est positive au PVH sans tri IVA.

# Research article: 257 - La prophylaxie pré-exposition communautaire au Sénégal face aux barrières socioculturelles : les femmes au foyer sous la contrainte d’une clandestinité aux allures sociales et sanitaires

**Published:** 2024-02-23

**Authors:** Barra Lamine FALL, Maguatte MBODJ, Magath POUYE ANCS

**Affiliations:** (Alliance nationale des communautés pour la santé), Dakar, Sénégal

## Objectifs

Le Sénégal vient d’introduire la prophylaxie pré-exposition (PrEP) au niveau communautaire public, qui connaît un gap au cours du 1^er^ semestre 2021 : 48 % chez les hommes ayant des relations sexuelles avec d’autres hommes (HSH) et 9 % chez les professionnelles du sexe (PS) au niveau communautaire. Une campagne d’accélération et de rattrapage des indicateurs PrEP a été organisée. En tant qu’anthropologues, nous nous sommes rendus sur le terrain pour comprendre les déterminants qui expliquent cette faiblesse de résultats chez les PS. Comme objectifs, il a été question de :

- mobiliser les acteurs communautaires sur site autour d’une campagne d’accélération et de rattrapage des indicateurs;
- atteindre au moins 80 % chez les PS et 90 % chez les HSH;
- comprendre les déterminants qui expliquent le gap chez les PS.

## Matériels et méthodes

- Organiser une réunion d’orientation sur site autour de la stratégie PrEP.
- Identifier les bénéficiaires à travers des entretiens individuels et des entretiens de groupe avec prise de rendez-vous.
- Orienter vers le labo pour prélèvement et bilan initial. Faire réaliser des prélèvements communautaires par les médiateurs comme alternative et les acheminer pour une manipulation.
- Administrer un questionnaire pour mesurer l’admissibilité et recueillir les données sur l’acceptation et la perception.
- Mettre sous PrEP, collecter, valider les données analysées et tenir une réunion d’évaluation.

## Résultats

Au terme de la campagne, l’indicateur des HSH a atteint 94 % et celui des PS 77,78 %, témoignant d’une grande réussite. Mais derrière cette belle performance se cachent plusieurs réalités :

- Les bénéficiaires n’acceptent pas qu’un individu « sain » (statut VIH négatif surtout) prenne des médicaments. Dans cette société, ils sont réservés aux malades ou aux personnes qui souffrent de troubles passagers.
- Plus de 80 % des PS sont mariées et vivent dans des unités domestiques où cohabitent plusieurs ménage, 70 % d’entre elles sont dans un ménage polygame bien qu’elles pratiquent la prostitution dans la clandestinité. Elles ne savent pas comment conserver les médicaments dont la boîte est lisible et montre facilement qu’il s’agit de Truvada.
- 78 % d’entre elles n’acceptent pas que leur nom soit consigné dans des registres et dossiers individuels car cela reviendrait à figurer sur le registre sanitaire en tant que PS officielle.
- 98 % d’entre elles pensent déjà aux contraintes liées au suivi car l’État du Sénégal a opté pour la PrEP continue et ces femmes veulent décider de rompre et revenir autant qu’elles le voudront.

## Conclusion

La campagne, que l’on pourrait presque qualifier d’étude, a permis de combler le gap en termes d’indicateurs, mais aussi de comprendre les déterminants sanitaires et sociaux.

Elle inspire les recommandations suivantes :

- sensibiliser avec des outils de communication pour faire comprendre que la PrEP est une autre option de prévention et non un traitement;
- mettre en place un dispositif moins administratif pour contourner la perception portée sur les registres et dossiers individuels;
- offrir les médicaments sans les boîtes d’origine pour banaliser et faciliter la conservation;
- plaider auprès de l’État pour l’adoption de la PrEP continue comme discontinue.

# Research article: 205 - Evaluation de l’élimination de la transmission mère-enfant du VIH dans le district sanitaire de Kaolack (Sénégal) : bilan de 5 ans de mise en œuvre (2017-2021)

**Published:** 2024-02-23

**Authors:** Cécile FAYE, Niene SECK, Lucie BASSE, Aminata THIAM, Selly BA, Jules Bashi BAGENDABANGA

**Affiliations:** 1 District sanitaire de Kaolack, Ministère de la santé et de l’action sociale, Sénégal; 2 EpiC USAID/FHI 360, Sénégal

## Objectif

La transmission mère-enfant du VIH est responsable de plus de 90 % des infections de l’enfant. Le Sénégal s’était engagé à atteindre l’élimination de cette transmission fin 2020. C’est dans ce cadre qu’un plan d’accélération a été mis en œuvre dans le district sanitaire de Kaolack depuis 2017 avec des stratégies innovantes comme le « Ndeye-Dické » qui consiste à la prise en charge des femmes enceintes séropositives et de leur marrainage par les sages-femmes *via* la délégation de tâches.

L’objectif de cette étude est d’évaluer la prise en charge du couple mère-enfant après 5 ans de mise en œuvre de la stratégie Ndeye-Dické.

## Méthode

Il s’agit d’une étude rétrospective descriptive à visée analytique portant sur les femmes enceintes séropositives suivies au district sanitaire de Kaolack du 1^er^ janvier 2017 au 31 décembre 2021.

## Résultats obtenus

Au total, 117 grossesses ont été suivies par une équipe composée de 3 médecins et 30 sages-femmes, ces dernières constituant les marraines ou « Ndeye-Dické ». L’effectif est allé crescendo de 2018 à 2021. L’âge moyen des femmes était de 30 ans. Chez 83 femmes soit 71 %, le diagnostic du VIH a été posé lors de la grossesse. Le profil 1 était majoritaire avec 90,5 %. Pour 89 % des femmes les 4 consultations prénatales ont été respectées. Cependant, 11 % d’entre elles ont raté au moins une consultation malgré les relances par les « Ndeye-Dické ». Seules 24 % ont bénéficié d’une mesure de la charge virale pendant la grossesse et 89 % d’entre elles avaient une charge virale supprimée. L’issue de la grossesse était favorable chez 113 femmes soit 96,5 %, à noter qu’il y a eu 3 avortements, 1 mort-né et 1 décès néonatal. La prophylaxie aux ARV a été effective chez 89,2 % des nouveau-nés. Au total, 99 soit 88 % des 112 nouveau-nés éligibles ont fait le test PCR à 6 semaines, 78 ont reçu leurs résultats. L’ensemble des résultats reçus de test PCR et les sérologies à 14 mois étaient négatifs. Concernant le suivi des femmes après accouchement, 6 ont été perdues de vue dont 4 après la sérologie de leur enfant. Une patiente perdue de vue au 1^er^ trimestre de la grossesse a été retrouvée 16 mois après l’accouchement et la sérologie de son enfant était positive. Grâce aux « Ndeye-Dické », 75 % des perdues de vue ont été retrouvées et réintroduites dans le programme de suivi.

## Conclusion

La stratégie Ndeye-Dické a contribué activement à l’amélioration des indicateurs de la prise en charge du couple mère-enfant dans le cadre de l’élimination de la transmission du VIH dans le district sanitaire de Kaolack.

# Research article: 236 - La législation protèget-elle les femmes usagères de drogues ? Analyse de parcours biographiques au Sénégal

**Published:** 2024-02-23

**Authors:** Rose André FAYE, Alice DESCLAUX, Groupe d’étude CODISOCS

**Affiliations:** 1 Centre régional de recherche et de formation à la prise en charge clinique de Fann (CRCF), Dakar, Sénégal; 2 TransVIHMI, Institut de recherche pour le développement (IRD)

## Contexte

En 2011, Les femmes usagères de drogues injectables (UD) identifiées par l’enquête UDSEN [Usagers de Drogues au Sénégal, ANRS 12243] étaient moins nombreuses que les hommes (69/507) et plus touchées par le VIH. En 2021, elles sont toujours minoritaires parmi les patients suivis au Centre de prise en charge intégrée des addictions de Dakar (CEPIAD), premier centre de soins pour les UD de l’Afrique de l’Ouest offrant la méthadone. Depuis 2016, le projet CODISEN/CODISOCS auquel notre thèse est associée étudie les dynamiques sociales autour de l’usage de drogues au Sénégal. Cette présentation vise à décrire les effets de la criminalisation des drogues sur les trajectoires des soins et de consommation des femmes UD au Sénégal.

## Méthode

Les données sont issues d’une thèse en anthropologie sur les trajectoires des femmes UD. Une enquête qualitative a été réalisée entre 2017 et 2021. Des récits de vies avec des passages répétés ont été collectés auprès de 34 femmes UD. Les données ont été retranscrites, anonymisées et analysées sur le logiciel Dedoose.

## Résultats

Les récits de vie témoignent que les femmes UD au cours de leur trajectoire font généralement face à des sanctions cumulées pour l’usage de drogues et pour le travail sexuel. La possession de drogues est généralement punie par l’emprisonnement ou la détention provisoire. Certaines femmes ont été poursuivies pour délit de possession de drogues ou pour état d’ivresse. Les femmes concernées ont été incarcérées avec des peines n’excédant pas 3 mois pour possession de drogues. La plupart des femmes ont re-consommé des drogues à leur sortie de prison. Parfois, elles sont aussi poursuivies pour défaut de carnet sanitaire avec des peines pouvant aller jusqu’à 1 mois. Au Sénégal, la législation en matière de drogues est figée depuis 2016. La stratégie nationale de lutte contre la drogue insère une modification du Code des drogues qui prévoit un cadre juridique pour la réduction des risques. Cependant, aucune réforme législative n’a été entreprise. Même si l’article 120 du Code des drogues au Sénégal parle du traitement comme complément ou remplacement de la peine (injonction thérapeutique), cette disposition est rarement utilisée. Aucune des femmes que nous avons interrogées n’a bénéficié d’une injonction thérapeutique au cours de sa trajectoire. Les femmes UD qui ont fait un séjour en prison ont rapporté les conditions difficiles de détention pour les femmes (violence, promiscuité, manque d’hygiène…). La prise en charge de l’addiction n’étant pas développée dans les maisons d’arrêt au Sénégal, la gestion du manque est problématique. Elles ont également rapporté des situations où elles ont été stigmatisées et mises à l’écart par leurs camarades de chambre. L’incarcération n’aide pas systématiquement les UD quant à la sortie de l’addiction, elle cause une rupture des soins et installe davantage les femmes dans la trajectoire de consommation.

## Conclusion

Les récits de vie des femmes montrent que le système judiciaire ne protège pas assez les femmes usagères de drogues alors qu’elles font face à de multiples sanctions du fait de l’usage de drogues et du travail sexuel. Les politiques pénales devraient prendre en compte ces multiples discriminations par rapport au phénomène de l’addiction et revoir les législations afin que les femmes ne souffrent plus indûment de l’accumulation des sanctions et des formes de stigmatisation. Il est nécessaire de continuer à sensibiliser sur la stigmatisation, de rendre disponible la prise en charge des addictions dans les prisons et d’accélérer l’adoption du décret relative à la réduction des risques.

# Research article: 235 - La vulnérabilité des femmes consommatrices de drogues injectables (CDI) en temps de Covid-19 : spécifique à l’épidémie ou structurelle ?

**Published:** 2024-02-23

**Authors:** Rose André FAYE, Alice DESCLAUX, Groupe d’étude CODISOCS

**Affiliations:** 1 Centre régional de recherche et de formation à la prise en charge clinique de Fann (CRCF), Dakar, Sénégal; 2 TransVIHMI, Institut de recherche pour le développement (IRD)

## Contexte

Créé en 2014, le Centre de prise en charge intégrée des addictions de Dakar (CEPIAD) est le premier centre de soins moderne incluant la méthadone et un programme d’échange de seringues en Afrique de l’Ouest francophone. Les femmes prises en charge y sont moins nombreuses que les hommes (environ 1/10) et sont plus touchées par la précarité. Des données empiriques ayant montré que l’épidémie de Covid-19 a renforcé la fragilité des femmes CDI, nous avons voulu comprendre quel type de vulnérabilité était exacerbé.

## Méthode

Ce travail est issu d’une thèse sur les trajectoires des femmes CDI, associé à un projet de recherche (CODISOCS). Seize entretiens individuels ont été réalisés auprès de femmes CDI entre avril et juin 2020. Les données recueillies ont fait l’objet d’une analyse thématique.

## Résultats

La survenue de l’épidémie de Covid-19 a créé un fardeau supplémentaire en fragilisant davantage les femmes CDI déjà affectées par l’usage de drogues, les pathologies infectieuses, les inégalités de genre, la précarité, la stigmatisation au Sénégal. Les mesures édictées ont mis à l’arrêt ou limité les activités économiques et personnelles de la plupart des femmes CDI. Les femmes CDI travailleuses du sexe (TS) professionnelles ont eu des difficultés pour avoir des clients du fait des mesures de distanciation sociale et du couvre-feu, mais aussi par peur d’être contaminées par le virus SARS-CoV-2.

Du côté des soins, les femmes se perçoivent comme particulièrement vulnérables à cause de leur état de santé déjà fragilisé par l’usage de drogues et des pathologies dont elles souffrent (VIH, hépatites, diabète, cancer). Lemport à domicile de la méthadone, déjà initié avant l’épidémie de Covid durant le week-end et les jours fériés a été étendu à une semaine en raison de l’épidémie. Certaines femmes en ont profité pour diminuer les doses délivrées ou pour expérimenter l’arrêt du traitement de substitution. Pour certaines, le stress lié à la situation épidémique et à ses conséquences sur leurs activités les a conduites à consommer à nouveau des drogues.

Face à la précarité financière en temps de crise, quelques femmes ont utilisé dans un premier temps leurs économies. D’autres ont demandé de l’aide à des proches. Ces demandes les ont exposées à des propos stigmatisants par rapport à leur statut de CDI. Des distributions de vivres ont été organisées par les acteurs, mais elles ont été jugées insuffisantes.

## Conclusion

La situation de la Covid-19 a renforcé les vulnérabilités multiples auxquelles font face les femmes en tant que femmes, CDI, TS, PvVIH. Même si les femmes n’ont pas été très touchées par la transmission du virus, sur le plan social, la Covid-19 les a rendues plus précaires. Cette vulnérabilité économique cause une certaine fragilité sanitaire pour les femmes qui ont continué le travail de prostitution en temps de Covid. Elle entraîne également une augmentation de la vulnérabilité morale. Pour réduire les inégalités, il faut initier des interventions plus ciblées sur la vulnérabilité économique et développer des stratégies d’autonomisation, de communication et d’éducation sanitaire, visant à minimiser l’impact de la Covid-19.

# Research article: 261 - Atteindre 95-95-95 en recourant à l’analyse et au reporting mensuel des indicateurs du programme VIH par les districts sanitaires du Sénégal : l’approche district

**Published:** 2024-02-23

**Authors:** Cheikh Bamba Dieye GUEYE, Abdoulaye SAGNA, Cheikh Tidiane NDOUR, Safiatou THIAM

**Affiliations:** 1 Secrétariat exécutif du Conseil national de lutte contre le sida, Sénégal; 2 Division de lutte contre le sida et les IST (DLSI), Ministère de la santé, Sénégal

## Contexte

Au Sénégal, l’épidémie du VIH est de type concentré avec une prévalence de 0,5 %. Cette prévalence basse cache cependant des disparités géographiques et populationnelles. Avec la nouvelle approche basée sur la localisation et les populations, l’épidémie de sida consiste en la somme de plusieurs épidémies locales interconnectées, au sein desquelles les populations clés et certaines régions sont les plus touchées. En effet, les régions Sud et Sud-Est présentent des prévalences variant de 0,9 à 1,5 %. Ces régions sont frontalières avec des pays où l’épidémie du VIH est de type généralisé avec des prévalences de 5 %. Les populations clés témoignent de prévalences allant de 5,2 % à 27,6 %. En 2021, le programme VIH a mis en place l’approche district qui permet le reporting mensuel et l’analyse des données des districts pour suivre les progrès vers 3x95 au niveau des districts dans tout le pays.

## Description

Cette approche basée sur les populations prioritaires et leurs localisations (districts) vise à prioriser les districts à travers des scores basés sur 7 domaines et 11 critères de performance à partir des indicateurs suivis par le programme VIH. Elle identifie les districts à problème et les gaps à combler pour améliorer leur performance. Avant la mise en œuvre de l’approche district, l’atteinte des 3x95 au niveau des districts posait problème du fait de mauvaises complétude et promptitude des données du programme VIH qui sont rapportées. Les cibles nationales et de districts issues des estimations Spectrum n’étaient jamais atteintes car les prestataires ne maîtrisaient pas les méthodes de calcul et d’analyse de leur cascade – en plus du manque de coordination et d’instance régulière de calcul et d’analyse des données. Pour accompagner cette stratégie, un processus de reporting mensuel et d’analyse des cascades a été mis en place.

## Leçons apprises

Cette approche a permis de calculer et d’analyser la cascade des districts avec un niveau de désagrégation par âge et sexe. Les données sont collectées par le district dans un outil qui génère automatiquement les cascades. Puis les cascades du district sont analysées pour identifier les gaps et élaborer un plan d’amélioration à mettre en œuvre. À la fin de l’année 2021, les PvVIH sont estimées dans le pays à 40 277 en 2021. Parmi elles, 37 406 connaissent leur statut sérologique soit 93 %, 31 637 sont sous ARV (85 %) et 27 757 ont atteint une suppression virale (88 %). Sur les 79 districts que compte le pays, plus de la moitié ont atteint le premier 95. Les deux tiers ont atteint le second et le troisième 95.

## Conclusion & prochaines étapes

L’approche district avec l’analyse ciblée des cascades a pu fixer des objectifs clairs et réalisables et permis à de nombreux districts d’améliorer leur 3x95 en 2021. Elle a permis un suivi régulier des objectifs du programme et l’atteinte des objectifs 3x95. Cette stratégie a beaucoup amélioré la qualité des données produites par les districts.

# Research article: 202 - Les organisations communautaires, des acteurs à fort potentiel de contribution à l’atteinte des objectifs 95-95-95 parmi les populations clés et prioritaires : experience réussie de leur implication dans la mise en œuvre du projet EpiC piloté par FHI 360 au Sénégal

**Published:** 2024-02-23

**Authors:** Ousmane DIENG, Isseu Fall GUEYE, Bachirou Maniang MBODJI, Jules Bashi BAGENDABANGA, Saliou Mbacke GUEYE, Angèle DIOP, Mouhamadou Moustapha DIOUM, Ibrahima BÂ, Malick DIOUF

**Affiliations:** 1 EpiC USAID FHI 360, Sénégal; 2 RNP+ (Réseau national des associations de PvVIH), Sénégal; 3 Santé service développement (SSD); 4 Association And Soppeku; 5 Association Bokk Yakaar; 6 Association AIDES

## Description du problème

Pour surmonter les obstacles persistants à la réalisation des objectifs 95-95-95 parmi les populations clés (PC) et prioritaires (PP) au Sénégal que sont les professionnels du sexe (PS), les hommes ayant des rapports sexuels avec des hommes (HSH) et les personnes vivant avec le VIH (PvVIH), le projet EpiC (Meeting Targets and Maintaining Epidemic Control), financé par l’USAID dans le cadre du plan d’urgence du Président américain pour la lutte contre le sida (PEPFAR), a misé sur l’enrôlement de quatorze organisations locales représentées par deux grands réseaux d’organisations identitaires : le Réseau national des populations clés (RENAPOC) et le Réseau national des associations de PvVIH (RNP+).

Cette communication a pour objectif de partager les résultats ainsi que la démarche méthodologique adoptée par le projet EpiC dans le cadre de la mise à contribution de ces organisations à l’effort global de réalisation des objectifs 95-95-95 chez les PC et PP au Sénégal.

## Activités réalisées et résultats obtenus

La démarche méthodologique suivante a été adoptée : (i) identification et sélection d’organisations et de réseaux d’association identitaires à forte capacité de mobilisation et signature d’accords de subvention; (ii) analyse et renforcement des capacités institutionnelles et organisationnelles; (iii) mise en réseaux et renforcement des capacités techniques des acteurs; (iv) recrutement de personnels techniques pour la coordination des interventions et remise d’un paquet d’activités de prévention et de soutien communautaire. D’octobre 2021 au 31 août 2022, les résultats suivants ont été obtenus dans les régions de Ziguinchor, Kolda, Sédhiou, Kaolack, Thiès, Saint-Louis et Dakar :

82 pairs éducateurs dont 53 HSH et 29 PS, et 44 pairs navigateurs dont 3 HSH, 3 PS et 20 PvVIH ont été recrutés et formés sur la mise en œuvre des différentes approches communautaires du projet;

7 agents mapping ont été recrutés, et 650 points chauds ont été identifiés à travers un exercice de cartographie programmatique dont 535 actifs, tous ayant été géolocalisés et répertoriés dans une base en ligne en vue d’un accès facile pour la cible et pour le personnel du programme;

offre du paquet de prévention à 5726 PC, soit 3341 HSH et 2385 PS, et à 6458 PP dont 57,7 % de femmes; réalisation du test de dépistage pour 5415 PP et 3748 PC avec respectivement 23,9 % et 7,15 % de cas positifs dont 71 % de HSH et 29 % de PS;

mise sous traitement antirétroviral chez les PC de plus de 95 % des cas positifs dont 95 % ont été accompagnés jusqu’à la suppression virale;

mise en place d’une base en ligne centralisant toutes les données des interventions communautaires dans la plateforme VIH Tracker du DHIS2.

## Leçons apprises et étapes futures

À la lumière des résultats enregistrés en 11 mois d’intervention, il convient de noter que les organisations identitaires seraient des acteurs incontournables pour la réalisation des objectifs 95-95-95 parmi les populations clés et prioritaires au Sénégal.

# Research article: J215 - Prévention et prise en charge des populations clés à travers les technologies de l’information et de la communication : expérience réussie de l’offre de service VIH en ligne dans le cadre de la mise en œuvre du projet EpiC piloté par FHI 360 au Sénégal

**Published:** 2024-02-23

**Authors:** Isseu Fall GUEYE, Bachirou Maniang MBODJI, El Hadji Malick DIOUF, Diadji DIOUF, Jules Bashi BAGENDABANGA, Ousmane DIENG

**Affiliations:** 1 EpiC-USAID/FHI 360, Sénégal; 2 Réseau national des populations clés (RENAPOC); 3 Association AIDES

## Description du problème

Dans le cadre de sa contribution aux efforts d’atteinte des cibles et de maintien du contrôle de l’épidémie VIH parmi les populations clés (PC) au Sénégal, le projet EpiC (Meeting Targets and Maintaining Epidemic Control), piloté par FHI 360, a développé depuis janvier 2021 une stratégie innovante de sensibilisation en ligne dénommée « Going Online ».

Cette stratégie utilise les médias sociaux et d’autres outils de messagerie pour atteindre les personnes qui peuvent avoir besoin d’informations précises et sans jugement sur la santé sexuelle et le VIH. De plus, elle associe le déploiement d’une plateforme Lerlouwer à travers ORA « Online Reservation and Case Management App » pour la réservation des services VIH et le suivi des clients séropositifs en lien avec les structures dédiées à la prise en charge.

Cette initiative est soutenue par un réseau d’acteurs communautaires dirigeant les agents de prévention par Internet (API) et les pairs éducateurs (PE) pour l’information et le marketing, l’identification, le référencement de leurs pairs cachés et à risque vers les structures de prise en charge du VIH.

Cette communication vient présenter la démarche méthodologique ainsi que les résultats clés obtenus de mars à juin 2022.

## Activités réalisées et résultats obtenus

Les étapes suivantes ont été suivies dans la mise en œuvre de cette approche : (i) le recrutement et le renforcement de capacité des agents de sensibilisation en ligne, (ii) la cartographie virtuelle des points chauds et l’utilisation des plateformes de communications numériques et des médias sociaux afin d’identifier et de mettre en lien les PC les plus exposées avec les services de santé, (iii) la configuration et l’adaptation de la plateforme de réservation électronique, de gestion et de suivi des cas (Lerlouwer) avec le paramétrage des 25 sites d’intervention du projet EpiC. De février 2022 au 30 juin 2022, les résultats suivants ont été enregistrés :

1701 patients ont été touchés dont 1542 PC identifiées et sensibilisées;

1950 vus ont été notifiés durant cette période avec les différentes plateformes et réseaux fréquentés par les API;

34 messages et posts effectués auprès des groupes et pages sur les plateformes WhatsApp, Grindr, Instagram et Facebook;

250 patients ont été référés avec 204 testés dont 120 par les PE d’AIDES et 84 par les pairs de RENAPOC.

32 PC confirmées positives et mis sous ARV dans les sites de DLSI, Pikine, Mbao, Mbour et Saint-Louis avec une contribution de 15 cas positifs qui ont été référés par les pairs d’AIDES;

8 populations clés HSH ont bénéficié de la prise en charge des IST/VIH;

124 PC dépistées négatives ont bénéficié du screening de la PrEP et 19 ont été enrôlées.

## Leçons apprises et étapes futures

Going Online est une stratégie innovante qui répond aux défis actuels de la mobilité des populations clés et de la continuité des services VIH dans le contexte de la pandémie de Covid-19.

# Research article: 250 - La cartographie programmatique et l’estimation de la taille des populations clés dans les 9 nouveaux sites PEPFAR : l’expérience et le leadership du Réseau national des associations de PvVIH (RNP+) du Sénégal

**Published:** 2024-02-23

**Authors:** Saliou Mbacke GUEYE, Bachirou MBODJ, Soukeye NDIAYE

**Affiliations:** 1 Réseau national des associations de PvVIH (RNP+), Sénégal; 2 EpiC USAID/FHI 360, Sénégal

## Objectif

Dans le cadre de la mise en œuvre du projet EpiC Sénégal piloté par FHI 360, le Réseau national des associations de personnes vivant avec le VIH (RNP+) a réalisé la cartographie et l’estimation de la taille des populations dans les 9 nouveaux sites dont le but est d’améliorer l’efficacité et l’efficience des interventions en faveur des populations clés pour une couverture de haut niveau dans les sites d’intervention du PEPFAR (plan d’urgence du Président américain pour la lutte contre le sida) au Sénégal.

## Méthodes

Phase préparatoire : réunions de coordination entre les différentes parties prenantes, recrutement des enquêteurs et superviseurs, visites de prospection dans les 9 sites auprès des autorités administratives et médicales, organisation de 9 focus groups avec les populations clés pour l’établissement de points chauds et la collecte des listes préexistantes de points chauds auprès des parties prenantes.

Formation des enquêteurs/superviseurs : 27 enquêteurs (3 par site) et 9 superviseurs ont été formés.

Phase de collecte de données : 10 jours ont été consacrés à la collecte des données avec l’administration de 563 questionnaires.

Phase de gestion et d’analyse de données : saisie des données dans une base, gestion des valeurs manquantes et estimation de la taille brute et ajustée.

## Résultats obtenus

Sur les 563 questionnaires administrés, 58 sont du site de Dakar Nord, 68 de Mbao, 69 de Keur Massar, 65 de Thiès, 61 de Kaolack, 67 de Saint-Louis, 79 de Bignona, 38 de Sédhiou et 58 de Kolda. L’analyse révèle que 117 (33 %) des sites, ont été identifiés comme sites fréquentés uniquement par les hommes ayant des relations sexuelles avec d’autres hommes (HSH) contre 114 (32 %) où se rencontrent les professionnels du sexe (PS) et 125 (35 %) comme des sites mixtes pour un total de 365 sites actifs. La répartition des types de points chauds démontre que les bars sans hébergement, les places publiques et les auberges/hôtels et chambres de passe sont les plus fréquentés avec respectivement 18 %, 15 % et 14 %. Pour les types d’activités pratiquées dans les points chauds, les sollicitations prennent le plus grand pourcentage avec 51 %, suivies des activités sexuelles avec 26 % et enfin les activités sexuelles et sollicitations avec 23 %. Comme jour de forte affluence, le samedi est confirmé par 99 % des répondants. Les estimations ajustées ont donné une population composée de 2727 HSH et 2796 PS dans les 9 nouveaux sites PEPFAR. 100 % des HSH et PS affirment utiliser l’application WhatsApp pour solliciter des rencontres. Pour les cas de violences basées sur le genre, on note des inquiétudes dans le site de Kaolack et Mbao avec respectivement 69 % et 63 % des répondants concernés.

## Conclusion

Globalement, l’étude a permis de cartographier tous les points chauds des 9 nouveaux sites PEPFAR et d’estimer le nombre de populations clés en responsabilisant les organisations communautaires. Elle a également permis de formuler des recommandations en matière de prévention et sensibilisation, pour un environnement favorable, aux partenaires techniques et financiers et à FHI 360.

# Research article: 36 - Devoir d’informer et injonction au silence : le dilemme des soignants face aux adolescents nés avec le VIH au Sénégal en 2021

**Published:** 2024-02-23

**Authors:** Gabrièle LABORDE-BALEN, Bernard TAVERNE, Khoudia SOW, et le groupe ETEA-VIH

**Affiliations:** 1 TransVIHMI, Université de Montpellier, INSERM, IRD, Montpellier, France; 2 Centre régional de recherche et de formation à la prise en charge clinique de Fann (CRCF), Dakar, Sénégal

## Objectifs

L’annonce de la maladie aux enfants et adolescents vivant avec le VIH (EAv-VIH) est une étape cardinale. Des études ont montré que l’annonce contribue à faire de l’enfant un acteur de ses soins, se traduit le plus souvent par une meilleure observance aux ARV et favorise le succès thérapeutique. L’OMS recommande depuis 2011 que le processus débute vers l’âge de 8 ans, avec une annonce complète à 12 ans.

Au Sénégal, des formations sont réalisées depuis plusieurs années, à l’aide de manuels de bonnes pratiques d’annonce, auxquels des recherches menées à Dakar ont contribué. Les recommandations sont de mieux en mieux appliquées dans la capitale, mais la situation reste inégale hors de Dakar. Une étude a exploré les pratiques et difficultés d’annonce de la maladie aux AEvVIH dans les structures de santé décentralisées.

## Méthode

La recherche anthropologique « Échec thérapeutique chez les enfants et adolescents vivant avec le VIH en contexte décentralisé [ETEA-VIH, ANRS 12421] » a été menée en 2020 et 2021 dans 15 hôpitaux régionaux (CHR) et centres de santé (CS) de 11 régions du Sénégal. Des entretiens semidirectifs ont concerné 87 AEvVIH et/ou leurs parents/tuteurs et les acteurs de santé. Une « histoire de vie » de chaque enfant a été reconstituée. Des analyses thématiques ont été réalisées à partir de thèmes pré-identifiés ou qui ont émergé au cours de l’étude. Les pratiques et difficultés liées à l’annonce de la maladie, chez les 39 adolescents âgés de plus de 12 ans, ont fait l’objet d’une analyse spécifique.

## Résultat

La mise en œuvre systématique de l’annonce par les soignants est plus fréquente dans les CHR que dans les CS. Dans certains CS excentrés, des adolescents âgés de plus de 16 ans ne sont toujours pas informés de leur maladie. Les raisons sont multiples : la difficulté des acteurs sociaux, pourtant formés à l’annonce, à appliquer les enseignements reçus; l’espacement des visites des adolescents – certains parents venant seuls récupérer les ARV; et principalement les réticences des parents. Les raisons évoquées sont la peur que l’enfant ne divulgue son statut – et indirectement celui de sa mère ou de ses parents – dans l’entourage et la volonté de le protéger contre un choc psychologique et un risque de stigmatisation. L’organisation de « groupes de parents » pour échanger et vaincre les réticences se heurte, dans certains villages, à la volonté de garder le secret absolu. Les soignants se trouvent face à un dilemme moral : doivent-ils respecter l’injonction parentale ou considérer qu’il est de leur devoir d’informer le jeune majeur ?

## Conclusion

L’information de l’adolescent doit être privilégiée pour lui permettre de gérer sa sexualité, son traitement, prendre soin de sa santé et de son avenir. Les soignants sont cependant soucieux de ménager les parents dans les localités où les PvVIH sont stigmatisés. Entre le devoir d’informer et la volonté de prévenir les conflits, la médiation reste la voie à privilégier pour surmonter ce dilemme.

# Research article: 61 - Évaluation du taux d’échec thérapeutique chez les patients sous première et deuxième ligne de traitement antirétroviral suivis au Service des maladies infectieuses et tropicales du Centre régional de recherche et de formation à la prise en charge clinique du Centre hospitalier universitaire de Fann

**Published:** 2024-02-23

**Authors:** Krist Daryl MAKOSSO, Osseynou NDIAYE, Malick DIOUARA, Melissa Sandrine PANDI, Hubert AKOTIA, Thierno Madio DIALLO, Karim DIOP

**Affiliations:** 1 Centre régional de recherche et de formation à la prise en charge clinique de Fann (CRCF), Dakar, Sénégal; 2 École supérieure polytechnique (ESP), Université Cheikh Anta Diop, Dakar

## Contexte

Selon l’Organisation mondiale de la santé, un patient est en échec thérapeutique lorsque nous observons une charge virale supérieure à 1000 copies/ml basée sur deux mesures consécutives de la charge virale à 3 mois d’intervalle, avec un soutien à l’observance après la première mesure de charge virale.

## Objectifs

Cette étude avait pour objectif principale d’évaluer le taux d’échec thérapeutique des patients suivis au Service des maladies infectieuses et tropicales (SMIT) du CRCF en première et en deuxième ligne de traitement antirétroviral. De façon spécifique, il s’agissait d’évaluer le taux d’échec virologique par la réalisation de tests de charge virale VIH-1 chez les patients sous traitement antirétroviral au SMIT/CRCF, d’identifier et de confirmer les échecs virologiques.

## Méthodologie

Il s’agit d’une étude transversale qui s’est déroulée sur une période de 12 mois allant de janvier à décembre 2021 chez les personnes vivant avec le VIH suivies au SMIT/CRCF du CHNU de Fann ayant effectué leurs mesures de charges virales VIH-1. Ces dernières ont été réalisées avec le Cobas Taqman 48/Cobas Ampliprep pour un seuil de détection de 20 copies/ml. L’échec virologique a été identifié sur les patients ayant des mesures de charge virale supérieures ou égale à 1000 copies/ml. Les tests statistiques ont été réalisés avec le logiciel IBM SPSS avec un indice de confiance de 95 %.

## Résultats

Notre étude portait sur 669 PvVIH sous traitement ARV et suivies au SMIT/CRCF. Les femmes étaient plus représentées avec une proportion de 65,30 % soit un sexratio Femmes/Hommes de 1,88 et la médiane d’âge était de 48 ans avec des extrêmes allant de 17 à 77 ans. La médiane de suivi était de 110 mois et le traitement de première intention était prédominant (85,50 %). Dans notre étude, 59 patients avaient des mesures de charge virale supérieures à 1000 copies/ml et donc en suspicion d’échec virologique soit un taux de 8,80 %. Parmi ces derniers, 58 patients ont eu une deuxième mesure de charge virale dont 27 sont en échec (soit un taux de 4,03 %). Parmi ces patients en échec, 21 étaient en première ligne de traitement (soit un taux de 77,77 %) contre 6 en deuxième ligne (soit un taux de 22,22 %). L’échec virologique était quasiment semblable autant chez les hommes (14) que chez les femmes (13) avec des taux respectifs de 51,85 % et 48,14 %. Par ailleurs, cette étude a montré une influence du temps de suivi sur l’échec virologique avec une p value = 0,273.

## Conclusion

L’Organisation mondiale de la santé recommande de maintenir des taux d’échecs à moins de 10 %. Il est important de rester en dessous de ce taux afin de réduire les risques accrus de transmission des formes résistantes du virus et une progression du VIH. Pour la dispensation au CRCF, plusieurs molécules antirétrovirales sont utilisées en première et en deuxième ligne; il serait intéressant d’observer les molécules sur lesquelles on a des échecs et suivre ces souches de VIH.

# Research article: 233 La stratégie « Ndeye-Dické » ou marrainage des femmes enceintes séropositives par les sages-femmes pour améliorer la rétention du couple mère-enfant pour l’élimination de la transmission mère-enfant au Sénégal

**Published:** 2024-02-23

**Authors:** Khadidia Fall TRAORÉ, Cheikh Tidiane NDOUR, Ndeye Marame GUEYE, Fatou Nar MBAYE

**Affiliations:** 1 Division de lutte contre le sida et les IST (DLSI), Ministère de la santé, Sénégal; 2 Conseil national de lutte contre le sida, Sénégal

## Contexte

L’analyse situationnelle de 2018 a mis en évidence des insuffisances dans l’offre de services dans le domaine de la prévention de la transmission de la mère à l’enfant du VIH (PTME) qui reste centralisée auprès du médecin de prise en charge ARV. La femme séropositive doit parcourir de longues distances, parfois venant de zones enclavées pour se rendre au centre de santé ou à l’hôpital pour avoir le test de confirmation du test VIH, les ARV et faire le suivi de son enfant, ce qui entraîne des « perdus de vue » du fait de l’inaccessibilité des soins PTME. L’amélioration de l’offre de services aux femmes enceintes séropositives et à leurs familles est une priorité pour l’atteinte des objectifs de l’élimination de la transmission mère-enfant (eTME) d’ici fin 2025, d’où l’adoption de la stratégie « Ndeye-Dické ». Cette stratégie consiste en une délégation de tâches aux paramédicaux de la prise en charge de la mère séropositive et de son enfant intégrée à l’offre de services de Santé reproductive (SR) : la sage-femme s’engage à faire un accompagnement psycho-social du couple mère-enfant jusqu’à la sérologie définitive de l’enfant, en étant la « marraine » du couple. L’objectif de cette présente étude est d’évaluer cette stratégie innovante après 3 ans de mise en œuvre et de dégager des perspectives.

## Méthodologie

Étude descriptive à partir des rapports de taux de survie, des rapports de supervision des sites, et des rapports de routine et des revues eTME régionales de 2021.

## Résultats

Il apparaît que 90 % des femmes enceintes ont utilisé les services de Consultations prénatales (567 356/628 363), 85 % (532 544) ont fait le dépistage VIH, 72 % ont bénéficié d’ARV par rapport aux femmes séropositives attendues et 61 % des enfants nés vivants ont bénéficié de PCR dans les délais en 2021. 45 % des sages-femmes formées prennent en charge les femmes enceintes séropositives et leurs enfants en prescrivant les ARV, et assurent le suivi du couple mère-enfant dans le cadre de la PTME. La prise en charge du couple mère-enfant est décentralisée dans 35 % des Postes de santé. Cependant, la décentralisation se limite à la dispensation ARV pour les femmes enceintes et allaitantes séropositives et à la prophylaxie à la naissance des enfants exposés. L’enfant est référé au Centre de santé pour la PCR et la sérologie définitive.

## Conclusion

La mise à l’échelle de la délégation de tâches et la décentralisation au niveau des postes de santé de l’offre de services PTME associées à une intégration des services SR et au marrainage pourront contribuer de manière significative à l’atteinte des objectifs de l’eTME d’ici fin 2025.

# Research article: 42 - Étude rétrospective sur la tuberculose extra-pulmonaire au Centre hospitalier Abass Ndao (2009-2021)

**Published:** 2024-02-23

**Authors:** Abdoul Aziz NDIAYE, Djiby SOW, Eunice Gloire YEDI M’FOUMBOULOU

**Affiliations:** 1 Université Alioune Diop de Bambey, Sénégal; 2 Université Cheikh Anta Diop, Dakar, Sénégal; 3 Université du Sine Saloum El Hâdj Ibrahima Niass (USSEIN), Sénégal

## Introduction

La tuberculose est une infection bactérienne due au *Mycobacterium tuberculosis.* Elle constitue un problème majeur de santé. L’atteinte extra-pulmonaire reste sous-estimée car le diagnostic est souvent basé sur des arguments présomptifs. L’objectif est d’étudier les aspects épidémiologiques et évolutifs de la tuberculose extrapulmonaire au service de médecine interne du Centre hospitalier Abass Ndao de Dakar.

## Méthodologie

Il s’agissait d’une étude rétrospective, descriptive et analytique sur une période de 12 ans à partir des dossiers de patients hospitalisés pour tuberculose extra-pulmonaire du 1^er^ janvier 2009 au 31 décembre 2021.

## Résultats

Il a été répertorié 52 cas de tuberculose extra-pulmonaire avec une prédominance féminine (61,4 %). L’âge moyen était de 40,56 ± 18,24 ans et la tranche d’âge de 20 à 34 ans était la plus touchée (42,31 %). Les femmes au foyer constituaient la majorité (40,38 %). La notion de contage était retrouvée chez 20 patients (38,46 %). Le délai de consultation était relativement long. Cependant le délai de prise en charge était essentiellement de moins d’un mois. La symptomatologie était polymorphe, dominée par la fièvre et l’altération de l’état général. L’atteinte péritonéale était dominante (54,9 %). La localisation bifocale plèvre/péritoine était fréquente. L’infection concomitante des poumons était notée chez la moitié des patients (51,92 %). La sérologie au VIH était positive chez 5,9 % des patients. La bacilloscopie a été souvent négative et le GeneXpert n’était réalisé que chez 13,7 %. La cytoponction était effectuée dans 33 % des cas et montrait un caséum à l’histologie (90,3 %). La radio du thorax et l’échographie abdominale étaient réalisées respectivement dans 51,92 % et 48,08 % des cas. L’issue thérapeutique était favorable dans 78,57 % des cas, 11,90 % transférés et 4 décès en cours d’hospitalisation.

## Conclusion

La tuberculose extra-pulmonaire pose un problème diagnostique. Le recours aux soins était souvent tardif. L’issue thérapeutique était plutôt favorable.

# Research article: 170 - Prise en charge à domicile de la Covid-19 : exemple du district sanitaire de Mbao du 19 mars 2020 au 19 août 2022

**Published:** 2024-02-23

**Authors:** Rahmatoulahi NDIAYE, Coumba Ndoffene DIOUF, Alseyni DIALLO

**Affiliations:** District sanitaire de Mbao, Sénégal

## Contexte

Face à la situation d’urgence de santé publique de l’épidémie à Coronavirus, le Sénégal a mis en place un dispositif pour une détection précoce et la prise en charge des patients. Avec l’augmentation du nombre de cas et la saturation des structures hospitalières, la stratégie de prise en charge extra-hospitalière a été mise en place. Son but étant de contribuer au désengorgement des structures hospitalières et à la rationalisation de l’utilisation des ressources.

## Méthodologie

Il s’agissait d’une étude transversale et descriptive allant du 19 mars 2020 au 19 août 2022 qui a été réalisée chez les patients atteints de Covid-19 et pris en charge à domicile dans le district sanitaire de Mbao. La décision d’une prise en charge à domicile était adoptée après une évaluation initiale par un médecin en présentiel ou en téléconsultation. L’évaluation comprenait :

l’évaluation clinique afin de classer le patient, consistant à rechercher les signes de gravité, de comorbidité et de fragilité chez les sujets; l’évaluation environnementale afin d’apprécier la possibilité du patient de disposer d’une pièce où il peut être isolé et respecter les gestes barrières.

Le suivi du patient se faisait par téléconsultation et au besoin une équipe médicale mobile se déplaçait à domicile pour une autre évaluation après accord du patient.

## Résultats

Durant la période d’étude, 8913 cas suspects ont été prélevés et 2972 patients sont revenus positifs soit 33,34 % des cas et répartis comme suit : 4 cas importés, 293 cas secondaires, 2675 cas communautaires. Parmi les cas communautaires, 226 cas étaient des patients asymptomatiques qui connaissent leur statut au décours d’un test de Covid-19 fait dans le but d’un voyage. La moyenne d’âge était de 42,28 ± 16,99 ans [1-95], la tranche d’âge la plus touchée était celle comprise entre 25 et 34 ans avec 681 cas. Il y avait plus d’hommes (53,83 %).

La prise en charge à domicile était faite chez 2536 cas, soit 85,32 %, 360 cas soit 12,11 % ont été régulés dans les Centres de traitement épidémiques, et 76 cas soit 2,55 % ont refusé d’être déclarés positifs à la Covid-19 et sont devenus injoignables.

La prise en charge des 5483 cas contact était faite par les assistants sociaux, mais 97 cas contact ont catégoriquement refusé une prise en charge.

2502 cas soit 98,65 % ont été déclarés guéris à domicile 10 jours après leur test positif à la Covid-19.

Huit patients sont décédés à domicile, soit 0,31 %, avec davantage de femmes décédées (75 %), la moyenne d’âge étant de 59,62 ± 15,88 ans [45-90]. Il s’agissait des patients qui avaient refusé les soins et qui présentaient des comorbidités : 5 cas d’hypertendus et diabétiques et 3 diabétiques.

## Conclusion

La pandémie de Covid-19 a révélé les faiblesses de nos systèmes de santé. La prise en charge à domicile a permis de faire face à ces difficultés. La téléconsultation doit être davantage considérée dans nos pays.

# Research article: 46 - L’apport des pairs communautaires dans l’identification et l’accès à l’offre de service VIH aux hommes ayant des rapports sexuels avec d’autres hommes (HSH) dans la banlieue de Dakar à Mbao du 1^er^ octobre 2020 au 31 juillet 2022

**Published:** 2024-02-23

**Authors:** Rahmatoulahi NDIAYE, Coumba Ndoffene DIOUF, Alseyni DIALLO, Aminata THIAM, Koubo BOSSOU, Cheikh Tidiane NDOUR

**Affiliations:** 1 District sanitaire de Mbao, Sénégal; 2 Division de lutte contre le sida et les IST (DLSI), Ministère de la santé, Sénégal

## Contexte

L’épidémie du VIH au Sénégal est de type concentré avec une prévalence faible dans la population générale (0,5 %) et élevée chez les populations clés en général, les hommes ayant des rapports sexuels avec d’autres hommes (HSH) en particulier dépassant les 27 %. Le contexte très particulier marqué par la pénalisation des actes contre nature, la stigmatisation et la discrimination constitue un défi majeur pour l’identification et l’accès à ces groupes à des services adaptés à leurs besoins. Cette situation est ainsi la principale contrainte à l’atteinte des objectifs d’élimination. L’objectif de cette étude était de contribuer à améliorer l’accès aux HSH les plus difficiles à atteindre dans le district sanitaire de Mbao afin de les sensibiliser, leur offrir des services de dépistage différencié et les lier aux soins de prévention ou de traitement.

## Méthodes

Il s’agit d’une étude transversale descriptive allant du 1^er^ octobre 2020 au 31 juillet 2022 qui a été réalisée chez les HSH habitant ou fréquentant les points chauds du district sanitaire de Mbao. Des pairs HSH formés avaient pour mission de rentrer en contact avec le maximum de HSH cachés, de leur fournir un paquet de services de sensibilisation, de dépistage, de prévention et de soins dont le traitement ARV en cas de besoin.

## Résultats

Durant la période d’étude, quatre pairs HSH ont été formés et mobilisés. Au total, ces pairs ont réussi à dépister un total de 513 HSH pour le VIH, dont 119 positifs, soit une séroprévalence de 23,19 %.

Sur les 119 positifs au VIH, 88 ont eu accès au traitement hautement actif soit 73 % des cas. Pour le reste, 20 cas (16 %) ont été transférés dans les autres sites de prise en charge pour un rapprochement de domicile, 7 (5,88 %) connaissaient déjà leur statut et étaient sous traitement lors du dépistage, et 4 patients (3,36 %) ont catégoriquement refusé les soins car ayant « peur de la stigmatisation ».

En outre, les pairs HSH ont effectué 409 visites à domicile durant lesquelles 145 162 préservatifs masculins ont été distribués, 6200 préservatifs féminins et 222 441 lubrifiants.

## Conclusion

L’atteinte de la cible des trois 95 et de l’élimination est fortement tributaire de la qualité de la prise en charge des populations clés. Des stratégies innovantes à base communautaire telles que l’implication des pairs dans les activités de sensibilisation, de dépistage et d’orientation, devraient nous permettre d’améliorer de manière significative les performances de la cascade de soins dans les catégories les plus vulnérables.

# Research article: 138 - Renforcer les acteurs communautaires pour une meilleure prise en charge des populations clés dans le contexte du VIH ? Exemple du projet régional ReCCAP

**Published:** 2024-02-23

**Authors:** Sidy Mokhtar NDIAYE, Nguissali TURPIN, Ibrahima BÂ, Fatou Maria DRAMÉ, Daouda DIOUF, Ngoundji DIENG

**Affiliations:** ENDA Santé

## Contexte

En Afrique de l’Ouest et du Centre, on assiste à la disponibilité progressive de données précisant le poids et la nature de l’épidémie du VIH chez les populations clés, associée à une mise en place de services en leur direction. Cependant, le manque de capacités des acteurs locaux limite les possibilités de mise à l’échelle des interventions à l’endroit des groupes vulnérables. ENDA Santé et ses partenaires ont proposé de mettre en œuvre un projet de renforcement des capacités. Le projet s’adresse aux acteurs communautaires, locaux et nationaux afin que ces derniers puissent s’approprier et participer à la mise à l’échelle d’activités adaptées aux réalités et aux besoins des différentes populations clés.

## Description de l’intervention

L’objectif est de renforcer la localisation et la qualité des interventions afin de contribuer à l’évaluation de l’efficacité des services de la cascade de soins VIH pour les populations clés en Côte d’Ivoire, en Guinée-Bissau, en Guinée et au Sénégal, à travers :

des formations régionales de haut niveau pour certifier des formateurs référents sur les processus de conduite et les outils de localisation (cartographie, estimation de la taille, évaluation des services et des acteurs) et l’offre de services de qualité pour les populations clés;

des renforcements de compétences des acteurs pays en démultipliant ces formations et en les associant à un appui direct à la mise en œuvre de cartographies localisées;

des soutiens techniques et financiers aux acteurs formés pour la réalisation de cartographie et l’estimation de la taille des populations clés au niveau local.

## Résultats

De 2019 à 2021 : Deux ateliers régionaux de formation des formateurs référents de haut niveau ont été organisés : octobre 2019, 22 participants, 7 pays – Sénégal (6), Guinée (4), Guinée-Bissau (4), Côte d’Ivoire (4), Mali (1), Gambie (1), Niger (2); mai 2021, 23 formateurs référents, 4 pays – Sénégal (6), Guinée (6), Guinée-Bissau (5), Côte d’Ivoire (6). Plusieurs sessions de mentorat personnalisé ont été organisées, notamment l’accompagnement et le coaching des formateurs pays pour la démultiplication des connaissances auprès des acteurs de lutte contre le VIH. 5 sessions de formation des acteurs pays ont été organisées avec une participation de 120 personnes dont 45 % issues des groupes de populations clés (HSH, PS et UD). Six projets de cartographie locale ont été réalisés dans les 4 pays par les acteurs communautaires formés et financés par le projet ReCCAP. Un espace d’échange virtuel et d’incubateur a été créé pour faciliter les discussions et les interactions entre acteurs de même pays et de pays différents. 8 sessions de webinaires sur des thématiques liées au VIH et à la Covid-19 ont été organisées.

## Conclusion

Le projet a pu démontrer les capacités des acteurs communautaires locaux, y compris les populations clés, à réaliser par eux-mêmes des activités de recherche (cartographie et estimation de la taille des populations clés) sans avoir recours à des experts nationaux ou internationaux. Il a permis d’avoir désormais des pools d’acteurs spécialistes en cartographie et estimation de la taille.

# Research article: 74 - Faiblesse des interventions d’accompagnement des enfants et adolescents vivant avec le VIH en contexte décentralisé au Sénégal (Bignona)

**Published:** 2024-02-23

**Authors:** Aminata NIANG, Karim DIOP, Khoudia SOW, Gabrièle LABORDE-BALEN, Bernard TAVERNE, Ndeye Fatou NGOM GUEYE, Abdoul Aziz NDIAYE

**Affiliations:** 1 Centre régional de recherche et de formation à la prise en charge clinique de Fann (CRCF), Dakar, Sénégal; 2 Université Alioune Diop de Bambey, Sénégal; 3 Division de lutte contre le sida et les IST (DLSI), Ministère de la santé, Dakar; 4 Ministère de la santé et de l’action sociale (MSAS), Dakar; 5 TransVIHMI, Université de Montpellier, INSERM, IRD, Montpellier, France; 6 Agence nationale de recherche sur le sida et les hépatites virales (ANRS), France

## Objectif

En 2018, l’étude EnPRISE 2, a révélé que 68 % des enfants et adolescents vivant avec le VIH (EAvVIH) suivis dans les régions Sud et Sud-Est du Sénégal étaient en échec thérapeutique. Cette situation a conduit à la mise en place d’un programme de renforcement de la prise en charge (PEC) décentralisée des EAvVIH en 2020 (EnPRISE 3) dans différentes régions dont celle de Ziguinchor. Ce programme comporte un volet recherche opérationnelle sur la PEC psychosociale des EAvVIH. L’objectif est de décrire et d’analyser les interventions d’accompagnement menées auprès des EAvVIH à Bignona.

## Méthodes

L’enquête qualitative a été conduite d’août à septembre 2020 au Centre de santé de Bignona, qui a l’une des plus grandes files actives d’enfants VIH de la région de Ziguinchor. Une revue documentaire a été réalisée, accompagnée d’entretiens semi-directifs et d’observations auprès de 21 acteurs de la prise en charge, de 16 EAv-VIH et/ou de leurs répondants. Les données recueillies ont fait l’objet d’une analyse thématique.

## Résultats

En 2020, le Centre de santé de Bignona suit 64 EAvVIH (0-19 ans). Le dispositif de prise en charge est géré par une équipe médico-sociale polyvalente composée de 11 personnes – 1 médecin, 1 assistant social, 1 technicien laboratoire, 2 infirmières, 1 aide-infirmière, 2 conseillères VIH et 3 acteurs communautaires (médiateurs). Sur le plan virologique, sur les 64 EAvVIH suivis, 46 ont bénéficié d’une mesure de charge virale (CV) en 2019. Parmi eux, 22 ont une CV > 1000 cp/ml.

Les activités de soutien développées pour les EAvVIH étaient assurées par l’équipe médico-sociale. Elles étaient ponctuelles et occasionnelles, et s’effectuaient dans des situations particulières de vulnérabilité. Elles étaient entre autres : le paiement du petitdéjeuner, le remboursement du transport, la dotation de fournitures scolaires, l’organisation d’arbres de Noël… Faute de ressources financières, il était difficile de les réaliser régulièrement.

Les activités des deux acteurs communautaires étaient principalement destinées aux populations clés (HSH et PS). Celles du troisième médiateur PvVIH concernaient uniquement les adultes vivant avec le VIH.

Aucune activité régulière de renforcement de l’observance (groupes de parole, visites à domicile, clubs d’observance, préparation à l’annonce de la maladie, repas communautaires entre adolescents.) n’était organisée pour les enfants présentant des CV > 1000 cp/ml.

## Conclusion

Les résultats de cette recherche montrent un déficit d’activités de soutien régulières destinées aux EAvVIH dans le Centre de santé de Bignona. Cela est dû d’une part au manque de moyens financiers attribués à l’équipe sociale, qui les conduit à se limiter à des activités ponctuelles et d’autre part au fait que les activités des médiateurs associatifs soient focalisées sur les adultes et les populations clés. Pour améliorer la prise en charge pédiatrique, il est nécessaire d’appuyer les équipes pour l’organisation régulière d’activités de soutien aux EAvVIH.

# Research article: 130 - Dyslipidémies et facteurs associés chez les enfants infectés par le VIH sous traitement ARV à Ouagadougou

**Published:** 2024-02-23

**Authors:** Caroline OKENGO YONABA, Aminata OUÉDRAOGO, Fabienne SOUDRE, Angèle KHALMOGHO, Jocelyne KYELEM, Fla KOUETA, Elie KABRE

**Affiliations:** 1 Département de pédiatrie, Centre hospitalier universitaire Yalgado Ouédraogo, Ouagadougou, Burkina Faso; 2 Service de biochimie, Centre hospitalier universitaire pédiatrique Charles de Gaulle, Ouagadougou; 3 Service de biochimie, Centre hospitalier universitaire Yalgado Ouédraogo, Ouagadougou

## Introduction

Les dyslipidémies chez les enfants vivant avec le VIH sont souvent asymptomatiques et sous-diagnostiquées dans notre contexte. Leurs diagnostic et prise en charge précoces permettent de prévenir les complications graves.

## Objectif

Analyser les dyslipidémies chez les enfants sous traitement antirétroviral (TARV).

## Méthodologie

Étude transversale descriptive et analytique à collecte rétrospective du 1^er^ mai 2009 au 31 mai 2019 chez les enfants sous TARV suivis en pédiatrie au CHU Yalgado Ouédraogo. Tous les enfants sous TARV ayant au moins un bilan lipidique ont été inclus.

## Résultats

Ont été inclus 175 enfants. L’âge moyen était de 56,67 mois ± 45,194 [extrêmes 1 et 168 mois; médiane 48 mois]; garçons = 53 %. À l’initiation du TARV : état nutritionnel normal (68 %), stade clinique 3 ou 4 (49,7 %), protocole à base de 2INRT + NVP (74,8 %). Les dyslipidémies ont été notées au moins une fois au cours du suivi chez 70 (40 %) patients : hypoHDLémie 65 (93 %), hyperlipasémie 3 (4 %), et hypertriglycéridémie 2 (3 %). En analyse multivariée, le sexe féminin était significativement associé à l’hypoHDLémie; OR = 2,2 (p = 0,01).

## Conclusion

Les dyslipidémies concerneraient en grande majorité l’hypoHDLémie chez les enfants infectés par le VIH sous traitement ARV dans notre structure. Des investigations plus approfondies sont nécessaires pour expliquer leur lien avec le sexe féminin.

# Research article: 156 - Évaluation de la charge virale chez les patients mono-infectés par le virus de l’hépatite B (VHB) dans la cohorte SEN-B au Sénégal

**Published:** 2024-02-23

**Authors:** Mélissa Sandrine PANDI, Messan Kodzo AKOTIA, Adrià RAMÍREZ MENA, Ousseynou NDIAYE, Moussa SEYDI, Gilles WANDELER

**Affiliations:** 1 Centre régional de recherche et de formation à la prise en charge clinique de Fann (CRCF/ANRS), Centre hospitalier national universitaire de Fann, Dakar, Sénégal; 2 Service des maladies infectieuses et tropicales (SMIT), Centre hospitalier national universitaire de Fann, Dakar; 3 Faculté des sciences et techniques (FST), Université Cheikh Anta Diop (UCAD), Dakar; 4 Département des maladies infectieuses, Hôpital universitaire de Berne, Suisse; 5 Haute école des sciences de la santé, Université de Berne, Suisse; 6 Institut de médecine sociale et préventive, Université de Berne, Suisse

## Contexte et objectifs

L’hépatite virale B est une atteinte inflammatoire du foie qui touche plus de 2 milliards de personnes dans le monde. Au Sénégal, 85 % des adultes ont été exposés au virus dont 10 % sont des porteurs chroniques.

Le diagnostic des patients ayant besoin d’un traitement et le contrôle de la suppression de la charge virale du VHB sont des défis dans les pays à revenus faibles et intermédiaires. L’objectif de cette communication est de déterminer la proportion de patients éligibles au traitement antiviral à l’inclusion au sein de la cohorte selon les recommandations de l’Association européenne pour l’étude du foie (EASL) et de déterminer la proportion de patients sous traitement ayant supprimé la charge virale après 12 mois de suivi.

## Méthodes

Il s’agissait d’une étude prospective au sein d’une cohorte incluant des patients mono-infectés par le VHB et naïfs au traitement, suivis au Service des maladies infectieuses et tropicales (SMIT) et au Centre de traitement ambulatoire (CTA) de l’hôpital Fann (Dakar) entre octobre 2019 et novembre 2021. Pour être inclus dans la cohorte il fallait avoir au moins 18 ans, être positif à l’hépatite B et remplir un consentement. Les données précliniques à savoir l’alanine aminotransférase (ALAT), la charge virale et la fibrose ont été étudiées.

Les lignes directrices de l’EASL étaient les suivantes : Cirrhose et charge virale détectable; Fibrose significative et charge virale > 2000 Ul/ml; ALAT > 80 U/L et charge virale > 20 000 Ul/ml; AgHBe positif et âge ≥ 30 ans; Antécédents familiaux de carcinome hépatocellulaire ou de cirrhose. Toutes les analyses statistiques ont été réalisées à l’aide du logiciel RStudio R x64 4.0.1.

## Résultats obtenus

L’étude a été réalisée sur une cohorte de 470 patients constituée de 256 (54,46 %) hommes et de 214 (45,54 %) femmes avec une légère prédominance du sexe masculin (sex-ratio homme/femme de 1,19). La moyenne d’âge constatée était de 32,77 ± 10,30 ans avec des extrêmes allant de 18 à 74 ans.

Selon les critères de l’EASL, 97 patients étaient éligibles au traitement antiviral soit un taux de 20,63 %. Enfin, 12 mois après l’inclusion, la comparaison de la détection de la charge virale entre les patients sous traitement et les non-traités a montré une suppression virologique de 60,74 % chez les patients sous traitement à base de fumarate de ténofovir disoproxil (TDF).

## Conclusion

Cette étude a permis d’identifier 97 patients qui ont été mis sous traitement à base de TDF. Après 12 mois, une suppression virologique de 60,74 % a été observée au sein de la cohorte. D’où l’intérêt de rechercher la cause de l’échec thérapeutique du restant des patients qui se traduit par la non-surpression de leur charge virale.

# Research article: 249 - Des différences générationnelles autour du statut et rôle des HSH au Sénégal qui renforcent des vulnérabilités sociales et sanitaires

**Published:** 2024-02-23

**Authors:** Aissatou SALL, Karim DIOP, Fatim SY, Mame Yacine MBODJ, Cheikh Bamba DIEYE

**Affiliations:** 1 Centre régional de recherche et de formation à la prise en charge clinique de Fann (CRCF), Dakar, Sénégal; 2 Conseil national de lutte contre le sida et les IST (CNLS), Sénégal

## Objectif

La communauté HSH (hommes ayant des relations sexuelles avec d’autres hommes) est composée de différentes catégories d’âge, dont les plus âgés qui ont vécu dans une société sénégalaise tolérante vis-à-vis de certaines attitudes et rôles qui leur étaient assignés. Malgré un contexte socioculturel de répression et de stigmatisation, les plus jeunes récusent ces consensus. L’objectif est de décrire et d’analyser ces différences de perceptions entre les différentes générations et d’analyser leurs effets sur les vulnérabilités auxquels ils sont confrontés.

## Méthode

L’étude a été menée dans le cadre d’un projet sur les populations clés supervisé par le CNLS et financé par le FM. La collecte de données s’est déroulée entre février et juin 2022 dans 8 régions (Dakar, Saint-Louis, Louga, Matam, Kaolack, Kaffrine, Fatick et Tambacounda). Des entretiens individuels (59) et des focus groups (15) ont été effectués auprès des HSH. Au total, 180 HSH ont été interrogés.

## Résultats

La plupart des personnes enquêtées sont de jeunes adultes (36 ont 20-35 ans) et une minorité est plus âgée (14 ont entre 36 et 45 ans,9 ont plus de 46 ans). Les données révèlent des différences de perception entre les plus jeunes et les plus âgés concernant les attitudes, pratiques et rôles liés à leurs vécus. Les plus âgés mettent l’accent sur la discrétion et la dissimulation de l’orientation sexuelle. Ils rapportent qu’auparavant, ils n’affichaient leurs différences que lors des festivités telles que les cérémonies familiales où ils étaient chargés de distraire les invités tout en conseillant les « grandes dames » qui les organisaient. Ils expliquent qu’à l’époque, les pratiques sexuelles étaient tues. Ces attitudes sont généralement récusées par les plus jeunes HSH enquêtés qui rapportent leur frustration d’être exclusivement assignés à de tels rôles. Ils revendiquent une meilleure place dans la société. Certains souhaitent partager leur orientation sexuelle au moins à un membre de leur famille, ou au sein du milieu professionnel, d’autres estiment qu’ils doivent assumer, voire revendiquer publiquement leur différence sexuelle et jouir de leurs droits. L’accès aux réseaux sociaux leur a permis d’être informés des modèles du vécu de leurs pairs dans les pays occidentaux perçus comme des modèles de vie épanouissants. Ils optent généralement pour un style vestimentaire singulier influencé par certains artistes locaux qui les inspirent. Les plus anciens dénoncent également la fréquence des actes sexuels avec contrepartie financière et/ou matérielle pouvant être assimilés à des transactions sexuelles qui les exposent tous. Les personnes enquêtées rapportent toutes sans distinction d’âge la montée de l’homophobie et de diverses formes de violences contre les HSH. Les HSH âgés accusent la nouvelle génération d’être à l’origine de ces rejets et violences, qui les rendent tous plus vulnérables, ce qui crée souvent des rapports hostiles entre eux.

## Conclusion

Dans un contexte marqué par une intolérance sociale accrue vis-à-vis des HSH, des divergences, des désaccords voire des conflits intergénérationnels entre pairs sur l’évolution du statut et l’affirmation de l’orientation sexuelle sont notés et risquent de fragiliser les mécanismes de solidarité et de résilience classiquement assez puissants et protecteurs au sein de la communauté.

# Research article: 71 - Évaluation du test de dépistage Panbio™ Covid-19 Rapid Test Device (Abbott)

**Published:** 2024-02-23

**Authors:** Dianké SAMATÉ, Ousseynou GUÈYE, Adjiratou Aïssatou BA, Halimatou DIOP-NDIAYE

**Affiliations:** Laboratoire de bactériologie et de virologie, Centre hospitalier national universitaire Aristide Le Dantec, Dakar, Sénégal

## Introduction et objectifs

La prise en charge de la pandémie à Covid-19 nécessite un accès le plus large possible au diagnostic. Pour rendre ce diagnostic disponible aux populations en particulier dans les pays à ressources limitées, il est important de disposer de technologies rapides mais fiables et surtout moins onéreuses que la RT-PCR qui est la technique de référence. C’est pour cela que nous avons évalué les performances de détection des tests antigéniques Panbio” Covid-19 Ag Rapid Test Device (Abbott Molecular) sur des prélèvements nasopharyngés et nasaux en comparaison avec la technique de RTPCR Abbott RealTime SARS-CoV-2 Assay (Abbott Molecular).

## Matériel et méthodes

Cette étude a été réalisée au laboratoire de bactériologie et de virologie (LBV) du CHNU Aristide le Dantec où il fallait inclure au moins 150 participants adultes dont le statut pour le SARS-CoV-2 était inconnu et 30 participants dont le test RT-PCR pour le SARS-CoV-2 était positif. Après l’obtention d’un consentement libre et éclairé, 2 prélèvements nasopharyngés (NP) et un prélèvement nasal (NS) ont été réalisés chez chaque participant. Les prélèvements NP et NS ont été testés avec le Panbio Ag et les résultats ont été comparés à ceux de RT-PCR obtenus sur le prélèvement nasopharyngé du patient considéré. L’analyse des performances a porté sur le calcul de la sensibilité (Se), la spécificité (Sp), les valeurs prédictives positives (VPP) et négatives (VPN) et le degré d’accord par le coefficient Kappa de Cohen.

## Résultats

Au total, 179 patients ont été inclus dont 150 participants recrutés parmi les voyageurs fréquentant le laboratoire de bactériologie et de virologie du CHNU Aristide Le Dantec et 29 patients du Centre de traitement de l’hôpital. L’âge moyen était de 33 ans et 40 ans et le sex-ratio de 0,85 et 1,2 respectivement chez les voyageurs et les malades de Covid-19.

Parmi les voyageurs, 21 (20 %), 13 (8 %) et 11 (7,3 %) ont été testés positifs respectivement par RT-PCR, Panbio NP et NS.

## Conclusion

Cette étude a montré une excellente concordance entre le test Panbio et la RT-PCR aussi bien sur des prélèvements NP que NS. Ces bonnes performances et la simplicité d’utilisation du test Panbio confirment l’intérêt de son utilisation dans le diagnostic de la Covid.

# Research article: 165 - Stratégie de dépistage communautaire du virus de l’hépatite B à partir de cas index : exemple du programme « Casamance Research program on HIV-Resistance and Sexual Health » (CARES)

**Published:** 2024-02-23

**Authors:** Benjamin Amaye SAMBOU, Aïssé THIOUBOU, Boubacar DIOUF, Vic ARENDT, Leonard Chabi BINDIA, Sally CAMARA, Martin NATAGNA, Aissatou Marie Louise NDIAGNE

**Affiliations:** 1 ENDA Santé; 2 Hôpital de la Paix, Université Assane Seck, Ziguinchor, Sénégal; 3 Centre hospitalier de Luxembourg, Luxembourg

## Description du problème

L’infection par le virus de l’hépatite B (VHB) constitue un problème de santé publique dans le monde de par sa fréquence et ses complications (cirrhose et cancer du foie). En effet, 2 milliards d’individus présentent une infection avérée par le VHB dont 350 à 400 millions sont atteints d’une hépatite chronique. Le carcinome hépatocellulaire constitue la 3^e^ cause de décès par cancer en Afrique et dans le monde en 2020. La région de Ziguinchor, à l’instar du reste du pays, est une zone de haute endémicité pour le VHB. La baisse de la prévalence et la prévention des complications reposent sur un dépistage de la population et une prise en charge précoce des cas positifs. Cette étude avait pour but de dépister et de déterminer la prévalence du VHB dans l’entourage de patients suivis pour un portage chronique du VHB dans le cadre de la cohorte Casamance Research program on HIV-Resistance and Sexual Health (CARES).

## Activités réalisées et résultats obtenus

Dans le cadre du programme CARES, des tests ciblés du virus de l’hépatite B ont été réalisés d’octobre 2019 à juin 2022. Ce dépistage a été effectué chez 18 737 personnes et 1334 sont revenus positifs et enrôlés dans l’étude. À l’inclusion, un dépistage de l’entourage est proposé à chaque patient. Une équipe (assistant social, biologiste, chargé de suivi évaluation) se déplace vers ces familles pour fournir des informations sur la maladie et proposer le dépistage du VHB par des tests rapides. Quarante familles de patients AgHBs+ suivis dans la cohorte ont été visitées. Cent soixante-treize personnes ont bénéficié d’un dépistage avec en moyenne 4,3 membres par famille [1-15]. Il s’agissait de 75 hommes et 98 femmes soit un sex-ratio de 0,76. Leur âge moyen était de 28 ans avec des extrêmes de 2 et 80 ans. Douze tests (7 hommes et 5 femmes) sont revenus positifs soit un taux de séropositivité de 7 %. Ils étaient retrouvés dans sept familles soit une prévalence de 1,7 cas par cas index [1-4]. Il s’agissait d’apparentés au premier degré dans 50 % des cas.

## Leçons apprises et étapes futures

La mise en œuvre du projet CARES a permis d’expérimenter la stratégie de dépistage du virus de l’hépatite B à partir des cas index. Ceci pourrait être un moyen de prévention du carcinome hépatocellulaire chez les porteurs chroniques du VHB. Cependant pour qu’elle soit plus efficace il faudrait, en plus de l’AgHBs, rechercher les anticorps anti-HBs et anti-HBc pour mieux déterminer le profil des personnes dépistées.

# Research article: 14 - Impact de la classe du 3^e^ médicament actif du régime antirétroviral des personnes vivant avec le VIH sur le ratio CD4/CD8 : résultats de l’étude de la cohorte québécoise sur le VIH

**Published:** 2024-02-23

**Authors:** Mohamed N’dongo SANGARÉ, Jean-Guy BARIL, Alexandra DE POKOMANDY, Marina KLEIN, Réjean THOMAS, Cécile TREMBLAY, Costa PEXOS, Madeleine DURAND, Louise LAPORTE, Helen TROTTIER

**Affiliations:** 1 Université du Québec en Abitibi-Témiscamingue, Rouyn-Noranda, Canada; 2 Clinique de médecine urbaine du Quartier latin, Montréal, Faculté de médecine, Université de Montréal, Centre hospitalier de l’Université de Montréal, Canada; 3 Service des maladies virales chroniques, Département de médecine familiale, Université de McGill, Montréal; 4 Service des maladies virales chroniques, Université de McGill, Montréal; 5 Clinique médicale L’Actuel, Montréal; 6 Faculté de médecine, Centre hospitalier de l’Université de Montréal, Département de microbiologie, maladies infectieuses et immunologie, Université de Montréal; 7 Département de microbiologie, maladies infectieuses et immunologie, Université de Montréal; 8 Centre hospitalier universitaire de Sainte-Justine, Montréal; 9 Département de médecine sociale et préventive, Centre hospitalier universitaire de Sainte-Justine, Montréal

## Introduction

L’impact des différentes classes thérapeutiques de médicaments dans la thérapie antirétrovirale (TAR) sur le ratio CD4/CD8 n’est pas bien documenté chez les personnes traitées pour l’infection pour le VIH. L’objectif de cette étude était d’analyser l’effet à long terme de l’exposition aux inhibiteurs de l’intégrase (INI) sur le ratio CD4/CD8 par rapport aux inhibiteurs non nucléosidiques de la transcriptase inverse (INNTI) ou aux inhibiteurs de la protéase (IP) chez les personnes vivant avec le VIH (PvVIH) traitées avec les TAR incluant deux inhibiteurs nucléosidiques de la transcriptase inverse (INTI).

## Méthodes

Nous avons utilisé les données de la cohorte VIH du Québec incluant un suivi jusqu’en août 2017. Notre étude inclut tous les patients de la cohorte ayant reçu une TAR composée de deux INTI et d’un troisième médicament actif d’une classe différente (INNTI, IP ou INI) depuis le 1^er^ janvier 2006, pendant au moins 16 semaines. Une analyse à l’aide d’un modèle structurel marginal de Cox a été réalisée pour estimer l’effet des classes thérapeutiques sur le ratio CD4/CD8 en tenant compte de trois cut-offs différents (≥ 1, ≥ 0,8 et ≥ 1,2).

## Résultat

Parmi les 3907 patients éligibles, 972 (24,9 %), 1996 (51,1 %) et 939 (24,0 %) ont été respectivement exposés à une TAR dont le 3^e^ agent actif était soit un INNTI, un IP ou un INI. La durée totale de suivi après l’inclusion était de 13 640, 24 années-personnes et 1790 patients ont atteint un ratio CD4/CD8 ≥ 1. L’incidence cumulée du ratio CD4/CD8 ≥ 1 était de 13,1 % (intervalle de confiance (IC) à 95 % : 12,5 %-13,7 %). Le hazard ratio (HR) pondéré pour l’association entre la 3^e^ classe thérapeutique active et le ratio CD4/CD8 ≥ 1 était de 0,54 (IC 95 % : 0,47-0,62) pour les patients exposés aux INNTI + 2 INTI et de 0,39 (IC 95% : 0,34-0,45) pour les personnes exposées aux IP + 2 INTI, par rapport à celles exposées aux INI + 2 INTI.

## Conclusion

Nos résultats ont montré que chez les personnes traitées pour le VIH, une TAR à base d’INI amène une plus forte incidence de la normalisation du ratio CD4/CD8 par rapport à une TAR à base d’INNTI ou d’IP.

# Research article: 136 - Cartographie de la prévalence des hépatites B et C chez les femmes enceintes au Sénégal

**Published:** 2024-02-23

**Authors:** Abou Abdallah Malick DIOUARA, Sarbanding SANÉ, Habibou SARR, Fatou THIAM, Halimatou DIOP-NDIAYE, Seynabou LO, Fodé DANFAKHA, Marie Edouard FAYE DIÉMÉ, Babacar BIAYE, Coumba TOURÉ KANE, Martine PEETERS, Ahidjo AYOUBA

**Affiliations:** 1 Groupe de recherche Biotechnologies appliquées & Bioprocédés environnementaux (GRBA-BE), École supérieure polytechnique (ESP), Université Cheikh Anta Diop, Dakar, Sénégal; 2 Service des maladies infectieuses, Hôpital de la Paix, Ziguinchor, Sénégal; 3 Unité de formation et de recherche en sciences de la santé, Université Assane Seck, Ziguinchor; 4 Laboratoire de bactériologie et de virologie, Centre hospitalier national universitaire Aristide Le Dantec, Université Cheikh Anta Diop, Dakar; 5 Laboratoire de bactériologie et de virologie, Centre hospitalier régional de Saint-Louis, Sénégal; 6 District sanitaire-région médicale de Kédougou, Kédougou, Sénégal; 7 Clinique gynécologique et obstétricale, Laboratoire de bactériologie et de virologie, Centre hospitalier national universitaire Aristide Le Dantec, Université Cheikh Anta Diop, Dakar; 8 Centre de santé Gaspard Kamara, Dakar; 9 Institut de recherche en santé, de surveillance épidémiologique et de formation (IRESSEF), Sénégal; 10 Université du Sine Saloum El Hâdj Ibrahima Niass (USSEIN), Sénégal; 11 TransVIHMI (Recherches translationnelles sur le VIH et les maladies infectieuses), Université de Montpellier/INSERM U1175, Institut de recherche pour le développement (IRD), France

## Objectif

L’objectif de cette étude était de documenter la prévalence de l’hépatite B (VHB) et C (VHC) chez les femmes enceintes au Sénégal.

## Méthodes

Nous avons exploité les reliquats d’échantillons de plasma entreposés à – 80 °C dans la biobanque du GRBA-BE et les métadonnées associées, collectés dans le cadre du projet EPIVHE, une enquête séro-épidémiologique de l’hépatite E conduite auprès des femmes enceintes sur la période allant de mars à juillet 2021.

Les échantillons ont été soumis à des tests rapides d’orientation diagnostique pour la détection de l’antigène HBs (AgHBs) d’une part et d’autre part les anticorps anti-VHC. Les kits utilisés étaient respectivement HB-sAg Serum cassette MEDIFF (Aubagne, France) et Wantai Rapid Test for Antibody to Hepatitis C (Wantai Biological Pharmacy Enterprise, Beijing), le tout conformément aux instructions des fabricants.

Pour évaluer les facteurs associés aux prévalences, des analyses bivariées ont été effectuées avec le logiciel JMP’ Pro version 15.0.0 (SAS Institute Inc., Cary, Caroline du Nord, 1989-2021). Pour des fréquences inférieures à 5, des tests de chi2 ou de Fischer ont été réalisés. Dans tous les cas, l’intervalle de confiance était fixé à 0,95 et les valeurs de p considérées significatives si < 0,05.

## Résultats obtenus

Au total, 1227 femmes enceintes étaient recrutées dans les villes de Dakar (n = 156), Saint-Louis (n = 400), Kédougou (n = 397) et Ziguinchor (n = 264). La médiane d’âge était de 25 ans [18-50]. La distribution par tranche d’âge était de 43 %, 29,1 % et 18,2 % respectivement pour les [18-23], [24-29] et [30-35] ans. Les femmes d’âge ≥ 36 ans ne représentaient que 9,7 %; parmi elles 3,6 % (n = 45) avaient plus de 40 ans. S’agissant du niveau d’instruction, 31.7 % d’entre elles n’en avaient pas. Le taux de non-instruites était plus élevé à Kédougou (58,7 %). Seulement 9 % des participantes avaient atteint un niveau d’études supérieures. Contrairement aux autres localités, 25,9 % des femmes de Dakar avaient effectué des études supérieures. Globalement, seules 18.7 % des participantes avaient des revenus réguliers.

Globalement, le portage du virus de l’hépatite B était de 7,3 % et variait d’une localité à l’autre avec des prévalences de 9,1 % à Ziguinchor, 8,8 % à Saint-Louis, 6,5 % à Kédougou et 3 % à Dakar. Par ailleurs, la prévalence de l’hépatite C était de 0,2 %. À noter qu’aucun cas n’a été décelé chez les participantes de Saint-Louis contrairement à Dakar, Ziguinchor et Kédougou où les prévalences étaient respectivement de 0,6 %, 0,4 % et 0,3 %. Aucune co-infection VHB et VHC n’a été décelée. Malgré les variations observées, l’analyse des prévalences selon la localité, la tranche d’âge, le niveau d’instruction, le statut matrimonial et professionnel ne montrait aucune différence statistiquement significative.

## Conclusion

Cette étude a révélé une prévalence élevée de l’hépatite B chez les femmes enceintes (7,3 %) même si elle reste inférieure à celle observée dans la population générale (11 %). Par ailleurs, la prévalence de l’hépatite C (0,2 %) est inférieure à celle trouvée chez les patients hémodialysés (5,6 %). Par conséquent, d’autres études complémentaires sont requises pour évaluer le risque de transmission mère-enfant, et déterminer le caractère aigu ou chronique des infections décelées y compris les génotypes viraux.

# Research article: 254 - Gestion de l’infection à Covid-19 à l’hôpital Dalal Jamm : de la première à la quatrième vague

**Published:** 2024-02-23

**Authors:** Ndeye Amy SARR, Khady FALL, Ibrahima GAYE, Aly SALANE, Angeline NIEBA, Ndeye Aissatou LAKHE, Papa Samba BA, Abibatou SALL, Fatou CISSÉ, Fatou Samba NDlAYE, Moussa SEYDI, Louise FORTES

**Affiliations:** 1 Hôpital Dalal Jamm, Dakar, Sénégal; 2 Service des maladies infectieuses et tropicales (SMIT), Centre hospitalier national universitaire de Fann, Dakar; 3 Hôpital principal de Dakar

## Introduction

L’hôpital Dalal Jamm assure la prise en charge de la Covid-19 depuis le 27 mars 2020. Il abrite l’un des plus grands Centres de traitement des épidémies (CTE) du Sénégal avec une capacité de 211 lits lors de la première vague. L’objectif de ce travail était de décrire le dispositif de prise en charge mis en place dans l’hôpital ainsi que les caractéristiques des patients admis pour infection à Covid-19.

## Méthodologie

Il s’agit d’une étude transversale menée sur la période du 13 décembre 2020 au 5 janvier 2022. Ont été inclus tous les patients admis au CTE en dehors des données de la réanimation. Les données ont été recueillies à partir des registres, des dossiers médicaux et des rapports de prise en charge. L’analyse a été faite grâce au logiciel SPSS.

## Résultats

Le CTE a pris en charge un nombre total de 2977 patients. L’âge moyen des patients était de 52,4 ± 20,0 ans avec des extrêmes de 6 jours et 104 ans. Plus d’un tiers des patients (39,5 %) avaient plus de 60 ans et 6 % moins de 18 ans. Il y avait une légère prédominance masculine (sex-ratio : 1,1). Près de la moitié (45 %) des malades présentaient des co-morbidités dominées par l’hypertension artérielle (701 cas, 24 %) et le diabète (622 cas, 21 %). La durée médiane des symptômes avant l’admission était de 7 jours. Sur les 2977 patients, 1109 (37 %) présentaient des formes modérées à critiques. Plus de la moitié des patients (55,4 %) ont bénéficié d’un traitement médical (antibiotique, anticoagulant, corticoïdes). Soixantedix pour cent des 685 patients avaient plus de 25 % de lésions au scanner thoracique. Au plan évolutif, le taux de guérison était de 89,4 % et la létalité de 9,6 % (285 cas). L’âge de plus 60 ans et la sévérité du tableau clinique étaient associés au décès. La prise en charge des patients au CTE a été possible grâce à la mobilisation d’une équipe multidisciplinaire. Sur le plan psycho-social, les patients et leurs parents ont bénéficié d’un accompagnement psychologique avec une salle de jeu qui a été mise en place en zone rouge lors de la première vague.

## Conclusion

La gestion de la Covid-19 au CTE de Dalal Jamm a été faite par une équipe multidisciplinaire ayant permis une prise en charge des patients quel que soit le tableau clinique. Le profil des patients a évolué durant les trois vagues et les décès ont concerné principalement les sujets âgés présentant des co-morbidités.

# Research article: 271 - TEST m-PIMA HIV1/2 VL : une solution pour le suivi de la charge virale des patients vivant avec le VIH-2

**Published:** 2024-02-23

**Authors:** Pauline Yacine SENE, Halimatou DIOP-NDIAYE, Brianan KIERNAN, Khadidiatou COULIBALY, Makhtar CAMARA, Cheikh Tidiane NDOUR, Coumba TOURÉ KANE

**Affiliations:** 1 Laboratoire de bactériologie et de virologie, Centre hospitalier national universitaire Aristide Le Dantec, Université Cheikh Anta Diop, Dakar, Sénégal; 2 Clinton Health Access Initiative, Dakar; 3 Division de lutte contre le sida et les IST (DLSI), Ministère de la santé, Dakar; 4 Institut de recherche en santé, de surveillance épidémiologique et de formation (IRESSEF), Dakar

## Contexte

Le suivi virologique de l’infection à VIH-2 reste problématique dans la plupart des pays de l’Afrique de l’Ouest du fait d’un nombre limité de tests de charge virale disponibles. L’arrivée du test m-PIMA HIV1/2 VL apparaît comme une bonne alternative pour la mesure de la charge virale.

L’objectif de cette étude était d’évaluer les performances du test m-PIMA HIV1/2 VL en comparaison avec les techniques de référence Generic HIV-2 Charge virale (Biocentric) et la technique maison HIV-2 Mvp (Max von Pettenkofer Institute (MvP), université Ludwig-Maximilian de Munich).

## Méthode

Un prélèvement de sang veineux a été obtenu chez 90 patients positifs au VIH2 et naïfs de tout traitement antirétroviral dans cette étude. La charge virale (CV) plasmatique a été réalisée avec les techniques : m-PIMA VIH1/2 VL (50 pL), Generic HIV2 Charge Virale (500 pL) et la technique maison HIV-2 Mvp (500 pL). La comparaison entre les techniques a été évaluée après conversion des valeurs de CV en échelle logarithmique (différence logarithmique significative si Delta Log CV > ±0,5 log 10). Les performances ont été mesurées grâce au calcul de la sensibilité et de la spécificité au seuil de détectabilité du test m-PIMA VIH1/2 VL (2,9 log_10_ copies/ml) et au seuil d’échec virologique (3,0 log_10_ copies/ml). Le coefficient de corrélation de Pearson et la concordance de Blant-Altman ont été obtenus avec le logiciel MethVal.

## Résultats

La concordance globale m-PIMA/Generic HIV-2 était respectivement de 97,7 % (88 échantillons concordants et 2 échantillons discordants dont les résultats étaient surestimés par le test m-PIMA) et de 95,5 % (86 échantillons concordants et 4 échantillons discordants avec 3 résultats surestimés par le test m-PIMA) à 2,9 log_10_ copies/ml et à 3 log_10_ copies/ml. La sensibilité et la spécificité étaient de 100 % et de 95,8 % à 2,9 log_10_ copies/ml, et de 97,6 % et 93,9 % à 3 llog_10_ copies/ml. Un coefficient de corrélation (r = 0,950) a été trouvé entre les deux techniques avec une différence moyenne de 0,229 log_10_ copies/ml [IC à 95 % de 0,161 à 0,297 log_10_ copies/ml]. La concordance globale m-PIMA/Mvp était respectivement de 98,8 % (89 échantillons concordants et 1 échantillon discordant dont le résultat était surestimé par le test m-PIMA) et de 97,7 % (88 échantillons concordants et 2 échantillons discordants avec 1 résultat surestimé par le test m-PIMA) à 2,9 log_10_ copies/ml et à 3 log_10_ copies/ml. La sensibilité et la spécificité étaient de 97,7 % et de 100 % à 2,9 log_10_ copies/ml, et de 97,7 % et 97,9 % à 3 log10 copies/ml. Un coefficient de corrélation (r = 0,979) a été trouvé entre les deux techniques avec une différence moyenne de 0,121 log_10_ copies/ml [IC à 95 % de 0,077 à 0,165 log_10_ copies/ml].

## Conclusion

Ces résultats montrent que le Point-of-Care m-PIMA VIH1/2 VL offre d’excellentes performances en comparaison avec les tests de référence Generic HIV-2 Biocentric et la technique maison HIV-2 Mvp (Max von Pettenkofer Institute (MvP) université Ludwig-Maximilian de Munich). Le test m-PIMA VIH1/2 VL pourrait être utilisé pour la surveillance de la charge virale du VIH-2 au Sénégal.

# Research article: 35 - Cinétique d’évolution des anticorps anti-SARS-CoV-2 après la vaccination contre la Covid-19 chez des volontaires Sénégalais

**Published:** 2024-02-23

**Authors:** Larry SEYE, Nalda DEBSIKREO, Djibril WADE, Tandakha Ndiaye DIÈYE, Nda Kouame Nazaire KOUADIO, Flore Biscotine MAKOUTCHOUANG NZONDE, Joséphine Khady BADIANE

**Affiliations:** 1 Institut de recherche en santé, de surveillance épidémiologique et de formation (IRESSEF), Sénégal; 2 Centre national de transfusion sanguine (CNTS), Sénégal; 3 Centre hospitalier national universitaire Aristide Le Dantec, Dakar, Sénégal

## Contexte et objectif

La maladie à coronavirus 2019 (Covid-19) a touché plus de 595 millions de personnes dans le monde et causé plus de 6 millions de décès. Très rapidement, de nombreux vaccins ont été développés pour s’ajouter à l’arsenal de prévention et de lutte contre la propagation du virus. Au Sénégal, les vaccins de Sinopharm et d’AstraZeneca ont été les premiers à être introduits et utilisés, suivis de ceux de Johnson & Johnson et plus récemment celui de Pfizer BioNTech. Peu de données étaient disponibles en Afrique sur le pouvoir protecteur de ces vaccins ainsi que sur la durabilité de leur réponse. C’est ainsi que notre étude s’était fixé comme objectif de déterminer la cinétique d’évolution des anticorps anti-SARS-CoV-2 après la vaccination contre la Covid-19 chez des volontaires au Sénégal.

## Méthodes

Nous avions procédé à une étude observationnelle chez des personnes vaccinées avec AstraZeneca ou Sinopharm. Chaque volontaire avait deux points de prélèvement : 3 mois puis 10 mois après la 2^e^ dose pour ceux vaccinés avec Sinopharm; et 3 mois après la 1^re^ dose (le jour de la 2^e^ dose) puis à 10 mois après la 2^e^ dose pour ceux vaccinés avec AstraZeneca. Un prélèvement sanguin a été réalisé pour recueillir le plasma afin de procéder au dosage immuno-enzymatique (ELISA) des anticorps anti-SARSCoV-2 pour les sous-unités S1 et S2 du gène S en utilisant le kit de Quansys Biosciences. Les taux d’anticorps ont été groupés par sexe, âge et type de vaccin reçu. Les tests non paramétriques de Chi carré et de Wilcoxon-Mann-Whitney ont été utilisés pour la comparaison des taux d’anticorps des différents groupes.

## Résultats obtenus

L’étude a porté sur 45 volontaires composés de 25 vaccinés avec Sinopharm et 20 vaccinés avec AstraZeneca. Globalement, nos résultats ont montré une augmentation significative du taux d’anticorps anti-SARS-CoV-2 aussi bien pour la protéine S1 (p = 0,003) que pour la protéine S2 (p = 0,02) sur la période de 3 à 10 mois. À 10 mois après la 2^e^ dose, ceux vaccinés avec Sinopharm avaient des taux d’anticorps anti-S2 plus élevés que ceux vaccinés avec AstraZeneca (p = 0,05) alors que les différences par rapport aux taux d’anticorps anti-S1 n’étaient pas significatives. Ceux vaccinés avec Sinopharm avaient tous des taux de positivité en anticorps anti-SARS-CoV-2 de 100 % aussi bien à 3 mois qu’à 10 mois après la 2^e^ dose. Pour ceux vaccinés avec AstraZeneca, 90 % (18/20) avaient des taux de positivité en anticorps à 3 mois après la 1^re^ dose et 95 % (19/20) à 10 mois après la 2^e^ dose. Il n’y avait pas de différence significative entre hommes et femmes ni entre les sujets âgés de plus de 40 ans et ceux de moins de 40 ans.

## Conclusion

Nos résultats ont montré la persistance des réponses anticorps dans la quasitotalité des volontaires jusqu’à 10 mois après la 2^e^ dose aussi bien avec Sinopharm qu’avec AstraZeneca.

# Research article: 162 - Riposte communautaire face à la Covid-19 : contribution d’ENDA Santé à la vaccination à travers la clinique mobile dans la région de Dakar

**Published:** 2024-02-23

**Authors:** Fatou Kiné SOGODOGO, Dieynaba MBAYE, Sidy Mokhtar NDIAYE, Ibrahima BÂ, Mouhamadou Siradi BÂ, Nguissali TURPIN

**Affiliations:** ENDA Santé

## Contexte et objectif

À l’image des autres pays, le Sénégal a connu une augmentation des cas de Covid-19 durant la première et la deuxième vague entraînant plusieurs décès. C’est ainsi que la vaccination a été proposée par l’OMS en vue d’éviter la forme sévère de la maladie. Pour mieux maîtriser la pandémie, une campagne de vaccination nationale a été initiée par le Ministère de la santé et de l’action sociale. ENDA Santé en partenariat avec Bioforce avait mis en place un projet « Dispositif de surveillance, de réponse communautaire, d’atténuation de l’impact de la Covid-19 », afin de contribuer à l’effort de la riposte communautaire. L’objectif était d’appuyer le district de Yoff dans sa campagne de vaccination contre la Covid-19 et de contribuer à la riposte contre la pandémie dans les quartiers de Yoff, Ouakam, Nord Foire et environs.

## Activités réalisées et résultats obtenus

ENDA Santé avait déployé ses cliniques mobiles à Dakar, notamment dans le district Ouest, pour apporter sa contribution à la campagne de vaccination contre la Covid-19. La clinique mobile est un minibus médicalisé et doté d’intrants médicaux (gants, masques, gel, vaccins, coton, seringues) fournis par le district ainsi que d’une équipe médicale composée d’une sage-femme, d’un chargé du contrôle et du renouvellement du stock de médicaments, d’un chauffeur et d’un animateur de terrain. Les responsables de districts identifient les localités dans lesquelles le taux de vaccination est jugé faible. Des stratégies mobiles facilitées par les Bajenu gox (marraine de quartier) ont été organisées. Des porte-à-porte sont faits pour informer et sensibiliser les gens, surtout les personnes âgées, à se faire vacciner.

Cette démarche a permis de booster la vaccination chez les populations des quartiers ne disposant pas de structure sanitaire de proximité et où les populations n’ont pas assez d’informations sur le vaccin.

9 journées de campagnes de vaccination ont été organisées. Cette stratégie avancée de vaccination a coïncidé avec la disponibilité des vaccins Sinopharm et Johnson & Johnson.

Environ 1890 personnes ont été vaccinées dans la clinique mobile dont 976 hommes et 914 femmes. 75 % des personnes vaccinées dans la clinique avaient 50 ans ou plus.

## Leçons apprises et étapes futures

La campagne de vaccination de proximité accompagnée d’activités de sensibilisation a permis de contribuer à l’amélioration de l’accès des populations à la vaccination et au respect des gestes barrières dans des quartiers ne disposant pas de structures sanitaires de proximité.

# Research article: 251 - Le coaching par les pairs, une expérience innovante de relèvement de la performance des médiateurs

**Published:** 2024-02-23

**Authors:** Ameth SOUGOU, Barra Lamine FALL ANCS

**Affiliations:** (Alliance nationale des communautés pour la santé), Dakar, Sénégal

## Introduction

Les résultats probants du projet PEPFAR (plan d’urgence du Président américain pour la lutte contre le sida) durant la première année fiscale ont été à l’origine de l’extension de la stratégie dans le plan opérationnel régional 2020 avec comme conséquence l’enrôlement de nouveaux sites, à Dakar par exemple : les districts de Mbao, Keur Massar et Nord.

En dépit de cette performance qui a valu cette extension, on note des résultats assez faibles en ce qui concerne la cible des professionnelles du sexe (PS) dans certains sites, notamment les districts de Keur Massar, Mbao, Nord, Institut d’hygiène sociale. Les ateliers de formation, bien qu’importants, n’ont pas pu régler la situation.

Néanmoins, 3 médiatrices se sont remarquablement distinguées par leurs méthodes utilisées sur le terrain. C’est dans ce cadre que s’inscrit cette initiative pour permettre un partage d’expériences afin de faciliter le réajustement des autres médiatrices.

## Objectifs

Objectif général : contribuer à la performance des médiatrices PS à travers un partage d’expériences.

Objectifs spécifiques :

partager les stratégies pour un bon ciblage sur le terrain avec les médiatrices PS;

partager les techniques pour améliorer la performance des médiatrices PS au niveau des sites;

échanger sur les techniques d’utilisation des réseaux sociaux et sur les lieux de rencontre des PS clandestines pour mieux toucher la cible.

Méthodologie.

Analyse des données.

Visite des sites (5 jours passés en moyenne dans chaque site).

Sorties dans les points chauds.

Partage d’expériences.

Médiateurs coachés : médiatrices PS et médiatrices sur site.

Sites investis : sites PEPFAR de Keur Massar, Mbao, Nord, IHS.

Période : du 5 juillet au 14 août 2021.

Résultats.

État des lieux :

analyse SWOT des interventions communautaires des médiateurs;

détermination des limites des médiateurs; découverte de points chauds passés souvent inaperçus.

Partage des stratégies pour un bon ciblage sur le terrain avec les médiatrices PS.

Partage des techniques pour améliorer la performance des médiatrices PS au niveau des sites.

Échanges sur les réseaux sociaux et lieux de rencontre des PS clandestines pour mieux toucher la cible.

Coaching des médiatrices au niveau des districts (DS Mbao : 5; DS Keur Massar : 6; DS Sud : 4; DS Nord : 7; DS Pikine : 4).

## Conclusion

Le coaching par les pairs a permis aux médiatrices d’avoir une autre idée de ce qui pourrait être considéré comme un point chaud. Il a sans nul doute constitué une expérience innovante qui a permis à ces médiatrices de découvrir de nouvelles approches favorisant leurs interventions dans les points chauds les plus difficiles d’accès, relevant ainsi la performance de beaucoup d’entre elles.

# Research article: 243 - Au Sénégal, les médias « arbitres » dans le jeu d’une « démocratie sanitaire » sous tutelle politique ?

**Published:** 2024-02-23

**Authors:** Khoudia SOW, Alice DESCLAUX, Mame Yacine MBODJ

**Affiliations:** 1 Centre régional de recherche et de formation à la prise en charge clinique de Fann (CRCF), Dakar, Sénégal; 2 TransVIHMI, Université de Montpellier, INSERM, IRD, Montpellier, France

## Objectifs

L’épidémie de Covid a été marquée par une intense communication avec des prises de parole fréquentes au plus haut niveau politique, qui a donné le ton d’un traitement politique de l’épidémie. L’objectif est d’analyser le rôle et la place des médias durant cette période.

## Méthodologie

Dans le cadre du projet CORAF (Coronavirus Anthropologie Afrique) développé au sein du programme ARIACOV (Appui à la riposte africaine contre la Covid, financé par l’AFD/IRD), un dispositif de veille des médias et opinions a collecté quotidiennement, de février 2020 à octobre 2021, des articles de presse écrite ou en ligne ayant trait à la Covid en intégrant les commentaires des lecteurs ainsi que les informations circulant sur les réseaux sociaux (infox incluses). Une recension des mesures de santé publique, des entretiens semi-directifs avec des professionnels de santé, des informateurs clés et des observations dans des structures de santé ou lors de diverses manifestations en lien avec la Covid ont été effectués.

## Résultats

Les autorités ont organisé une intense communication institutionnelle marquée par le primat des approches biomédicales ou politiques et la valorisation des initiatives gouvernementales. Leur promotion des attitudes préventives a été réalisée parfois de manière assez autoritaire, voire culpabilisante. En plus de vulgariser les informations scientifiques, de diffuser, commenter les données épidémiques journalières, de relayer les messages de prévention, et de diffuser les expériences du vécu de la Covid-19, les médias ont donné leur point de vue sur la gestion de l’épidémie ou relayé celui de citoyens et de divers spécialistes en santé qui installent une « actualité épidémique ». En l’absence d’un véritable comité scientifique émettant publiquement des recommandations, c’est à travers les médias que des positions techniques et scientifiques adverses sont défendues à la fois par des experts en santé publique et par des citoyens qui s’expriment pour expliquer, commenter, approuver ou contester la riposte. Même si ces échanges sont susceptibles de renforcer la confusion dans un environnement marqué par les incertitudes, les changements et fluctuations des instructions et par la diffusion d’infox de toutes origines, les populations, qui se méfient parfois des informations délivrées par les autorités, apprécient la possibilité de disposer d’autres sources de connaissances à travers ces contributions à la fois citoyennes et scientifiques. De plus, la diffusion de ces informations ou débats dans les réseaux sociaux leur permet de donner leurs avis ou opinions et de partager leurs propres expériences autour de la Covid-19.

## Conclusion

En publiant les commentaires, les médias, notamment ceux en ligne, ont permis l’existence d’un espace d’expression, première étape d’une forme de « démocratie sanitaire » inédite. Les contributions d’experts, les avis de citoyens exprimés à travers ces espaces médiatiques se sont mués parfois en instruments de veille, d’alerte, d’évaluation voire de « tribunal » de la riposte à la Covid-19. Ces expressions ont probablement influé sur les décisions et orientations stratégiques. Des dispositifs de consultation des citoyens formels et ouverts à toutes les catégories sociales devraient être développés dans la gestion des crises sanitaires, encore dominée par le biomédical et le politique.

# Research article: 241 - Points forts, défis et leçons apprises de la Covid pour la préparation des futures épidémies

**Published:** 2024-02-23

**Authors:** Khoudia SOW, Alice DESCLAUX

**Affiliations:** 1 Centre régional de recherche et de formation à la prise en charge clinique de Fann (CRCF), Dakar, Sénégal; 2 UMI233, TransVIHMI, Université de Montpellier, INSERM, IRD, Montpellier, France

## Objectifs

Dans un contexte marqué par les effets délétères de la Covid sur les systèmes de santé fragiles des pays africains, les stratégies de préparation ou « preparedness » deviennent primordiales face aux risques d’émergence de nouvelles épidémies. L’objectif de cette communication est de partager les analyses issues de l’expérience d’acteurs ayant été impliqués dans la lutte contre la Covid au niveau des structures de coordination sous-régionales en Afrique et au Sénégal. Les points forts, les défis et les priorités au niveau régional et national pour renforcer les dispositifs de préparation des épidémies vont être partagés.

## Méthodologie

Dans le cadre du Projet Pandemic Preparedness (PPP) qui analyse la préparation des épidémies au niveau régional, national et global, une série d’entretiens a été menée entre juillet et septembre 2022 auprès de 12 responsables d’organisations sanitaires internationales basés en Afrique subsaharienne, d’ONG, de structures de gestion des urgences sanitaires, de laboratoires et de structures de recherche impliqués dans la lutte contre la Covid. Deux focus groups ont été réalisés auprès de leaders d’organisations communautaires et de professionnels de la santé au Sénégal.

## Résultats

Pour les points forts, les acteurs interrogés rapportent que la plupart des pays ont une tradition de programmes de lutte contre les épidémies (VIH, Ébola…) et de réponse aux urgences de santé publique liées aux maladies infectieuses, qui a facilité la mise en place rapide des mesures de riposte impliquant les hautes autorités politiques. Ils ont salué la disponibilité de centres d’opérations des urgences sanitaires, du dispositif de santé au niveau décentralisé, et le renforcement des équipements sanitaires. Ils ont apprécié la résilience des communautés qui ont souvent participé au financement de la réponse, ainsi que l’utilisation des nouvelles technologies.

Les personnes interrogées ont déploré de nombreux défis tels que : une gouvernance et un modèle de réponse politisés, médicalisés et centralisés « top down » souvent en déphasage avec les réalités du terrain; les difficultés d’application du RSI (Règlement sanitaire international); et l’insuffisance de l’application des leçons apprises de la gestion des épidémies de VIH et d’Ébola. Le manque de dispositifs sanitaires pour la détection et la prise en charge des cas au début de l’épidémie a été rappelé. Les acteurs ont également mis l’accent sur les limites d’une communication peu adaptée aux réalités socio-culturelles, brouillée par les effets délétères de l’infodémie et par l’insuffisance du recours à l’expertise communautaire. Selon eux, les campagnes de vaccination n’ont pas pu vaincre les réticences de la majorité de la population africaine. Ils déplorent les difficultés de la circulation des informations et de la coordination des réponses entre les pays.

## Conclusion

Les personnes enquêtées ont rappelé la nécessité de maintenir la santé comme priorité de l’agenda du développement, humanitaire et sécuritaire. Elles proposent également de repenser le rôle de l’OMS, d’adapter le RSI, de renforcer l’appui et la coordination des réponses régionales en valorisant les approches communautaires et de relancer la vaccination à partir des réalités épidémiologiques et sociales africaines basées sur l’évaluation critique de la riposte à la Covid.

# Research article: 240 - Des transgenres vulnérables exposés précocement à de multiples violences au Sénégal

**Published:** 2024-02-23

**Authors:** Khoudia SOW, Cheikh Bamba DIEYE, Mame Yacine MBODJ, Ibrahima SYLLA, Cyrille BASSÈNE

**Affiliations:** 1 Centre régional de recherche et de formation à la prise en charge clinique de Fann (CRCF), Dakar, Sénégal; 2 Conseil national de lutte contre le sida (CNLS), Sénégal; 3 Division de lutte contre le sida et les IST (DLSI), Ministère de la santé, Sénégal

## Contexte et objectifs

Les populations clés au Sénégal sont confrontées à une vulnérabilité accrue au VIH marquée par la stigmatisation sociale. Parmi elles, la catégorie des transgenres est peu connue. L’objectif est de proposer quelques principaux critères qui permettent d’identifier des « transgenres » au sein de la communauté des hommes ayant des relations sexuelles avec d’autres hommes (HSH), de décrire leur vécu et d’analyser les facteurs de vulnérabilité liés au VIH.

## Méthodes

Une enquête qualitative exploratoire a été menée dans le cadre d’un projet sur les populations clés supervisé par le CNLS et financé par le FM. La collecte de données s’est déroulée entre février et juin 2022 dans 8 régions (Dakar, Saint-Louis, Louga, Matam, Kaolack, Kaffrine, Fatick et Tambacounda). 59 entretiens individuels ont été effectués auprès des populations clés, 10 entretiens individuels avec le personnel de santé et 15 focus groups. Au total, 180 HSH ont été enquêtés dont 24 transgenres.

## Résultats

L’analyse des données a permis d’identifier quelques critères permettant de classer la catégorie de femme transgenre : avoir éprouvé dès l’enfance le sentiment d’être une femme, tendance naturelle à exprimer sa féminité, ne pas être attiré par les relations amoureuses avec des femmes, être sexuellement exclusivement « passif » ou plus à l’aise avec des relations passives, avoir envie ou effectué des transformations corporelles et/ou biologiques pour ressembler le plus possible à une femme.

Les transgenres ont exprimé leur propension à exprimer leur féminité sous diverses attitudes vestimentaires, sociales ou sexuelles et à avoir recours à des transformations corporelles souvent superficielles. Ces transgenres ont une forme de féminité « naturelle » qui les rend plus visibles, les exposent davantage ainsi que leurs partenaires et leurs familles au risque de stigmatisation et donc de violences verbales et/ou physiques. Les transgenres sont ceux qui ont été le plus souvent victimes de violences dès l’enfance avec des abus sexuels, au sein de l’école, de leurs quartiers, ou de la part de proches. Ils sont souvent éloignés ou expulsés de leurs domiciles familiaux. Beaucoup d’entre eux ont peu de signes extérieurs de masculinité, et ont du mal à dissimuler leur féminité naturelle et/ou à contracter des unions hétérosexuelles. Souvent classés dans la catégorie HSH, les transgenres affichent leur féminité, sont très sollicités et font l’objet de convoitises qui attisent les conflits entre pairs. Ils ont souvent des partenaires sexuels multiples avec lesquels ils adoptent des attitudes de dépendance et de soumission. Ils ont de nombreuses offres de transactions sexuelles et ont parfois du mal à négocier l’utilisation du préservatif car généralement « passifs », même si dans certaines circonstances ils peuvent devenir « actifs ». Tous ces facteurs augmentent leur vulnérabilité et accroissent leurs risques visà-vis du VIH.

## Conclusion

Ces résultats confirment la réalité d’une catégorie transgenre souvent classée dans le groupe des HSH, venant de toutes les catégories sociales et régions du Sénégal. Les transgenres cumulent dès le jeune âge des vulnérabilités familiales, psychologiques, sociales et sexuelles qui continuent à les exposer au risque VIH dans un contexte nécessitant le déploiement de stratégies plus adaptées à leurs réalités.

# Research article: 258 - Facteurs associés à la mortalité des personnes vivant avec le VIH sous ARV au Service des maladies infectieuses et tropicales de l’Hôpital national Donka, Guinée, 2016-2020

**Published:** 2024-02-23

**Authors:** Mamadou Saliou SOW, Mamadou Dian SOW, Nicolas MEDA, Idrissa SANOU, Dramane KANIA

**Affiliations:** 1 Hôpital national Donka, Centre hospitalier universitaire de Conakry, Guinée; 2 Agence nationale de sécurité sanitaire (ANSS), Guinée; 3 Programme burkinabé de formation en épidémiologie de terrain (BFELTP), Université Ouagadougou 1 Joseph Ki-Zerbo, Ouagadougou, Burkina Faso

## Introduction

Le VIH/sida demeure un problème de santé publique en Guinée où il représente une cause de morbi-mortalité avec un impact négatif au plan humain et sur le plan du développement socio-économique du pays.

## Méthodes

Cette étude transversale à visée analytique a examiné les dossiers médicaux de 400 PvVIH sous ARV (patients vivant avec le VIH sous antirétroviraux) suivis sur 5 ans dans le Service des maladies infectieuses et tropicales de l’Hôpital Donka. Les analyses statistiques ont été effectuées à l’aide d’Epi Info* 7.2.4. Un modèle de régression logistique a été utilisé pour identifier les variables indépendamment associées à la mortalité des PvVIH sous ARV. Les variables explicatives associées avec une valeur p

## Résultats

Sur 400 dossiers éligibles, 118 (29 %) sont décédés. L’âge médian (IIQ) des patients était de 40 ans (33-48). La tranche d’âge de 35-44 ans (34 %) était la plus représentée. Le sexe féminin (52 %) était majoritaire avec 68 % de mariés. Le VIH-1 était observé chez 99,75 % des patients et un poids corporel ≤ 40 kg chez 63,93 % des patients. Cependant, 85,75 % des patients étaient au stade IV de l’OMS avec un taux d’hémoglobine ≤ 10 g/dL chez 54,50 % des patients et un taux de CD4 ≤ 50 cellules/mm^3^ chez 41,89 % des patients. L’association TDF + 3TC + EFV était utilisée chez 97,75 % des patients. Le régime RHZE était utilisé chez 63 % des patients.

## Conclusion

La mortalité des PvVIH reste élevée dans le Service des maladies infectieuses et tropicales (SMIT) de l’hôpital Donka malgré l’introduction des ARV. Les patients décédés étaient caractérisés par un stade avancé de l’infection par le VIH avec un faible taux de CD4. Le faible poids corporel et le taux de CD4 effondré étaient les facteurs associés à la mortalité des PvVIH sous ARV au SMIT/Donka de 2016 à 2020.

# Research article: 220 - Quelle place pour le « one-stop shop » dans la cogestion Tuberculose/VIH dans un contexte de prestations de soins différenciés (PSD) ?

**Published:** 2024-02-23

**Authors:** Thierno Chérif SY, Souleymane SAGNA, Yaya BALDE, Ibrahima SARR, Adama DIALLO, Mahamady SOUANÉ, Maimouna COLY, Mamadou NDOUR, Abdoulaye SAGNA

**Affiliations:** 1 Unité de traitement ambulatoire du Centre de santé de Kolda, Sénégal; 2 District sanitaire de Kolda; 3 Région médicale de Kolda; 4 Conseil national de lutte contre le sida (CNLS), Sénégal

## Description du problème

Au niveau du district sanitaire de Kolda, les patients étaient traités séparément pour la tuberculose (TB) et le VIH. Les patients TB testés séropositifs au VIH par le chargé de traitement de la TB (CDT) sont orientés vers le service de prise en charge du VIH pour le traitement antirétroviral (ARV) et inversement. Les conséquences sont des rendez-vous multiples, des files d’attente longues entraînant la lassitude des patients et des perdus de vue.

Pour améliorer les indicateurs de la co-gestion TB/VIH, le district a opté pour une application stricte des directives de l’OMS en matière de collaboration pour la lutte contre les deux maladies en mettant en œuvre la stratégie du « one-stop shop », où le même soignant fournit sous le même toit tous les services TB-VIH, ceci à partir de janvier 2019.

## Activités réalisées

- Une note de service instaurant la délégation des tâches de dispensation ARV au CDT a été signée par le médecin chef de district (MCD).
- Le CDT et son assistant ont bénéficié d’une formation sur la prise en charge du VIH suivie d’un stage pratique au niveau de l’unité de traitement ambulatoire. Ils proposent et réalisent le test de dépistage rapide du VIH à tous les patients tuberculeux, et procèdent à la confirmation au laboratoire.
- Les cas de tuberculose chez les PvVIH leur sont référés pour la prise en charge antituberculeuse.
- Ils initient le co-traitement (ARV et antituberculeux) ainsi que la prophylaxie au cotrimoxazole pour tous les nouveaux patients diagnostiqués et ceux référés du service social.
- Ils assurent le suivi mensuel des deux affections selon une approche différenciée jusqu’à la guérison de la tuberculose.
- Ils demandent la mesure de la charge virale aux patients éligibles.
- Ils participent aux réunions du comité médical technique.
- Ils réfèrent les patients au service social à la fin du traitement antituberculeux pour suivi unique du VIH.

Une enquête de satisfaction a été réalisée auprès des patients avant et après le début de la stratégie.

## Résultats

- 116 clients suivis dont 59 femmes et 57 hommes de janvier 2020 à juin 2022.
- 100 % de réalisation du GeneXpert chez les co-infectés.
- Diminution du temps d’attente des clients passant de 2 heures à 25 minutes.
- Succès thérapeutique amélioré, passant de 68 % en 2018 à 86 % en 2021.
- Réduction des perdus de vus, passant de 35 à 8 (2018 vs 2021).
- Réduction de l’exposition des PvVIH à la tuberculose par une séparation des salles d’attente.
- Cependant, pas de réduction notée des décès, avec persistance du retard au diagnostic des deux affections.
  Leçons apprises et étapes futures.
- La qualité de la co-gestion TB/VIH est améliorée par une meilleure intégration des services.
- La prise en compte des préoccupations des patients améliore leur adhésion au traitement.
- Le « one-stop shop » peut être adopté comme un modèle PSD pour la co-gestion TB/VIH.

# Research article: 88 - Séroprévalence du SARSCoV-2 chez les professionnels de santé dans les régions de Kaolack et de Ziguinchor après la deuxième vague Covid-19 au Sénégal

**Published:** 2024-02-23

**Authors:** Adja Khady SÈNE, Noël Magloire MANGA, Habibou SARR, Baba SOKHNA, Ibrahima BÂ, Daouda DIOUF, Vic ARENDT, Henri GOEDERTZ

**Affiliations:** 1 Institut de recherche en santé, de surveillance épidémiologique et de formation (IRESSEF), Sénégal; 2 Laboratoire, Hôpital de la Paix, Ziguinchor, Sénégal; 3 Laboratoire régional de Kaolack, Sénégal; 4 Enda Santé, Sénégal; 5 Université de Luxembourg, Luxembourg

## Objectifs

Les études de séroprévalence du SARS-CoV-2 jouent un rôle crucial dans la détection des personnes en contact avec le virus autant dans la population générale que dans certains sous-groupes, notamment les professionnels de santé.

L’objectif de cette étude est de déterminer la séroprévalence de la SARS-CoV-2 immunoglobuline G (IgG) chez les professionnels de santé dans les régions de Kaolack (région carrefour et de transit) et de Ziguinchor (région transfrontalière) après la deuxième vague pandémique au Sénégal.

## Méthodes

C’est une étude transversale en collaboration entre ENDA Santé, l’Université de Luxembourg, les régions médicales et l’IRESSEF. La population d’étude est composée de tous types de personnel travaillant dans les structures sanitaires allant du vendeur de tickets à l’agent administratif en passant par les chauffeurs, les agents de sécurité et tout le personnel technique et médical. À partir d’un prélèvement sanguin sur tube EDTA chez des patients consentants, le prétraitement a été effectué dans les laboratoires régionaux et le plasma envoyé à l’IRESSEF. Les tests sérologiques ont été réalisés avec le kit Anti-SARS-CoV-2 ELISA (IgG) des laboratoires EUROIMMUN” dont le principe repose sur la détection des anticorps anti-SARS-CoV-2 du domaine S1 de la protéine Spike.

## Résultats

Nous avons inclus 497 professionnels de santé dont 266 de la région de Ziguinchor et 231 de la région de Kaolack avec des médianes d’âge respectives de 36 et 35 ans. Les participants ayant reçu le vaccin contre le coronavirus sont au nombre de 403 soit un pourcentage de 96,6 %. Une documentation d’infection antérieure à Covid-19 a été notée chez 89 patients sur 100 testés (soit 20,12 % de la population).

Sur le total des participants, 417 sont revenus positifs soit une séroprévalence de 83,9 % dont 345 vaccinés (soit 82,7 %) et 27 patients avec un test Covid-19 positif (soit 30,3 %) et les mêmes étant vaccinés. Mais d’après les données sur lesquelles nous avons travaillé, le personnel médical et les agents de santé qui sont plus en contact avec les malades représentent la majeure partie des séropositifs (soit 13,6 %). Nous avons aussi observé une légère différence géographique, soit 200 à Kaolack et 217 à Ziguinchor (47,9 et 52 %).

## Conclusion

Ces données mettent en lumière le risque élevé d’infection chez les professionnels de santé. Cependant, la présente étude ne permet pas de déterminer la proportion des infections acquises dans les structures sanitaires en fonction du type de personnel y travaillant. Par ailleurs, ces résultats représentaient un véritable reflet du rôle des vaccins sur la prévention.

En outre, ces données pourraient nous orienter vers des études approfondies, notamment une exploration des facteurs associés à une sérologie positive, qu’elle soit due à la vaccination ou à l’infection, afin de bien suivre la progression de la pandémie de la Covid-19 au Sénégal.

# Research article: 32 - Des services différenciés face aux contraintes structurelles de la prise en charge des enfants et adolescents vivant avec le VIH en contexte décentralisé au Sénégal

**Published:** 2024-02-23

**Authors:** Bernard TAVERNE, Gabrièle LABORDE-BALEN, Khoudia SOW, et le groupe ETEA-VIH

**Affiliations:** 1 TransVIHMI, Université de Montpellier, INSERM, IRD, Montpellier, France; 2 Centre régional de recherche et de formation à la prise en charge clinique de Fann (CRCF), Dakar, Sénégal

## Objectifs

Au Sénégal, en contexte décentralisé – rural – l’intégration de la prise en charge pédiatrique du VIH dans le système de soins reste difficile. En 2015, une étude conduite sur l’ensemble du pays (hors région de Dakar) a révélé que 64 % des enfants (0-19 ans) recevant des ARV présentaient un échec virologique. Dans le cadre du projet ETEA-VIH (Échec thérapeutique chez les enfants et adolescents vivant avec le VIH en contexte décentralisé [ANRS 12421]), nous avons entrepris une analyse des contraintes structurelles.

## Matériels et méthodes

Des enquêtes qualitatives (entretiens et observations) ont été menées en 2020 dans 4 hôpitaux régionaux et 11 centres de santé, de 11 régions du pays. Des entretiens et des observations ont concerné 87 enfants/adolescents vivant avec le VIH (EAvVIH), 94 parents/tuteurs et 47 acteurs de santé. L’organisation des dispositifs de prise en charge a fait l’objet d’une analyse spécifique.

## Résultats

Le dispositif de prise en charge repose souvent sur quelques personnes (un médecin ou un infirmier, un assistant social et un médiateur communautaire) qui cumulent un grand nombre d’activités supplémentaires; les consultations médicales sont souvent assurées par un infirmier, parfois un assistant social, le médecin étant souvent pris par des tâches administratives ou changeant périodiquement de lieu d’affectation. Malgré diverses formations biomédicales, la responsabilité de la prise en charge des EAvVIH repose souvent sur les médiateurs généralement VIH+, plus stables et qui ont une meilleure connaissance de l’histoire médicale et sociale des patients. Les professionnels de santé les plus jeunes maîtrisent moins bien que les anciens les connaissances sur le VIH et n’ont pas les mêmes engagements militants. Dans le flot des consultations générales, ils peinent à instaurer une relation personnalisée avec les EAvVIH. Les ruptures d’approvisionnement en ARV, notamment pédiatriques, sont fréquentes ce qui conduit à découper des médicaments pour adultes. L’accès à la charge virale reste limité : dans 8 sites (dont un hôpital régional), moins de 4 patients sur 10 avaient un résultat de charge virale en 2020. Les assistants sociaux et médiateurs expliquent avoir réduit les activités d’accompagnement communautaires faute de financement. De nombreuses structures ré-introduisent le paiement de certaines prestations, auparavant gratuites pour les PvVIH, afin de maintenir leur équilibre financier, alors que les dispositifs de couverture sanitaire tels que la CMU sont rarement opérants.

## Conclusion

Les défaillances structurelles du système de soins, certaines communes à tous les sites, d’autres particulières, limitent l’efficacité de la prise en charge décentralisée des EAvVIH. Les identifier précisément permet de proposer des réponses différenciées et adaptées à chaque site pour maintenir une offre de soin de qualité.

# Research article: 208 - Dépistage des lésions pré-cancéreuses par le test PVH et l’IVA dans trois régions du Sénégal : résultats préliminaires

**Published:** 2024-02-23

**Authors:** Fadimatou Bintou THIAM, Dianké SAMATÉ, Aboubacry DRAMÉ, Yaye Coumba COULIBALY, Oumy DIOP-DIONGUE, Blandine BOURGOIN, Marie Edouard FAYE, Mamadou DIOP, Marieme DIALLO, Daouda CISS, Halimatou DIOPNDIAYE, Yacine DIENG

**Affiliations:** 1 Laboratoire de bactériologie et de virologie, Centre hospitalier national universitaire Aristide Le Dantec, Dakar, Sénégal; 2 Agence nationale de la statistique et de la démographie (ANSD), Sénégal; 3 Institut Joliot-Curie du cancer, Centre hospitalier national universitaire Aristide Le Dantec, Dakar; 4 Clinton Health Initiative Access (CHAI); 5 Clinique gynécologique et obstétricale, Centre hospitalier national universitaire Aristide Le Dantec, Dakar; 6 Hôpital général Idrissa Pouye, Dakar; 7 Division de lutte contre les maladies non transmissibles, Ministère de la Santé, Dakar

## Introduction

Le cancer du col de l’utérus est dû à des infections persistantes par des papillomavirus humains (PVH). Néanmoins il peut être évité grâce à des interventions de dépistage efficaces visant à détecter et à traiter les lésions précancéreuses du col avant qu’elles ne deviennent invasives. Notre objectif était de dépister les lésions précancéreuses du col en utilisant test de PVH et l’inspection visuelle du col après application à l’acide acétique (IVA).

## Méthodologie

Il s’agissait d’une étude prospective et descriptive réalisée entre mai et septembre 2022 dans le cadre d’une collaboration avec les services de gynécologie des régions de Dakar, Thiès et Fatick, l’Institut Joliot-Curie et le laboratoire de bactériologie-virologie de l’hôpital Aristide Le Dantec. Dans cette étude, des femmes âgées de 30 à 49 ans ont été incluses de manière exhaustive après un consentement libre et éclairé. Une inspection visuelle à l’acide acétique du col et un prélèvement sur solution PreservCyt pour le test de PVH ont été réalisés chez chaque participante. La recherche des génotypes 16,18 et autres PVH à haut risque a été faite à l’aide du test Cobas 4800 HPV. Une biopsie a été effectuée chez toutes participantes dont l’IVA ou le test PVH était positif. Les données ont été saisies sur le logiciel Filemaker Pro et l’analyse statistique a été effectuée par les logiciels Excel et Epi Info.

## Résultats

Au total, 2886 femmes ont été incluses dans 10 sites avec une moyenne d’âge de 39,9 ans (± 5,3 ans). Le nombre de femmes recrutées par site variait entre 257 au Centre de santé de Dominique et 330 à la Clinique gynécologie de l’hôpital Aristide Le Dantec. Le taux de positivité global du test PVH était de 13,9 % (400/2886) et variait entre 10,5 % (27/257; CS de Dominique) et 15,5 % (46/295; Institut Joliot-Curie). Le génotypage viral a trouvé PVH-16 dans 32 cas (8,0 %), PVH-18 dans 21 cas (5,2 %), PVH-16-18 dans 3 cas (0,8 %) et les autres PVH à haut risque oncogène dans 344 cas (86,0 %).

L’IVA a été réalisée chez toutes les femmes avec un taux de positivité de 10,6 % (305/2886) et seules 1,6 % (45/2886) des patientes étaient positives aux deux tests.

Un total de 171 patientes ont bénéficié de la biopsie avec 43 (25,2 %) cols normaux, 110 (64,3 %) CIN1, 17 néoplasies intra-épithéliales de haut grade (CIN2+) soit 9,9 %, et 1’adénocarcinome *in situ* (AIS) soit 0,6 %.

De façon surprenante, 9 patientes à test PVH négatif et 7 patientes négatives à l’IVA présentaient des lésions de haut grade.

Le statut VIH était connu pour 1863 femmes dont 6 étaient positives, soit une prévalence de 0,3 %.

## Conclusion

Ces résultats préliminaires confirment l’intérêt des tests IVA et PVH pour le dépistage des lésions précancéreuses et la prédominance du portage des autres types de PVH à haut risque oncogène chez les femmes au Sénégal.

# Research article: 184 - Évaluation de la prise en charge des enfants vivant avec le VIH suivis à l’hôpital régional de Thiès, Sénégal (2004-2020)

**Published:** 2024-02-23

**Authors:** Pauline DIOUSSE, Daouda THIOUB, Mariama BAMMO, A. Dela-Dem Tevi LAWSON, Haby DIONE, Fatou Seck SARR, Sylvie Audrey DIOP

**Affiliations:** 1 UFR Santé, Université de Thiès, Sénégal; 2 Dermatologie, Hôpital régional de Thiès, Sénégal; 3 Infectiologie, Hôpital de Tivaouane, Sénégal; 4 Dermatologie, Hôpital de Tivaouane, Sénégal; 5 Dermatologie, Hôpital de Mbour, Sénégal

## Introduction

Après plusieurs années de suivi des enfants, face aux nouveaux défis et aux objectifs d’élimination du VIH chez les enfants, il s’avère nécessaire d’évaluer la prise en charge décentralisée des enfants. L’objectif général était de contribuer à l’évaluation de la prise en charge pédiatrique du VIH en milieu décentralisé.

## Matériel et méthode

Du 1^er^ janvier 2004 au 31 décembre 2020, soit 17 ans, était menée une étude rétrospective et descriptive. Étaient inclus tous les dossiers des enfants infectés par le VIH reçus dans le service.

## Résultats

Au total, 101 enfants étaient colligés. L’âge moyen était de 7,28 ± 3,70 ans. Il y avait une prédominance du sexe masculin (59,41 %).

Les parents des enfants séropositifs étaient vivants dans 60,47 % chez les mères et 55,81 % chez les pères. Concernant le statut sérologique, on enregistrait un taux de positivité maternelle de 67,44 %.

Plus de la moitié des enfants (86,67 %) étaient sous allaitement maternel exclusif. La toux (15,84 %), la candidose digestive (12,87 %) et la malnutrition (7,92 %) étaient les manifestations cliniques les plus représentées. Les enfants étaient diagnostiqués majoritairement au stade I (31,7 %) et au stade IV (26,7 %). Le VIH-1 était prédominant (97,03 %). Le taux moyen de CD4 était de 573 ± 571/mm^3^. La charge virale était réalisée chez 6 patients (5,94 %). Le taux moyen était de 1 726 827 ± 4 053 436 copies/ml. Le pourcentage d’enfants sous ARV était de 90,70 %. Un traitement de 1^re^ ligne avait été initié dans 90,48 %. Dix-neuf enfants (20 %) étaient passés à une 2^e^ ligne suite à un échec virologique. Quarante enfants soit 39,60 % étaient sous prophylaxie au cotrimoxazole et 13,86 % sous prophylaxie à l’isoniazide. 42,19 % étaient observants et 53,73 % respectaient les rendez-vous. L’annonce du statut était faite chez 64,10 %. Parmi les enfants 32,67 % étaient suivis, 27,72 % décédés, 25,74 % perdus de vue et 13,86 % transférés. La malnutrition aiguë sévère (54,54 %), la tuberculose (27,27 %) et la diarrhée chronique (27,27 %) étaient les principales causes de décès.

## Conclusion

Des efforts restent à faire dans la prise en charge des enfants séropositifs afin de l’améliorer.

# Research article: 183 - Les comorbidités au cours de l’infection au virus de l’immunodéficience humaine (VIH) à l’hôpital régional de Thiès, Sénégal (2009-2019)

**Published:** 2024-02-23

**Authors:** Pauline DIOUSSE, Daouda THIOUB, Aminata DEH, Mariama BAMMO, A. Dela-Dem Tevi LAWSON, Haby DIONE, Fatou Seck SARR, Sylvie Audrey DIOP

**Affiliations:** 1 UFR Santé, Université de Thiès, Sénégal; 2 Dermatologie, Hôpital régional de Thiès, Sénégal; 3 Infectiologie, Hôpital de Tivaouane, Sénégal; 4 Dermatologie, Hôpital de Tivaouane, Sénégal; 5 Dermatologie, Hôpital de Mbour, Sénégal

## Introduction

L’utilisation à large échelle et à long terme du traitement antirétroviral (TARV) a réduit considérablement la mortalité, entraînant l’augmentation de l’espérance de vie des Personnes vivant avec le VIH (PvVIH) et la survenue de comorbidités. Le but de ce travail était d’étudier les comorbidités chez les PvVIH.

## Matériels et méthode

Il s’agissait d’une étude rétrospective, descriptive sur une période de 10 ans (2009-2019). Étaient inclus tous les dossiers des PvVIH âgés de plus de 15 ans. Les variables étudiées étaient les aspects sociodémographiques, cliniques, paracliniques, évolutifs, thérapeutiques des comorbidités. La saisie et l’analyse des données étaient faites avec le logiciel Epi Info 2000 version 7.2.4.0.

## Résultats

Nous avons colligé 297 dossiers de PvVIH sur un total de 43 906 patients, soit une prévalence hospitalière de 6,76 %. L’âge moyen était de 44,52 ans [19-80] ans avec un sex-ratio F/H de 0,63. Parmi les comorbidités retrouvées, la dyslipidémie était de 41,82 % des cas suivie de l’hypertension artérielle (23,35 %), la lipodystrophie (22,54 %), l’obésité (16,75 %) et le diabète (6,6 %). L’insuffisance rénale était retrouvée dans 19,44 % des cas, l’atteinte hépatique dans 2,75 % des cas, l’AVC ischémique dans 4,57 % des cas.

## Conclusion

Des études ont montré que pour certains antirétroviraux, la longue durée de traitement entraînerait la survenue de comorbidités. Le dépistage de ces dernières doit être pris en compte dans le suivi des PvVIH afin d’améliorer leur qualité de vie dans le contexte de maladie chronique de l’infection par le VIH.

# Research article: 53 - Perspective d’introduction des traitements antirétroviraux injectables à longue durée d’action (ARV-LA) au Sénégal : un optimisme prudent

**Published:** 2024-02-23

**Authors:** Séverine CARILLON, Maïmouna DIOP, Gabrièle LABORDE-BALEN, Guillaume BRETON, Karim DIOP, Bara NDIAYE

**Affiliations:** 1 Expertise France; 2 Centre régional de recherche et de formation à la prise en charge clinique de Fann (CRCF), Dakar, Sénégal; 3 TransVIHMI, Université de Montpellier, INSERM, IRD, Montpellier, France; 4 Division de lutte contre le sida et les IST (DLSI), Dakar, Sénégal, ANRS, Expertise France; 5 Faculté de médecine, Université Cheikh Anta Diop, Dakar, Sénégal; 6 Solthis, Paris, France

## Contexte

Le traitement antirétroviral injectable à longue durée d’action (ARV-LA) peut offrir aux Personnes vivant avec le VIH (PvVIH) une alternative intéressante au traitement oral quotidien. La question de l’implémentation de ces traitements en contexte ouest-africain reste peu documentée. Cette communication vise à mettre en évidence les défis et conditions d’introduction des ARV-LA dans la vie sociale des PvVIH et dans les pratiques de prise en charge du VIH au Sénégal.

## Méthode

Une étude qualitative a été conduite en 2021-2022 dans 3 services de prise en charge du VIH à Dakar. Des entretiens semi-directifs ont été réalisés auprès de 42 PvVIH – 12 femmes, 30 hommes parmi lesquels 20 hommes ayant des rapports sexuels avec d’autres hommes (HSH) et 13 professionnels de santé (1 infirmier, 8 médecins, 2 assistantes sociales, 1 pharmacienne, 1 biologiste). Une analyse transversale de contenu a été réalisée.

## Résultat

Pour les PvVIH rencontrées, quel que soit le nombre d’années écoulées avec les traitements, la gestion quotidienne des ARV fait l’objet de routines. Pour autant, la majorité fait part d’une certaine lassitude. Les ARV injectables sont perçus comme une nouvelle opportunité de traitement, permettant de s’affranchir des contraintes associées au secret de la maladie qui les concernent tous. Ils garantiraient l’allègement d’un fardeau psychologique, logistique et social associé jusqu’ici aux comprimés et diminueraient la crainte du stigma. Cet enthousiasme manifesté a priori peut toutefois être nuancé par un rapport ambivalent aux injections (entre peur et efficacité) et est soumis à certaines conditions : pas d’effets secondaires, des expérimentations préalables sur autrui, l’aval des médecins. Par ailleurs, certaines contraintes liées à la médicalisation des soins sont à anticiper, parmi lesquels l’obligation de se rendre strictement tous les 2 mois à l’hôpital pour l’injection et, avec elle, l’enjeu du glissement de la question de l’observance thérapeutique à celle de l’observance des rendez-vous pour les injections. Les PvVIH auront dès lors à expérimenter un nouveau travail de gestion du suivi médical. La prise en charge globale des PvVIH sera quant à elle à repenser. Enfin, les résultats mettent en évidence à la fois certaines conditions propices à l’introduction des ARV-LA au Sénégal – expertise et expériences passées d’introduction d’innovations thérapeutiques – et des défis organisationnels importants – l’introduction des ARV-LA peut mettre le système de prise en charge face à des défis d’approvisionnement et de pérennisation.

## Conclusion

En mettant en évidence trois enjeux majeurs de l’introduction des ARVLA – simplification de la vie avec le VIH, remédicalisation et adaptation du système de prise en charge – les résultats de cette étude permettent d’éclairer le déploiement de programmes d’introduction des ARV-LA « dans la vraie vie » en contexte ouest-africain.
